# Transcriptomic and enzymatic analysis of peroxidase families at the early growth stage of halophyte ice plant (*Mesembryanthemum crystallinum* L.) under salt stress

**DOI:** 10.1186/s40529-024-00450-y

**Published:** 2025-01-21

**Authors:** Li-Ching Hsieh, Chia-Che Lee, Kai-Fu Zhang, Hui-Hsien Chang, Cheng-Hsun Li, Hsuan-Jung Huang, Hungchen Emilie Yen

**Affiliations:** 1 Graduate Institute of Genomics and Bioinformatics, National Chung Hsing University, Taichung, 40227 Taiwan; 2 Department of Life Sciences, National Chung Hsing University, Taichung, 40227 Taiwan; 3 Advanced Plant and Food Crop Biotechnology Center, National Chung Hsing University, Taichung, 40227 Taiwan; 4 Present address: Department of Biochemical Science and Technology, National Taiwan University, Taipei, 10617 Taiwan

**Keywords:** Ice plant, Halophyte, Hydrogen peroxide, Peroxidase, Transcriptome

## Abstract

Ice plant (*Mesembryanthemum crystallinum* L.) is a halophyte and an inducible CAM plant. Ice plant seedlings display moderate salt tolerance, with root growth unaffected by 200 mM NaCl treatments, though hypocotyl elongation is hindered in salt-stressed etiolated seedlings. Superoxide anion accumulation was prominent in cotyledons and primary leaves but decreased in root tissues over time, with no significant effect from salt treatment. Hydrogen peroxide levels initially surged in both control and salt-treated seedlings, with higher and more persistent accumulation in the salt-treated seedlings. The activities of H_2_O_2_-scavenging ascorbate-glutathione cycle enzymes ascorbate peroxidase (APX), monodehydroascorbate reductase, and dehydroascorbate reductase increased, while guaiacol-dependent peroxidase activity decreased and catalase activity showed no change, indicating APX activity as the primary response to salt stress. Salt-induced APX activities were detected mainly in the microsomal fraction for light-grown seedlings and the cytosolic fraction for etiolated seedlings, highlighting plastids as the primary site of ROS accumulation under salt stress. An RNA-seq analysis of etiolated seedlings revealed about 8% unigenes showing more than a four-fold change in expression after a 6-h 200 mM NaCl treatment. GO enrichment analysis indicated that differentially expressed genes (DEGs) with increased transcript abundance were associated with ion transport, antioxidant activity, and stress responses, while DEGs with decreased transcript abundance were linked to metabolic and biosynthesis processes such as ribosomal protein synthesis and cell wall formation. This indicates that salt stress hinders growth but enhances ion homeostasis and stress response mechanisms. The expression of all eight *APX* genes were induced by a 48-hour salt treatment, with varying expression patterns. For class III peroxidase family, 14 out of 53 identified unigenes qualified as DEGs. The time-course expression patterns revealed that the transcript levels of *McPrx4.1*, *McPrx12.1*, and *McPrx12.3* increased, while *McPrx60.3* decreased. These findings highlight the distinct roles of class III peroxidases in balancing plant growth and stress responses, advancing our understanding of the mechanisms behind salt tolerance in halophytes. This study comprehensively analyzed changes in gene expression, antioxidant enzyme activity, and ROS accumulation in ice plant seedlings. Unveiling these responses will advance our understanding of the growth–stress balance in the intrinsic salt tolerance in halophytes.

**Supplementary Information:**

The online version contains supplementary material available at 10.1186/s40529-024-00450-y.

## Background

Environmental stresses such as water deficits, salinity, extreme temperatures, and heavy metals induce oxidative stress, leading to overproduction of reactive oxygen species (ROS) like singlet oxygen (^1^O_2_), superoxide (O_2_^.^^−^), hydrogen peroxide (H_2_O_2_), and hydroxyl radicals (HO·). These ROS, generated primarily through electron transport chains and peroxisomal respiration, can damage cellular components but also serve as signaling molecules regulating stress-responsive gene networks (Dumanović et al. 2021; Waszczak et al. 2018). Both enzymatic and non-enzymatic antioxidant systems regulate cellular ROS levels. Superoxide radicals generated by electron transport chains in chloroplasts and mitochondria, as well as by NAD(P)H oxidase in the plasma membrane (Bhattacharjee 2005) are converted to H_2_O_2_ by superoxide dismutases (SODs): Cu/ZnSOD and FeSOD in the chloroplast stroma and, MnSOD in the mitochondrial matrix. The ascorbate-glutathione cycle, involving ascorbate peroxidase (APX), monodehydroascorbate reductase (MDHAR), dehydroascorbate reductase (DHAR), and glutathione reductase (GR), is a key pathway for scavenging H_2_O_2_ in the cytoplasm, chloroplasts, and mitochondria (Gill and Tuteja 2010). Chew et al. (2003) identified nine *APX*, five *MDHAR*, five *DHAR*, and two *GR* genes in the Arabidopsis genome, and the gene products APX, MDHAR, and GR can be targeted simultaneously into mitochondria and chloroplasts. Ascorbate, glutathione, and other non-enzymatic antioxidants, such as carotenoids, tocopherols, phenolics, and polyols, directly scavenge ROS and support antioxidant enzymes. In the halophyte ice plant, major non-enzymatic antioxidants include pinitol, betacyanins, and mesembryanthin (Ibdah et al. 2002; Motohashi 2014).

The common ice plant (*Mesembryanthemum crystallinum*) is a model plant used to study salt and drought tolerance. Ice plant can adapt to many environmental stressors as a consequence of its effective antioxidant systems. Salt-stressed ice plants exhibit significant increases in SOD activity, with an initial rise in chloroplastic FeSOD activity preceding the onset of CAM, followed by increased cytosolic Cu/ZnSOD and mitochondrial MnSOD activities, suggesting the chloroplast is the primary site of salt-induced oxidative stress (Miszalski et al. 1998). In 5-week-old plants, *Cu/ZnSOD* expression increased after 2 days of high salt or high light treatments, serving as an indicator of oxidative stress (Hurst et al. 2004). Many studies have worked on ice plant SODs, but only two studies focusing on the peroxidases of ice plant were found in the literature. The enzyme APX is a class I heme-containing peroxidase that uses ascorbate as the electron donor and H_2_O_2_ as the acceptor, and distinct APX isoforms are found in chloroplasts, mitochondria, peroxisomes, and the cytosol (Li 2023). A small gene family encodes APX genes in Arabidopsis, including three cytosolic APXs (*AtAPX1*, *2*, and *6*), three peroxisomal APXs (*AtAPX3*, *4*, and *5*), and two chloroplastic APXs (stromal APXs [*sAPX*] and thylakoid membrane-bound APXs [*tAPX*]). Class II peroxidases are found in fungi and are involved in lignin degradation. Class III peroxidases are found only in plants, where they form large multigenic families with 73 genes identified in Arabidopsis and 138 identified in rice (Passardi et al. 2007). These peroxidases localize in the apoplast or vacuole and catalyze the reduction of peroxide by oxidizing a wide variety of substrates, such as phenolic compounds, lignin, plant growth hormones, or secondary metabolites. Common examples include horseradish peroxidase, peanut peroxidase, and soybean peroxidase. Nonheme peroxidases, including glutathione peroxidase and thioredoxin-dependent peroxidase, are also key H_2_O_2_-scavenging enzymes (Sevilla et al. 2015).

Class III peroxidases play crucial roles throughout the plant life cycle, performing functions in H_2_O_2_ scavenging, lignification, cell elongation, cell wall metabolism, pathogen defense, and stress response (Passardi et al. 2004). They can also catalyze the reverse reaction, generating ROS molecules, such as H_2_O_2_ and singlet oxygen. Peroxidase activity is monitored by transferring hydrogen atoms and electrons from organic reducing agents, such as guaiacol, to H_2_O_2_ and measuring the resulting formation of water and oxidized organic substrates (MacAdam et al. 1992a). Based on the results obtained by MacAdam et al. (1992b), the activity of guaiacol-dependent peroxidase (GPX) is suitable for detecting the cationic isoforms of apoplastic peroxidase, i.e., class III peroxidases. Class III peroxidases are plant-specific, and their evolution is related to the emergence of land plants, the subsequent increase in the number of isoforms evolving to adapt to a wide range of substrates, and the functional redundancy arising with the complexity of these organisms (Passardi et al. 2007). As a result, many class III peroxidase-encoding genes (*Prx*), often more than one hundred, are found in the genomes of highly evolved plants (Bakalovic et al. 2006).

Class III peroxidases are involved in lignin synthesis and cell wall crosslinking, enhancing plant defense (Almagro et al. 2009). In Arabidopsis, *AtPrx30*, *AtPrx34*, and *AtPrx69* were linked to lignification during hypocotyl elongation (Irshad et al. 2008), while *AtPrx03* improved drought and salt stress tolerance by increasing fresh weights in overexpression lines and reducing them when expression was suppressed (Llorente et al. 2002). Overexpression of *GsPRX09* in wild soybean enhanced salt stress tolerance by increasing fresh weight, relative water content, and root length (Jin et al. 2019). Class III peroxidases also oxidize indole-3-acetic acid (IAA) via mechanisms involving H₂O₂ or O₂ (Gazaryan et al. 1996). Transgenic Arabidopsis overexpressing *Prx01* from zucchini (*Cucurbita pepo*) showed increased IAA oxidase activity and reduced IAA levels, while elevated IAA levels were observed in *Prx01* silencing lines, linking this peroxidase to auxin-mediated hypocotyl elongation (Cosio et al. 2009). In this report, we analyzed ice plant transcriptomes obtained from 3-day-old seedlings and found that 8 and 53 assembled transcripts were annotated as *APX* and *Prx* members, respectively. The expressions of *APX* and selected *Prx* members were then analyzed along with the salt-induced changes in ROS accumulation and enzyme activity.

The salt tolerance of the ice plant increases with development, reaching a peak in the adult stage due to mechanisms including a fully operational CAM pathway, fully developed epidermal bladder cells (EBCs) for Na^+^ compartmentation and herbivore deterrence (Moog et al. 2023), and the accumulation of compatible solutes for osmoregulation and ROS protection. These adaptations enable tolerance to NaCl levels up to 500 mM, exceeding seawater salinity (Bohnert and Cushman 2000). Before the draft genome was published (Shen et al. 2022), *de novo* transcriptome assembly revealed salinity-induced differentially expressed genes (DEGs) in roots, EBCs, and guard cells, highlighting tissue-specific responses and identifying salt tolerance genes for crop improvement (Tsukagoshi et al. 2015; Oh et al. 2015; Barkla et al. 2018; Kong et al. 2020). Although seedlings lack a functional CAM cycle and fully developed EBCs (Jou et al. 2007), they tolerate 150 mM NaCl, suggesting intrinsic stress tolerance mechanisms even at early stages (Bohnert and Cushman 2000). Our studies demonstrated that ice plant seedlings actively respond to increased Na^+^ flux by secreting Na^+^ through root surfaces or relocating it to leaf stomates and bladder cells, similar to mechanisms observed in adult plants (Chiang et al. 2016).

In this study, we evaluated the initial response of ice plant seedlings to salt-induced oxidative stress by analyzing ROS accumulation, antioxidant enzyme activities, and transcriptome profiling to understand how seedlings mitigate oxidative stress under high-saline conditions. We focused on changes in the expression of *APX* family genes and selected *Prx* family members to elucidate the complex roles of these peroxidases in oxidative stress responses.

## Materials and methods

### Materials

Sterilized ice plant (*Mesembryanthemum crystallinum* L.) seeds were sown in 1X Murashige and Skoog basal medium with vitamins (M519, *Phyto*Technology Laboratories, USA) and maintained in a vertical position at 25^o^C under illumination 16 h 50 µmol m^− 2^ s^− 1^ light/8 h dark for 5 days (light-grown seedlings) or continuous darkness at 25^o^C for 3 days (dark-grown/etiolated seedlings). During the salt treatment time, agar plates were placed at an angle of inclination of 10^o^ and control (liquid MS medium) or salt (MS plus 200 mM NaCl) were added directly to plates to cover the lower part of the roots, but not the upper part of seedlings as described by Chiang et al. (2016). Seedlings were collected, washed in distilled water, and blotted dry at different time points up to 48 h. Each treatment was repeated at least three times and three to five seedlings were pooled per replicate.

### Methods

### Superoxide radical staining

Ice plant seedlings were immersed in nitroblue tetrazolium (NBT) (N6876, Sigma-Aldrich, USA) solution in 0.01 M potassium phosphate buffer (pH 7.8) containing 0.1% NBT and 0.01 M NaN_3_. Seedlings were infiltrated under vacuum for 2 min and incubated under dark for 90 min. Superoxide radical was visualized as a blue color produced by NBT precipitation. After infiltration, dark- and light-grown stained seedlings were stored in 0.01 M potassium phosphate buffer and 95% ethanol for chlorophyll clearing, respectively, until photographs were taken.

### Hydrogen peroxide staining

Ice plant seedlings were immersed in 3,3’-diaminobenzidine (DAB) (D5637, Sigma-Aldrich, USA) in 0.01 M potassium phosphate buffer (pH 7.8) containing 0.125% DAB and 0.01 M NaN_3_. Seedlings were infiltrated under vacuum for 2 min and incubated under dark for 90 min. Hydrogen peroxide was visualized as a brown color due to DAB polymerization. After infiltration, dark- and light-grown stained seedlings were stored in 0.01 M potassium phosphate buffer and 95% ethanol, respectively, until photographs were taken. For quantification of H_2_O_2_ contents, the stained seedlings were ground in liquid nitrogen and homogenized in 99.99% dimethyl sulfoxide (DMSO, Fisher Scientific, USA). The mixtures were centrifuged (10,000 x*g*, 10 min) and the absorption of supernatants at 450 nm were determined using a spectrophotometer (DS-11, DeNovix, USA). H_2_O_2_ concentrations were obtained using standard curve of 0, 0.01, 0.025, 0.05, and 0.1 mM H_2_O_2_ dissolved in 99.99% DMSO containing 0.125% DAB.

### Activity of APX, MDHAR, DHAR, GR and CAT

Seedlings (0.5 g) was extracted in a prechilled mortar and pestle with 0.8 mL ice-cold extraction buffer containing 50 mM potassium phosphate buffer, pH 7, 0.1 mM EDTA, and trace insoluble polyvinylpolypyrrolidone (PVPP, Sigma, USA). Homogenized tissue was centrifuged at 13,000 x*g* for 10 min at 4^o^C and the supernatant was used as enzyme extract for ascorbate peroxidase (APX), monodehydroascorbate reductase (MDHAR), dehydroascorbate reductase (DHAR), and glutathione reductase (GR) and catalase (CAT). The activities of APX and DHAR were assayed based on Nakano and Asada (1981). APX activity was monitored spectrophotometrically by following the H_2_O_2_-dependent oxidation of ascorbate at 290 nm, using an extinction coefficient of 2.8 mM^− 1^ cm^− 1^. The assay mixture contained 50 mM potassium phosphate buffer, pH 7, 0.1 mM EDTA, 0.5 mM ascorbate. The reaction was initiated by adding 100 µL freshly prepared 0.1 mM H_2_O_2_ (final concentration) in a total volume of 1 mL enzyme assay mixture. The assay mixture of DHAR contained 0.1 mM EDTA, 2.5 mM GSH (glutathione) and 1 mg mL^− 1^ DHA (dehydroascorbate). In the absence of enzyme extract as the blank, the increase in absorbance at 265 nm resulted from the non-enzymatic reduction of DHA by GSH. After adding enzyme extract, the change in 265 nm was subtracted from the blank value to calculate the DHAR activity (µmol ascorbate consumed min^− 1^). The activity of MDHAR was assayed based on Hossain et al. (1984). The assay mixture contained 0.2 mM NADH and 2.5 mM ascorbate and the reaction was initiated by adding 1.4 unit mL^− 1^ ascorbate oxidase. The oxidation of NADH was monitored at 340 nm. The activity of GR was assayed based on Carlberg and Mannervik (1985). The assay mixture contained 2 mM EDTA and 2.2 mM GSSG (glutathione disulfide) and the reaction was initiated by adding 0.15 mM NADPH to measure the oxidation of NADPH at 340 nm. The extinction coefficient of NADH/NADPH is 6.22 mM^− 1^ cm^− 1^. The activity of CAT was assayed based on Hadwan (2018). CAT activity was monitored by the rate of H_2_O_2_-dependent oxidation of Co^2+^ to Co^3+^ in the presence of bicarbonate. The detection of green colored carbonato-cobaltate (III) complex was performed at 440 nm in the room temperature.

### Activity of guaiacol peroxidase (GPX)

Seedlings (0.5 g) was extracted in a prechilled mortar and pestle with 0.8 mL ice-cold extraction buffer containing 50 mM potassium phosphate buffer (pH 5.8), 0.8 M KCl, and trace insoluble polyvinylpolypyrrolidone (PVPP, Sigma, USA) to extract both soluble and ionically bound peroxidase. Homogenized tissue was centrifuged at 13,000 x*g* for 10 min at 4^o^C and the supernatant was assayed for peroxidase activity based on MacAdam et al. (1992a). Guaiacol (2-methoxyphenol) is used as reducing agent of enzyme assay and guaiacol is oxidized to a dark orange compound called tetraguaiacol. GPX activity was monitored spectrophotometrically by following the H_2_O_2_-dependent oxidation of guaiacol at 470 nm, using an extinction coefficient of 26.6 mM^− 1^ cm^− 1^. One hundred µL crude extract were added to 660 µL reaction buffer (16.5 mM potassium phosphate buffer, pH 5.8) containing 7 mM guaiacol. The reaction was initiated by adding 240 µL freshly prepared 12 mM H_2_O_2_ (final concentration) in a total volume of 1 mL enzyme assay mixture. Total soluble protein of crude extract was measured using the Bio-rad protein assay dye reagent. The specific activity of GPX was calculated as changes in 470 nm within 5 min and expressed as µmol guaiacol consumed min^− 1^ mg protein^− 1^.

### Separation of soluble and microsomal fractions

Freshly collected seedlings (about 10 g) were immediately ground at room temperature with an equal volume of extraction buffer containing 50 mM Tris-HCl (pH 7.5), 10 mM KCl, 1 mM EDTA, 0.1 mM MgCl_2_, and 8% sucrose. Homogenized tissue was centrifuged at 3000 x*g* for 20 min, filtered through Miracloth, and the resulting product being referred to as crude extract. The crude extract was then centrifugated at 100,000 x*g* for 1 h (Optima L-100 K Ultracentrifuge, Beckman Coulter). The post-centrifugation supernatant and pellet was referred to as soluble and microsomal fraction, respectively. The activity of APX was determined in crude extract, soluble, and microsomal fractions. Total soluble protein of crude extract and soluble fraction was measured using the Bio-rad protein assay dye reagent. Total microsomal protein was measured using Pierce™ BCA protein assay kit. The specific activity of APX was calculated as decrease of 290 nm within 5 min and expressed as µmol ascorbate consumed min^− 1^ mg protein^− 1^.

### RNA extraction and quantitative RT-PCR

Total RNA was extracted from 15 seedlings using 1 mL TRIzol reagent (Invitrogen) according to the manufacturer’s instruction. The reverse transcription reaction was performed in 1 µg total RNA and 1 µL of 10 µM random hexamer using ImProm-II Reverse Transcriptase (Promega). The PCR reaction was performed in 10 µM gene-specific primer pairs or *ferredoxin-NADP*^*+*^ *reductase* (*FNR1*) as an internal control using SYBR PremixEx Taq (TaKaRa). The PCR reactions were run using Rotor-Gene Q (QIAGEN) and the program was set to 94 °C for 5 min, 40 cycles at 94 °C for 30 s, 53 °C for 30 s, and 72 °C for 30 s. The expression of *APX* and *Prx* target gene was calculated by subtracting the Ct value of the target from that of the internal control Ct to obtain ΔCt. The change in target gene expression was calculated by subtracting the ΔCt value of salt treatment from the ΔCt value of control treatment to obtained − ΔΔCt (ΔCt_control_ - ΔCt_treatment_), and the relative expression was displayed as 2^−ΔΔCt^. The expression of each gene was an average from 3 independent experiments, 5 replicates for each experiment.

### RNA-seq, *de novo* transcriptome assembly and differential expression analysis

The procedures for RNA-seq were conducted as described by Chiang et al. (2016). Samples were obtained from 200 mg of 3-day-old etiolated ice plant seedlings that were treated with either 0 or 200 mM NaCl for 6 h. Two libraries were constructed and 100 bp paired-end sequencing was performed by YourGene Bioscience (Taiwan) using Illumina^®^ Hiseq 2000 sequencing platform. Trimmomatic (Bolger et al. 2014) was employed to remove low-quality reads with a Phred quality score Q (Ewing and Green 1998) below 20 and to trim Illumina^®^ adaptor sequences, completing the preprocessing of reads prior to assembly. Although Shen et al. (2022) have published the first draft genome of the ice plant, they provide only raw sequencing data and lack accessible annotated gene sequences. Due to the absence of publicly available high-quality reference sequences and gene annotations for the ice plant, we performed *de novo* transcriptome assembly using Trinity (Grabherr et al. 2011) with default settings and a minimum contig length of 200 bases. In this process, Trinity first combines reads of a certain length that overlap by a specified amount to form longer sequences called contigs. Then, these contigs are connected to reconstruct their original transcriptional context, forming what are known as de Bruijn graphs. During assembly, Trinity automatically adjusts k-mer sizes and uses in silico read normalization to enhance assembly quality while managing computational resources efficiently. Transcriptome expression levels were quantified in terms of Transcripts Per Million (TPM) using eXpress (Roberts and Pachter 2013), which also estimates the number of reads mapping to each unigene for further differential gene expression analysis. Differential gene expression analysis was subsequently carried out using edgeR (McCarthy et al. 2012) with the dispersion parameter set to 0.01 and employing the trimmed mean of M-values (TMM) method (Robinson and Oshlack 2010). Unigenes displaying an absolute log2-fold-change (|log2FC|) greater than 2 and a false discovery rate (FDR) below 0.001 were identified as significantly differentially expressed genes (DEGs).

### Functional annotation and enrichment analysis

Functional annotation of assembled transcripts was conducted using the Trinotate suite (Bryant et al. 2017). The process commenced with the prediction of coding regions in transcripts using TransDecoder (http://transdecoder.github.io), specifically targeting sequences of at least 100 amino acids in length. Homology searches were then performed: both the assembled transcripts and the predicted protein sequences were searched against UniProtKB/Swiss-Prot (UniProt, 2023) using BlastX and BlastP (Altschul et al. 1990), respectively, with an E-value cutoff set at 10^− 5^. Conserved protein domains were identified through domain and profile homology searches using HMMER (Finn et al. 2011) against the Pfam (Mistry et al. 2021) database. Additionally, SignalP (Petersen et al. 2011) was used for predicting signal peptides, and Tmhmm (Krogh et al. 2001) for identifying transmembrane domains. The results were integrated into a Trinotate SQLite database that also includes data from UniProtKB/Swiss-Prot, eggNOG (Huerta-Cepas, 2019), Gene Ontology (GO) (Gene Ontology, 2008), Pfam-A (Mistry et al. 2021), and pfam2go (http://geneontology.org/external2go/pfam2go) databases. This integrated database facilitates the retrieval of Gene Ontology (GO) IDs and Kyoto Encyclopedia of Genes and Genomes (KEGG) IDs for proteins in UniProtKB/Swiss-Prot, thus providing insights into the corresponding GO terms and KEGG pathways for the assembled transcripts matching these proteins. Enrichment analysis for GO terms was conducted using Fisher’s exact test as the statistical model.

### Statistical analysis

The F-test and Student’s t-test in Excel were used to determine if there were significant differences between control and salt treatment. First, the F-test was employed to compare the variances of the two data sets (an F-test value ≥ 0.05 indicates that the two data sets have equal variances, while an F-test value < 0.05 indicates that the variances are not equal). Following this, Student’s t-test was used to assess whether there were significant differences between the two data sets, with the tails set to a two-tailed distribution. The type was determined based on whether the F-test indicated equal variances. A Student’s t-test value ≥ 0.05 indicates no significant difference between the two data sets, while a value < 0.05 indicates a significant difference.

## Results

### Growth inhibition due to salt treatment in ice plant seedlings

The ice plant has been recognized for its moderate salt tolerance at the seedling stage. Monitoring the growth of light-grown ice plant seedlings subjected to a salt treatment revealed rapid root growth upon the addition of fresh liquid MS medium (control) or MS medium supplemented with 200 mM NaCl (salt). No significant difference in root length was observed between control and salt treatment plants after 48 h (Supplementary Fig. 1A). In etiolated seedlings, the root lengths remained unchanged during the 48-h treatment, whereas hypocotyl lengths decreased after 24 h in response to the salt treatment (Supplementary Fig. 1B). These findings indicate that roots continued to elongate normally under the salt treatment, but the expansion of the hypocotyl was hindered.

### Accumulation of superoxide anion and hydrogen peroxide in ice plant seedlings

**Fig. 1:**
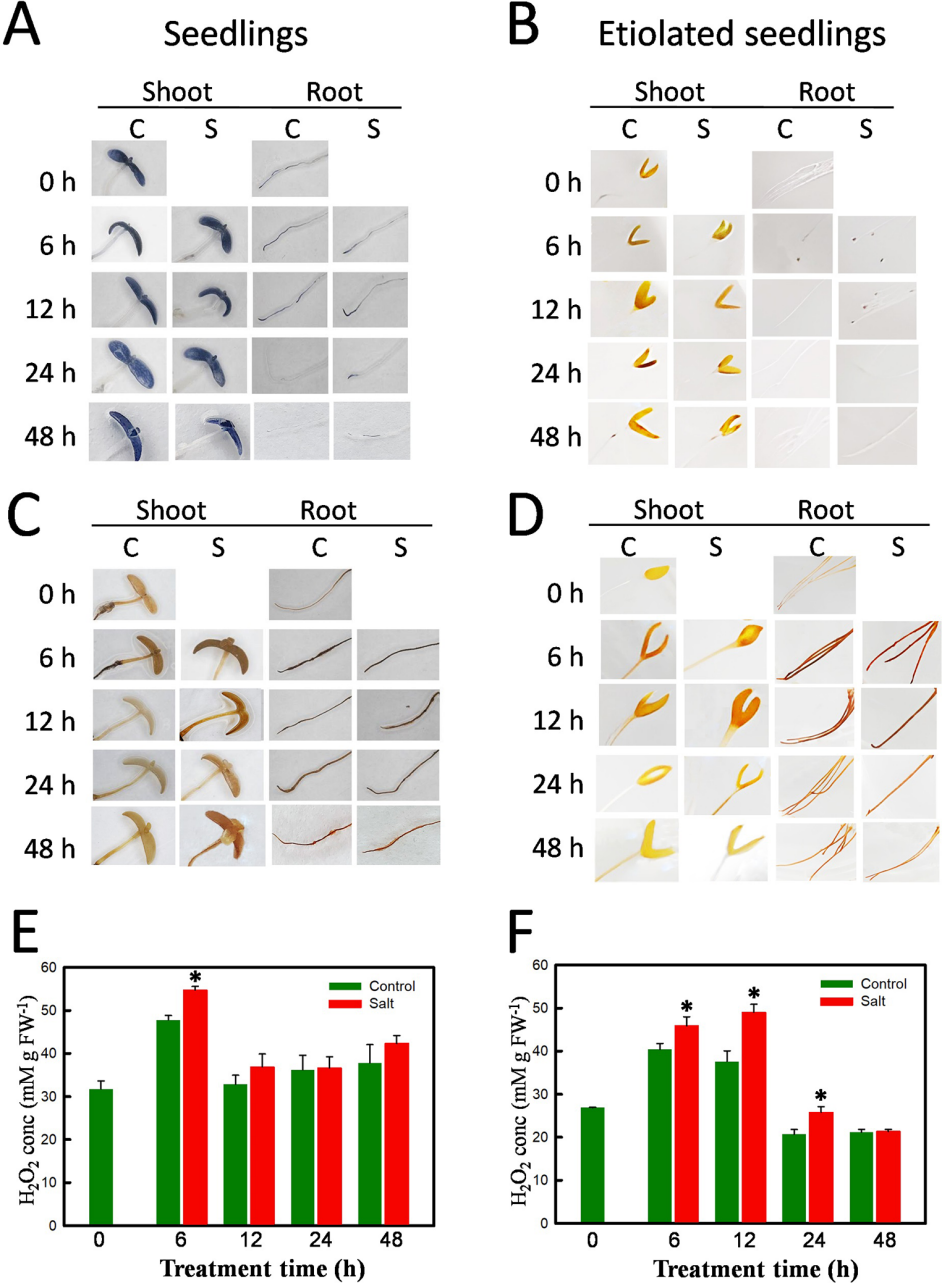
The accumulation of superoxide anion and hydrogen peroxide in ice plant seedlings. Five-d-old seedlings (Left panel) and 3-d-old etiolated seedlings (Right panel) were treated with MS liquid medium (Control; C) or 200 mM NaCl (Salt; S) for 0, 6, 12, 24–48 h. Seedlings were stained by NBT (A, B) for superoxide anion to produce blue coloration or stained by DAB (C, D) for hydrogen peroxide to produce brown coloration. The DAB-stained seedlings were quantified for H_2_O_2_ contents (E, F). Error bars represent mean ± SD (*n* = 3). All data were compared to control and analyzed by Student’s t-test (*p < 0.05)

Ice plant seedlings were treated with control (C) or salt (S) for 6, 12, 24, and 48 h and stained with NBT and DAB to detect superoxide anion and hydrogen peroxide, respectively (Fig. 1). Both control and salt-treated light-grown seedlings exhibited strong blue coloration in the cotyledons and primary leaves throughout the treatment period, with no discernible difference in staining intensity (Fig. 1A). Superoxide anion accumulation was detected in the root vascular tissues at the onset of both treatments, and this accumulation decreased from 24 h onward. However, only salt-treated roots retained a slight coloration after 48 h of treatment (Fig. 1A). In etiolated seedlings stained with NBT, the mixture of blue and yellow reflectance resulted in a green to brown coloration in the cotyledons, while no NBT staining was detected in the roots (Fig. 1B). Similar levels of NBT staining were observed in the cotyledons of both control and salt-treated seedlings. These findings indicate that the photosynthetic tissues of ice plant seedlings are the primary sites of superoxide anion production and that salt treatment does not affect superoxide anion accumulation.

H_2_O_2_ production was visualized in seedlings infiltrated with DAB, which reacts with H_2_O_2_ in the presence of peroxidase, forming a brown coloration product. A low level of H_2_O_2_ was observed in the cotyledons of ice plant seedlings at time 0. Upon the addition of the nutrient solution, a transient surge in H_2_O_2_ occurred at 6 h, indicated by the appearance of a strong brown coloration in the cotyledons and roots. Compared to control seedlings, H_2_O_2_ levels were notably higher in salt-treated seedlings and persisted for the entire 48-h treatment duration, while they were attenuated after 6 h in the control seedlings (Fig. 1C). In etiolated seedlings, a progressive increase in DAB staining was observed, peaking at 12 h and declining after 24 h of treatment in both cotyledons and roots. Strong brown coloration was evident in all parts of the roots for both control and salt treatments (Fig. 1D).

Cellular H₂O₂ levels are influenced by multiple reactions, with SOD-catalyzed conversion of superoxide anions being a major source, and are further regulated by oxidases and scavenging enzymes. We quantified the H_2_O_2_ levels in whole seedlings, and salt-induced H_2_O_2_ accumulation was observed in both light-grown (Fig. 1E) and dark-grown (Fig. 1F) seedlings with H_2_O_2_ levels increasing at 6 h and dropping after 24 h. This indicates that the salt treatment induced a transient ROS accumulation, with increased levels lasting for a longer duration in the etiolated seedlings. Additionally, there was a transient increase in H_2_O_2_ concentrations at 6 h in the control seedlings, suggesting that rapid root growth led to H_2_O_2_ accumulation upon the addition of the fresh nutrient solution.

### Antioxidant enzyme activity in ice plant seedlings under salt treatment

**Fig. 2:**
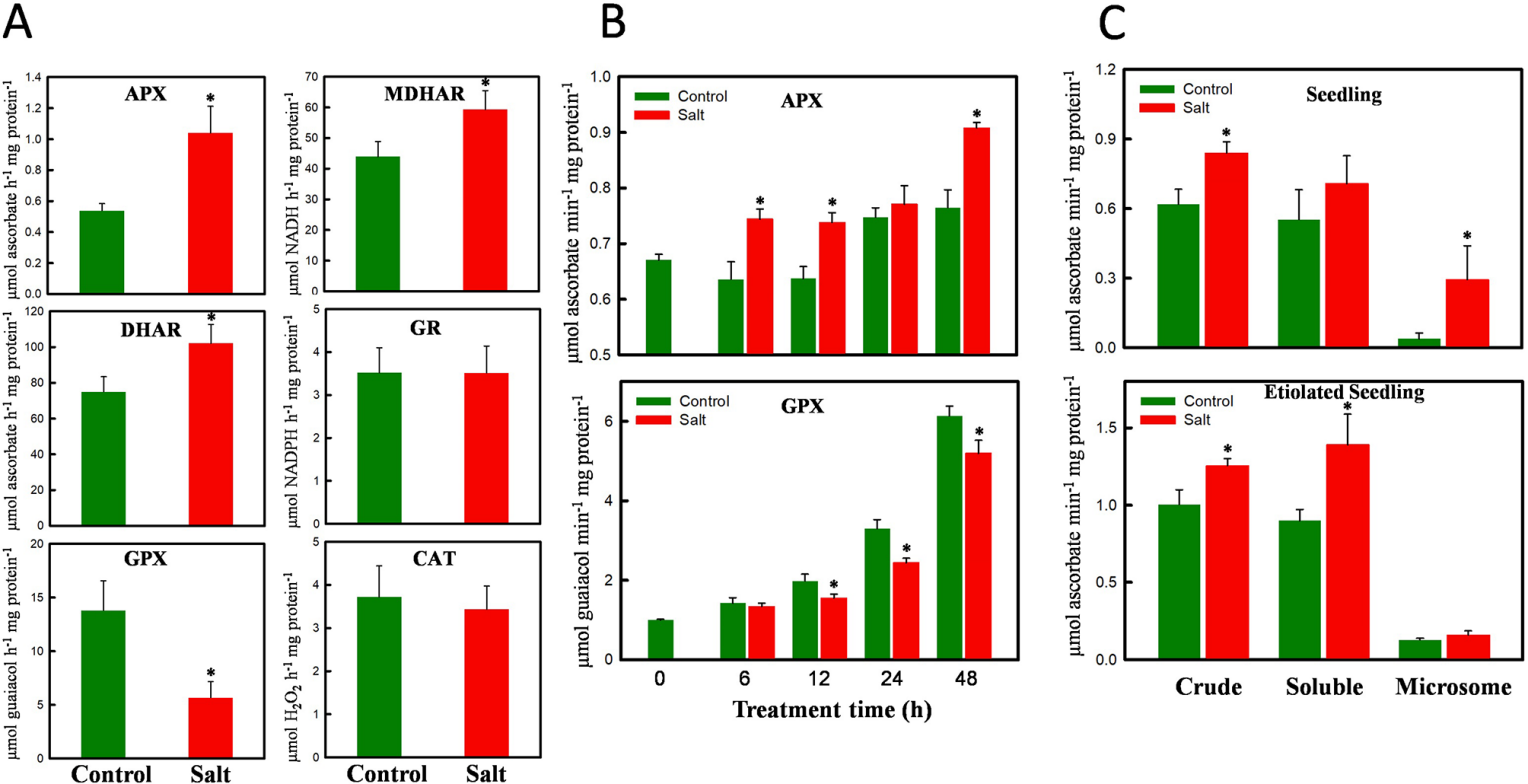
Activities of antioxidant enzymes in ice plant seedlings under salt treatment. (**A**) Activities of ascorbate-glutathione cycle enzymes ascorbate peroxidase (APX), monodehydroascorbate reductase (MDHAR), dehydroascorbate reductase (DHAR), and glutathione reductase (GR) and H_2_O_2_ reducing enzymes guaiacol peroxidase (GPX) and catalase (CAT) in 5-d-old ice plant seedlings treated with 0 mM (control) or 200 mM NaCl (salt) for 48 h. (**B**) Time-course activities of APX and GPX in 3-d-old etiolated ice plant seedlings treated with 0 mM (control) or 200 mM NaCl (salt). (**C**) Cellular fractionation of APX activities of ice plant seedlings treated with 0 mM (control) or 200 mM NaCl (salt) for 48 h. Crude extract (Crude) was separated into soluble fraction (Soluble) and microsomal fraction (Microsome). Error bars represent mean ± SD (*n* = 3). All data were compared to control and analyzed by Student’s t-test (*p < 0.05)

The findings depicted in Fig. 1 clearly illustrate that ice plant seedlings accumulated superoxide anions in their photosynthetic tissues, irrespective of whether they were subjected to the salt treatment. We performed SOD activity staining in native PAGE, and the results showed that all three SOD isoforms, MnSOD, FeSOD, and Cu/ZnSOD were constitutively active in ice plant seedlings, indicating high endogenous SOD activity (Supplementary Fig. 2A). There was no notable difference in SOD activity under the salt treatment except for a higher Cu/ZnSOD activity after 48 h of treatment. In contrast to the continuous accumulation of superoxide anions, ice plant seedlings exhibited a transient increase in H_2_O_2_ levels under both control and salt treatments. We measured the enzyme activities of H_2_O_2_ scavengers—including ascorbate-glutathione cycle enzymes APX, MDHAR, DHAR, and GR, as well as two enzymes involved in H_2_O_2_ reduction, GPX and CAT—in seedlings treated with 0 or 200 mM NaCl for 48 h. The results showed increased activities of APX, MDHAR, and DHAR and a significant decrease in GPX activity in the salt-treated seedlings, with no differences between control and salt-treated seedlings in GR and CAT activity (Fig. 2A).

Given that changes in H_2_O_2_ levels were also observed in etiolated seedlings, we measured the time-course activities of APX and GPX. During the 48-h treatment period, APX activity continuously increased in both the control and salt-treated groups, with the increase in the salt-treated seedlings being higher than that in the control group. GPX activity also continuously increased in both the control and salt-treated groups, but the increase in the salt-treated seedlings was less significant than in the control group (Fig. 2B). The results showed ice plant seedlings exhibited a different temporal pattern between APX and GPX, with salt-induced APX activity beginning 6 h after the initiation of salt stress, while GPX activity decreased after 12 h of salt treatment (Fig. 2B). These analyses of antioxidant enzyme activities indicate that ice plant seedlings possess intrinsic SODs, as well as activities associated with the ascorbate-glutathione cycle, guaiacol-dependent peroxidase, and catalase. Notably, the most prominent induction in response to the salt treatment among the examined antioxidant enzymes was observed in APX activity.

Different isoforms of APX are distributed across various compartments or organelles within higher plants. Through activity staining of APX via native PAGE, our results revealed a distinctive band around 38 kD, representing cytosolic APX, and a smear of multiple bands ranging from 50 to 100 kD, presumably indicating organelle- or membrane-bound APXs (Supplementary Fig. 2B). We separated the crude extract into soluble and microsomal fractions using ultracentrifugation and measured the APX activity in both fractions. Notably, in light-grown seedlings, salt-induced APX activities were detected in the microsomal fraction, which comprises chloroplastic and peroxisomal APXs. In etiolated seedlings, the salt-induced APX activities were primarily attributed to cytosolic APXs present in the soluble fraction (Fig. 2C). The major difference between light- and dark-grown seedlings lies in the development of chloroplasts. The chloroplast emerges as the primary site of ROS accumulation under salt stress in ice plant seedlings grown in the light, whereas in the absence of chloroplastic ROS production, the cytoplasm becomes the predominant site of ROS generation.

### Differentially expressed genes in ice plant seedlings under salt treatment

At the early developmental stage in ice plant, the salt treatment increased APX activity while decreasing GPX activity, and the enzyme assay accounted for the overall contributions of various isozymes distributed in different cellular compartments. There are 8 *APX* genes and 73 class III peroxidase genes identified in Arabidopsis. To identify the DEGs among *APX* and class III peroxidase, an RNA-seq analysis was performed on 3-day-old etiolated seedlings treated with 0 or 200 mM of NaCl for 6 h, a time point at which H_2_O_2_ accumulation is induced by salt treatment. The RNA-seq reads were *de novo* assembled using Trinity since ice plants lack an accessible reference genome. Detailed steps for the transcriptome assembly and DEG analysis are outlined in the Materials and Methods. The ice plant transcriptome assembly produced 85,667 transcripts, which were subsequently merged into 75,194 unigenes. Among these, 6,326 unigenes exhibited more than a fourfold change in expression levels (False Discovery Rate, FDR < 0.001) in response to the salt treatment. Thus, approximately 8% of the unigenes are significantly affected by salt treatment in ice plant seedlings. Plotting the expression levels against the FDR identified 3,917 unigenes with increased expression and 2,409 with decreased expression (Supplementary Fig. 3).

**Fig. 3:**
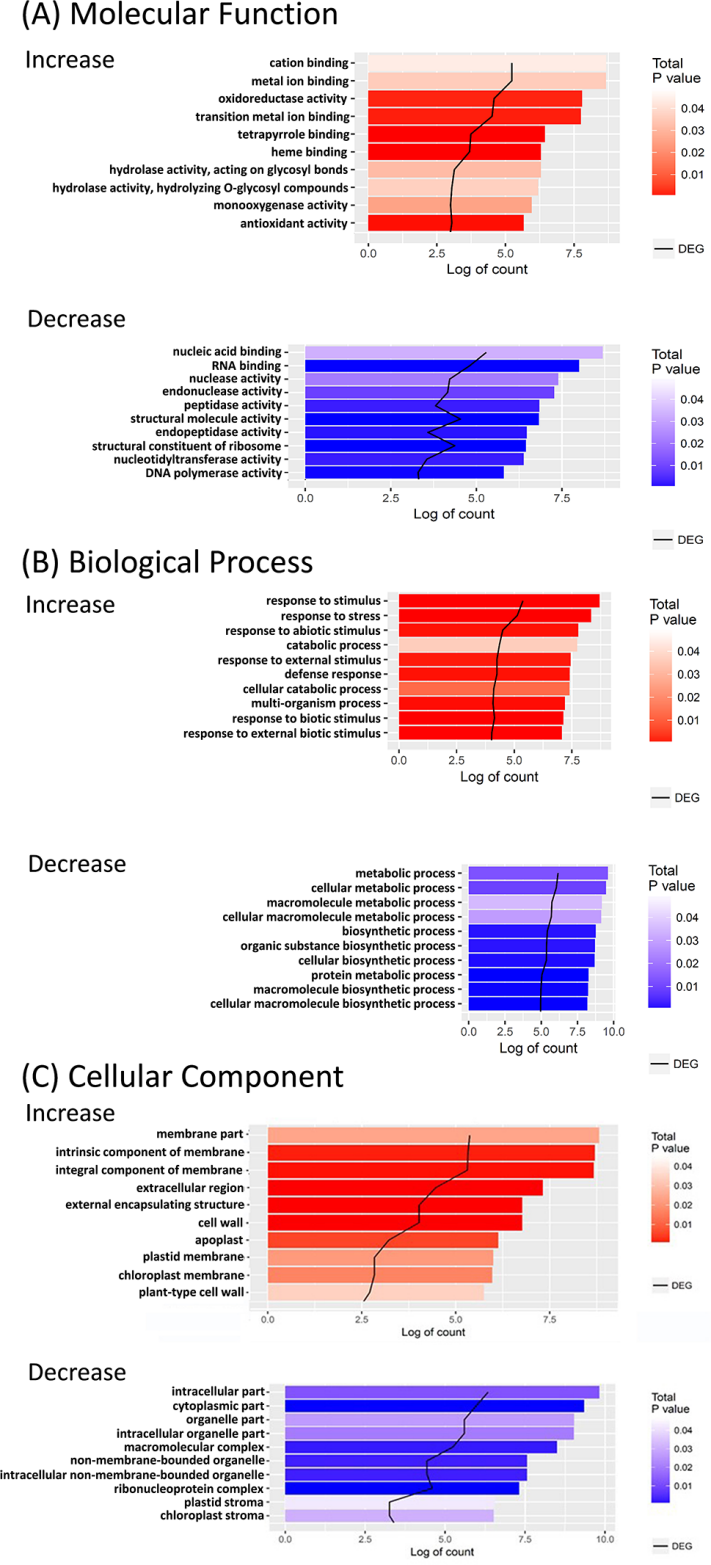
Top 10 GO terms of 4-fold increase and decrease DEGs in ice plant seedlings treated with 200 mM NaCl for 6 h. The GO terms are classified by (**A**) molecular function, (**B**) biological process and (**C**) cellular component. The color bars show the values of statistical significance (P value < 0.05). Red color indicates increased and blue color indicates decreased transcript abundance. The black lines indicate numbers of DEGs

To annotate the DEGs identified in ice plant seedlings under salt stress, we aligned the assembled transcriptome to sequences in various databases. A substantial portion of the unigenes (41,625; 55.35%) aligned with an ice plant cDNA library established by Dr. Cushman containing 24,204 full-length or nearly full-length cDNA sequences. Moreover, approximately 43% of DEGs (2,744 unigenes) were identified in Cushman’s database (Table 1). Alignment against the Swiss-Prot database led to annotations for 28,520 unigenes (37.93%), with 27,843 unigenes (37.03%) annotated with GO terms, and 4,754 unigenes (6.32%) annotated with KEGG pathways (Table 1). The top 10 GO terms for 4-fold increase or decrease in the Molecular Function, Biological Process, and Cellular Component categories were graphed (Fig. 3). In the Molecular Function category, half of the top 10 increased terms were linked to “cation binding,” “metal ion binding,” “oxidoreductase activity,” “monooxygenase activity,” and “antioxidant activity.” The top three increased GO terms in the Biological Process category were associated with “responses to stimuli,” “stress responses,” and “abiotic stimuli,” whereas the decreased terms were all linked to “metabolic process” and “biosynthetic process.” The top increased GO term in the Cellular Component category was associated with “membrane parts.” The enrichment analysis reveals that the increased terms are primarily involved in ion binding, redox activities, and stress responses, while the decreased terms are associated with metabolic and biosynthetic processes.

**Table 1:** Summary of unigene annotations of ice plant transcriptome using different databases

Annotated databases	Transcriptome	4FC* DEG**	2FC DEG
Unigene number	75,194	6,326	8,585
Cushman	41,625 (55.35%)	2,744 (43.37%)	4,485 (52.24%)
Swiss-Prot	28,520 (37.93%)	1,564 (24.72%)	3,079 (35.86%)
GO	27,843 (37.03%)	1,516 (23.96%)	2,811 (32.74%)
KEGG	4,754 (6.32%)	809 (12.79%)	1,549 (18.04%)

*FC: fold change

**DEG: differentially expressed gene

**Fig. 4:**
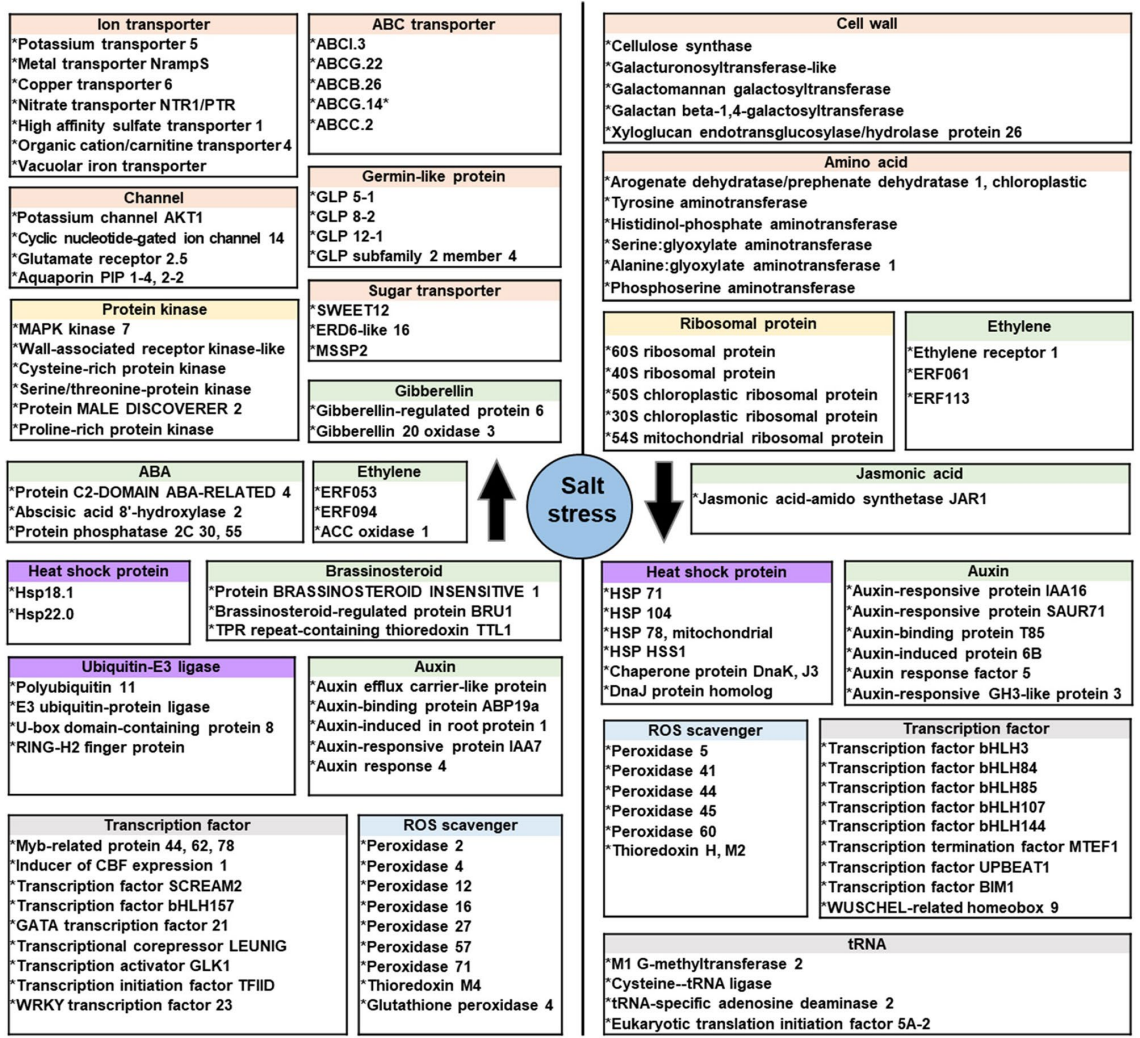
Summarization the function of differentially expressed genes (DEGs) in ice plant seedlings under salt stress. Three-day-old dark-grown ice plant seedlings were treated with 0 mM or 200 mM NaCl for 6 h. Unigenes grouped by functional category exhibit a significant increase in expression are shown in the left panel, and exhibit a significant decrease in expression are shown in the right panel

The GO-annotated unigenes exhibiting significant changes in expression were grouped (Supplementary Table 1) and are summarized in Fig. 4. The initial responses of ice plant seedlings to salt treatment involve both unique pathways and pathways common to both salt-treated and control plants. Ion transporter, channel, and germin family member genes were uniquely increased (left panel, Fig. 4), whereas specific genes encoding enzymes involved in cell wall and amino acid synthesis were decreased following salt exposure (right panel, Fig. 4). For common pathways, members of the class III peroxidase and heat shock protein families were differentially expressed, with some genes increased and others decreased. Genes associated with ABA signaling were uniquely increased, whereas those involved in auxin and ethylene signaling showed differential expression. The results indicate that genes related to growth, including those associated with ribosomal proteins, amino acid metabolism, and cell wall synthesis, were significantly decreased by the salt treatment, suggesting that salt inhibits the growth of ice plant seedlings. Concurrently, genes involved in ion transport were increased to facilitate the compartmentation of toxic ions and maintain cellular ion homeostasis.

### Salt-induced changes in the expression of all *APX* and selected *Prx* family members

**Fig. 5:**
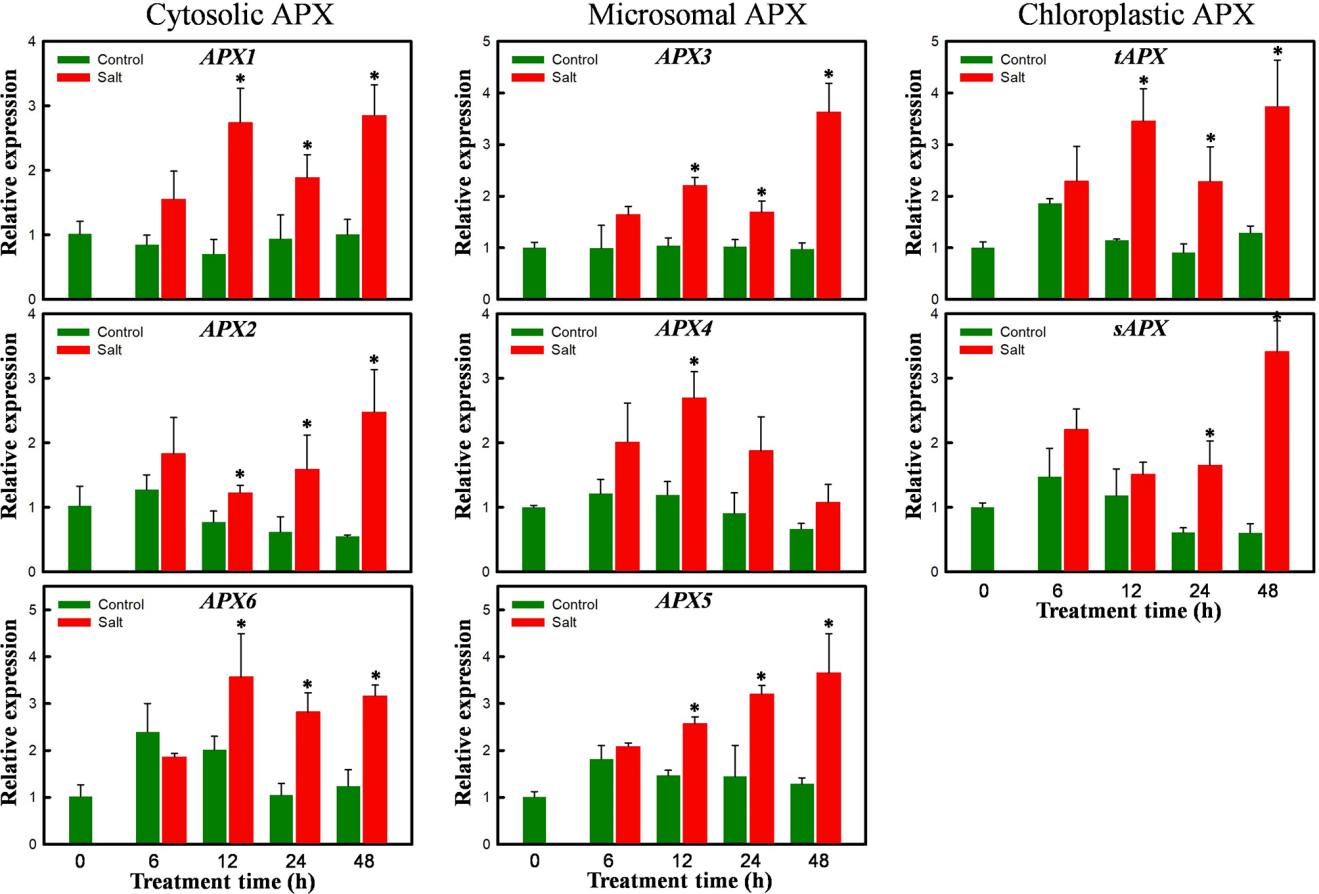
Temporal expression of *APX* family in 3-d-old etiolated ice plant seedlings treated with 0 (Control) or 200 mM NaCl (Salt) for 0, 6, 12, 24 and 48 h. APX gene family includes three cytosolic APXs (*APX1*, *2*, and *6*), three peroxisomal (microsomal) APXs (*APX3*, *4* and *5*), and two chloroplastic APXs (stromal *sAPX* and thylakoid *tAPX*). The expression of *McFNR1* was used as an internal control. The expression of time 0 treatment of each gene was set as 1. Average values of qRT-PCR were from 5 samples of each treatment. Error bars were calculated using Excel STDEVP function. Asterisks indicate *P* < 0.05 comparing Control and Salt treatment based on pairwise Student’s *t* test

By searching the ice plant transcriptome, we identified at least 19 assembled transcripts that were orthologous to Arabidopsis APX family genes, including those encoding three cytosolic APXs (*APX1*, *2*, and *6*), three peroxisomal APXs (*APX3*, *4* and *5*), and two chloroplastic APXs (*sAPX* and *tAPX*). Though none of these transcripts met the stringent criteria, |Fold Change (FC)| > 4 and FDR < 0.001, to qualify as DEGs in the GO annotation profiles, their expression was examined in detail. Based on the transcriptome sequence information, primer pairs were designed to amplify all eight ice plant *APX* members, and each primer pair amplified a single band using conventional RT-PCR (data not shown). Quantitative RT-PCR was used to examine the temporal expression patterns of the *McAPX*s during 48 h of salt treatment. The results showed the expression of all eight *McAPX*s was induced by salt treatment (Fig. 5). Among them, the expression of seven *McAPX*s showed a progressive increase over the 48-h treatment, whereas the expression of *APX4* exhibited a transient surge 12 h after the initiation of the salt treatment. At the initial 6-h time point, the expression levels of all eight *McAPX*s did not exhibit significant differences between the control and salt-treated samples. This lack of significant differences in expression at 6 h could potentially explain the failure to detect the *McAPX*s as DEGs in the transcriptome results, as the samples for transcriptome profiling were collected at the 6-h mark following salt treatment.

**Fig. 6:**
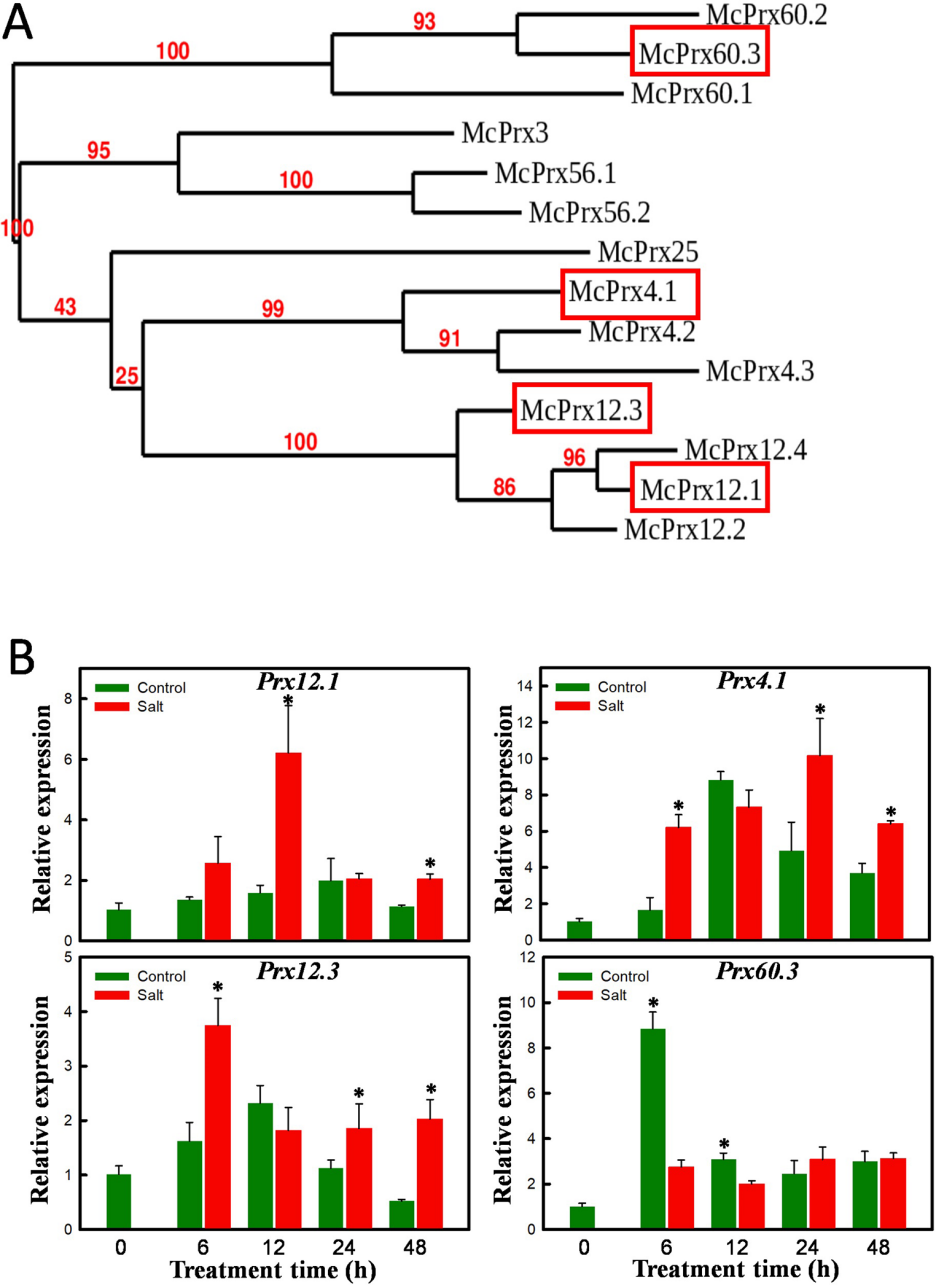
Temporal expression of selected *Prx* family members. (**A**) Phylogenetic analysis of 14 ice plant *Prx* genes with |FC| > 4 and FDR < 0.001. The branch numbers were branch support values. Four ice plant *Prx* members chosen from distinct external branches are marked in red boxes. (**B**) The total RNA was extracted from 3-day-old etiolated seedlings treated with 0 (Control) or 200 mM NaCl (Salt) for 0, 6, 12, 24 and 48 h. The expressions of *McPrx4.1*, *12.1*,*12.3* and *60.3* were detected by qRT-PCR. The expression of *McFNR1* was used as an internal control. The expression of time 0 treatment of each gene was set as 1. Average values of qRT-PCR were from 5 samples of each treatment. Error bars were calculated using Excel STDEVP function. Asterisks indicate *P* < 0.05 comparing Control and Salt treatment based on pairwise Student’s *t* test

There are 73 members of the class III peroxidase (*Prx*) family of genes in Arabidopsis. By searching the ice plant transcriptome, we identified at least 53 assembled transcripts that were annotated as *Prx* members (Supplementary Fig. 4). Among these, 14 unigenes met the stringent DEG criteria, |FC| > 4 and FDR < 0.001. These selected unigenes were renamed based on the Arabidopsis nomenclature, and their phylogenetic relationships are depicted in Fig. 6A. The deduced amino acid sequences of all 14 peroxidases contain eight highly conserved cysteines and three conserved domains. Four ice plant *Prx* members were chosen, each from distinct external branches, for expression analysis, and single bands were successfully amplified using conventional RT-PCR (data not shown). The quantitative RT-PCR results showed that the expression of *McPrx4.1*, *McPrx12.1*, and *McPrx12.3* was induced by salt exposure, while the expression of *McPrx60.3* was decreased, matching the DEG results (Fig. 6B). The expression profiles of salt-induced *Prxs*, namely *McPrx4.1*, *McPrx12.1*, and *McPrx12.3*, exhibited distinct patterns, with the highest expression levels observed 24, 12, and 6 h following salt treatment, respectively. These differing expression patterns strongly suggest that individual members of the class III peroxidase family play unique and specific roles in the trade-offs between plant growth and salt tolerance in ice plant seedlings.

## Discussion

### Hydrogen peroxide acts as an ROS and a growth-promoting molecule

Ice plant is a halophyte and an inducible CAM plant capable of thriving in high-salinity environments. This adaptability is attributed in part to its efficient antioxidant system, which mitigates oxidative stress induced by salinity. Our study demonstrates that ice plant seedlings possess active antioxidant systems and an inherent salt tolerance, even in the absence of functional photosynthesis and CAM. In dark-grown seedlings, the primary ROS is H_2_O_2_ (Fig. 1), which largely originates from the mitochondrial electron transport chain, in which superoxide radicals are produced and subsequently converted to H_2_O_2_ by MnSOD (Borland et al. 2006). The glyoxylate cycle in glyoxysomes also generates H_2_O_2_ in germinating seeds. Additionally, H_2_O_2_ also accumulates within the apoplast of rapidly expanding root cells. This H_2_O_2_ is derived from class III peroxidases and other cell wall enzymes, such as oxalate oxidase, also known as germin, which releases H_2_O_2_ and CO_2_ from oxalic acid (Voothuluru et al. 2020). We identified an increase in four germin-like transcripts in salt-stressed ice plant seedlings (Fig. 4), which may contribute to the increased H_2_O_2_ levels.

Upon the addition of fresh liquid MS medium, the root and hypocotyl continued to grow for 48 h (Supplementary Fig. 1), with H_2_O_2_ levels increasing at 6 h and dropping after 24 h in both control and salt-treated seedlings (Fig. 1). Konieczny et al. (2014) observed a similar transient increase in H_2_O_2_ accumulation during the early stages of root differentiation in the roots of ice plants cultured on a medium inducing root differentiation. The involvement of H_2_O_2_ in auxin-related responses during root development and differentiation has long been recognized (Ivanchenko et al. 2013). We believe that this initial high accumulation of H_2_O_2_ functions as a signaling molecule facilitating ice plant growth. Compared to the control seedlings, higher concentrations of H_2_O_2_ were observed in the salt-treated seedlings at 6, 12, and 24 h, with no significant difference at 48 h indicating that under salt stress, antioxidant mechanisms are activated, leading to a reduction in H_2_O_2_ concentrations. Correlating this reduction with the temporal analyses of *APX* expression (Fig. 5) and APX activity (Fig. 2), we propose that the ascorbate-glutathione cycle plays a major role in H_2_O_2_ scavenging under salt stress in ice plant seedlings.

In addition to its role as an ROS and growth-promoting molecule, H_2_O_2_ may act as a signaling molecule in the induction of the metabolic shift from C3 to CAM in ice plants, thereby alleviating water-deficit stress (Ślesak et al. 2007). Concerns regarding O_2_ concentrations in CAM plants have been longstanding. Internal O_2_ levels can rise to 42% during the daytime when the photosynthetic electron transport chain is active and stomata are closed, increasing the potential for oxidative stress (Lüttge 2002). On the other hand, when daytime malate decarboxylation is operating, the enriched internal CO_2_ prevents excessive energy accumulation through the photosynthetic electron transport chain, thus protecting against photoinhibition (Adams and Osmond 1988). However, once the malate stored in the vacuole is depleted, a high O_2_ partial pressure may develop before stomates reopen in phase IV, presenting a high potential for ROS generation in the late afternoon. To address this issue, Borland et al. (2006) compared SOD activity in wild-type and CAM-deficient ice plant mutants. They found that salt-induced increases in the activities of three SOD isoforms were significantly higher in CAM-deficient mutants than in wild-type plants. Additionally, Sunagawa et al. (2010) demonstrated that the activities of CAT and APX, along with H_2_O_2_ levels, were also higher in CAM-deficient mutants compared to wild-type ice plants. These findings suggest that performing CAM lowers ROS levels, thereby alleviating the oxidative burden under environmental stress.

### Peroxidases attenuate hydrogen peroxide levels to achieve salt tolerance

Plants possess two types of heme-containing peroxidases: class I peroxidases, such as APX, which are widespread from prokaryotes to higher eukaryotes, and class III peroxidases, which are unique to plants. Class III peroxidases constitute a large gene family that plays crucial roles in growth and development, particularly in cell wall expansion and tightening. The activity of class III peroxidases significantly increases during root formation in grapevine (Vatulescu et al. 2004) and fruit development in tomato (Thomas et al. 1982). Cell wall-bound peroxidases catalyze reversible H_2_O_2_-producing or -scavenging reactions, which play roles in regulating the biosynthesis of cell wall components, including lignin (Ros Barceló 1998). The bifunctional nature of class III peroxidases, which depends on whether the reaction favors the consumption or generation of H_2_O_2_, allows them to regulate growth cessation through cell wall cross-linking (MacAdam et al. 1992b) or promote cell elongation in hypocotyls (Dunand et al. 2003). During the 48-h treatment period, a rapid elongation of hypocotyls in 3-day-old etiolated ice plant seedlings was accompanied by a transient increase in H_2_O_2_ concentrations (Fig. 1) and a continuous increase in peroxidase activity (Fig. 2), as measured using the oxidation of guaiacol, in both control and salt-treated seedlings. Salt-treated seedlings exhibited higher H_2_O_2_ levels and lower GPX activity. The peak in H_2_O_2_ accumulation observed at 12 h (Fig. 1F) was likely due to decreased GPX activity (Fig. 2B), whereas the subsequent decline in H_2_O_2_ levels after 48 h of salt stress was likely attributed to increased APX activity. The expression level of *McPrx60.3* (Fig. 6) was inversely correlated with H_2_O_2_ levels, suggesting that *McPrx60.3* is involved in promoting hypocotyl elongation in etiolated seedlings. In contrast, decreased *McPrx60.3* expression, leading to lower GPX activity, slows cell elongation under salt stress.

The diverse functions of class III peroxidases are evidenced by the distinct expression patterns among its family members. The expression levels of *McPrx12.3*, *McPrx12.1*, and *McPrx4.1* all increased under the salt treatment, with peak levels 6, 12, and 24 h, respectively, after salt treatment began. Kim et al. (2008) showed that transgenic tobacco plants expressing sweet potato (*Ipomoea batatas*) *Prx4* exhibited reduced H_2_O_2_ levels accompanied by an enhanced tolerance to abiotic stress. Similarly, overexpression of *AtPrx12* in Arabidopsis reduced ROS accumulation under cold stress, thereby increasing cold tolerance (Kim et al. 2012). The different temporal expression patterns of these three *Prx* members suggest that they play roles in different stages of the salt stress response rather than in promoting plant growth.

The levels of H_2_O_2_ induced by oxidative stress in different cellular compartments are regulated by specific peroxidase systems. Class III peroxidases are the sole H_2_O_2_-scavenging enzymes within the apoplastic and vacuolar spaces, while APX isoenzymes are distributed in the cytosol, chloroplasts, mitochondria, and peroxisomes/glyoxysomes (Dumanović et al. 2021). Nosek et al. (2018) showed that treating 6-week-old ice plants with 400 mM NaCl for 14 days resulted in higher APX activity compared to the control treatment. Consistent with this finding, Fig. 2 shows that while overall GPX activity decreased, overall APX activity increased, indicating that APX is the major H_2_O_2_ scavenger in salt-stressed seedlings. Although APX activity increased under salt treatment in both light- and dark-grown seedlings, the major contributors to the increased enzyme activity differed: APX activity increased in the microsomal fraction in light-grown seedlings, while it increased in the cytosolic fraction in dark-grown seedlings (Fig. 2C). Thus, ROS generated from chloroplasts in light-grown seedlings were actively scavenged via the ascorbate-glutathione cycle to mitigate oxidative damage. In etiolated seedlings, cytoplasmic H_2_O_2_ levels were regulated by cytosolic APXs under salt stress, as demonstrated by the increased expression of the cytosolic APX genes *McAPX1*, *McAPX2*, and *McAPX6* (Fig. 5). Wang et al. (2005) showed that transgenic tomato plants expressing tobacco cytosolic *APX* exhibited increased APX enzyme activity, reduced ion leakage rates, and decreased oxidative damage under low-temperature and salt stress, highlighting the critical role of cytosolic APX in the salt stress response.

Although the seedlings were maintained in darkness, the expression of peroxisomal membrane-bound *McAPX3*, *McAPX4*, and *McAPX5*, as well as chloroplastic *MctAPX* and *McsAPX*, increased upon salt treatment (Fig. 5). Wang et al. (1999) found that transgenic tobacco plants expressing Arabidopsis *APX3* had reduced malondialdehyde contents and ion leakage rates, enhancing tolerance to oxidative stress. The expression patterns of *McAPX3* and *McAPX5* showed a continuous increase for 48 h under salt stress, while *McAPX4* expression peaked at 12 h before declining, suggesting that *McAPX4* primarily functions in early peroxisomal-associated antioxidant mechanisms during salt stress, with *McAPX3* and *McAPX5* taking over in later stages. Furthermore, this study detected the expression of *tAPX* and *sAPX* genes in etiolated seedlings, with their expression levels increasing under salt stress. This indicates that *tAPX* and *sAPX* are indeed transcribed in seedlings grown in the dark. Since etiolated seedlings lack fully functional chloroplasts, whether *tAPX* and *sAPX* are translated into proteins requires further investigation.

### Transcriptome analysis provides a gene mining reference for improving plant salt tolerance

In this study, an active antioxidant system was observed in ice plant seedlings without the complication of CAM induction. Our DEG analysis of seedling transcriptomes identified 6,326 out of 75,194 unigenes that were differentially expressed under a 200 mM salt treatment using an FDR < 0.001 and |FC| > 4 cutoff. Genes with increased transcript abundance (3,917 unigenes) were primarily associated with ion transport and stress responses, whereas genes with decreased transcript abundance (2,409 unigenes) were linked to growth-related processes, such as ribosomal protein synthesis and cell wall formation (Fig. 4). A list of annotated DEG is provided in Supplementary Table 1, which highlights the complex and multifaceted response of ice plant to salt stress at the seedling stage. This response involves osmoprotection, ion homeostasis, antioxidant defense, signal transduction, and transcriptional regulation. For example, Kawase et al. (2013) introduced the ice plant aquaporin gene *McMIPB*, encoding a PIP1-type aquaporin found in the plasma membrane of xylem parenchyma, into tobacco, which improved photosynthetic responses and growth under water-deficit conditions. In the Channel category of Fig. 4, aquaporin PIP 2–2 is listed alongside PIP 1–4, a PIP1-type aquaporin, suggesting PIP 2–2 as another potential candidate for exploring water channel functions in salt tolerance. Additionally, the potassium channel AKT1 in Fig. 4 highlights the role of Na/K homeostasis. Nishijima et al. (2017) identified the gene *McHKT2*, which encodes a high-affinity potassium transporter orthologous to the Arabidopsis sodium transporter *AtHKT1*, from an ice plant transcriptome dataset. They observed that overexpression of *McHKT2* in Arabidopsis significantly enhanced salt tolerance, demonstrating the potential role of this transporter in maintaining ion homeostasis under saline conditions. Understanding these mechanisms in halophytes offers valuable insights for improving crop salt tolerance.

Tsukagoshi et al. (2015) *de novo* assembled 53,516 contigs from ice plant seedling roots. Of these, 10,818 contigs had orthologs in the Arabidopsis genome. They identified 152 up-regulated and 42 down-regulated genes (cutoff: FDR < 0.05 and |FC| > 2) in response to 250 mM NaCl exposure. Comparing salt-induced gene expression between Arabidopsis and ice plant, they found ABA-responsive genes to be up-regulated and the sodium transporter HKT1 to be down-regulated in both species. However, three peroxidase genes were up-regulated in Arabidopsis and down-regulated in ice plant. The number of salt-regulated unigenes reported by Tsukagoshi et al. (2015) was much lower compared to our results. Nevertheless, they showed that the differential expression of the peroxidase family genes results in different degrees of salt tolerance in ice plant and Arabidopsis, which was further confirmed in this study.

Previously, we analyzed the small RNA profile of ice plant seedlings and identified many conserved miRNA families and several novel miRNAs. The functions of predicted target genes were mostly transcription factors regulating growth and development for most conserved miRNAs and transporters involved in ion homeostasis for some novel miRNAs (Chiang et al. 2016). The target gene of mcr-miR3, an inositol transporter (INT), drew our attention. A 5-fold increase in *INT* expression accompanied the downregulation of mcr-miR3 in 3-day-old dark-grown seedlings salt-treated for 6 h. Subsequently, we found that an exogenous supply of *myo*-inositol mitigated salt-induced damage in ice plant seedlings and that the salt-induced *INT* expression was accompanied by a 2-fold increase in the inositol uptake rate of ice plant seedlings (Li et al. 2021). *Myo*-inositol is the precursor for the biosynthesis of pinitol, the major compatible solute accumulated in ice plant, which constitutes up to 71% of the soluble carbohydrates in the leaves of adult ice plants treated with 400 mM NaCl (Paul and Cockburn 1989). Free radical scavenging activities in the leaves of plants treated with 400 mM NaCl were two-fold higher than those of the control (Agarie et al. 2009), suggesting that the accumulation of polyols, including *myo*-inositol, ononitol, and pinitol, contributes to the increased antioxidative activity in salt-stressed ice plants (Li et al. 2023). In conjunction with the findings of the current study, the intrinsic responses to salt treatment in this halophyte include improved ion homeostasis and osmoprotection, along with a reduction in cell metabolism and expansion.

## Conclusion

We observed an active antioxidant system acting to protect cellular integrity in ice plant seedlings under salt stress. Transcriptome analysis showed that approximately 8% of identified unigenes were significantly affected by the salt treatment, with genes showing increased transcript abundance associated with ion transport and stress responses, and genes showing decreased transcript abundance linked to growth-related processes. The distinct expression profiles of *McPrxs* suggest that class III peroxidase members play a role in balancing plant growth and stress response. The activity of APX isozymes increased significantly in salt-treated seedlings, particularly in the microsomal fraction of light-grown and cytosolic fraction of dark-grown seedlings, highlighting their roles in mitigating oxidative damage. The enhanced expression of cytosolic and peroxisomal *APX* genes under salt stress supports the importance of these enzymes in the salt stress response, even in etiolated seedlings lacking fully functional chloroplasts. These findings provide a deeper understanding of the molecular responses in ice plant seedlings, paving the way for future research on stress adaptation in halophytes.

## Electronic supplementary material

Below is the link to the electronic supplementary material.

Supplementary Material 1

Supplementary Material 2

## Data Availability

RNA-seq data and list of DEGs were deposited to Gene Expression Omnibus (GEO) database in NCBI under accession number GSE277109.
