# Supplementary material for: Transcriptomic and enzymatic analysis of peroxidase families at the early growth stage of halophyte ice plant (Mesembryanthemum crystallinum L.) under salt stress
Source: Bot Stud. 2025 Jan 21;66:5. doi: 10.1186/s40529-024-00450-y (PMC11751343; doi:10.1186/s40529-024-00450-y)
Supplement: Supplementary file 1 — Supplementary Material 1 [file 40529_2024_450_MOESM1_ESM.pdf]

Supplementary Table 1. Annotation profile of DEG in ice plant seedlings treated with 200 mM NaCl (|FC|>4, FDR<0.001)

No.	Transcript ID	Length	Control reads	Control reads (TPM**)	Salt reads	Salt reads (TPM)	logFC*	Annotation
1	TRINITY_DN35173_c2_g4	388	0.00	0.00	901.31	94.65	12.80	Ribulose bisphosphate carboxylase small chain 2, chloroplastic
2	TRINITY_DN26866_c0_g1	722	0.17	0.01	411.81	23.24	11.67	U-box domain-containing protein 38
3	TRINITY_DN531_c0_g1	830	0.00	0.00	346.80	17.03	11.42	Mitogen-activated protein kinase kinase 7
4	TRINITY_DN32070_c0_g1	1484	0.19	0.01	315.31	8.66	11.28	DNA topoisomerase 6 subunit B
5	TRINITY_DN34794_c0_g2	883	0.11	0.01	289.13	13.34	11.16	Exocyst complex component EXO70B1
6	TRINITY_DN30102_c1_g1	1047	0.34	0.01	271.18	10.55	11.06	Mitochondrial import receptor subunit TOM20
7	TRINITY_DN31296_c0_g1	982	0.00	0.00	231.88	9.62	10.84	Transcription factor bHLH157
8	TRINITY_DN28467_c0_g1	483	0.00	0.00	229.63	19.37	10.83	L-ascorbate oxidase
9	TRINITY_DN30244_c0_g1	1009	0.04	0.00	194.00	7.83	10.58	E3 ubiquitin-protein ligase RHF1A
10	TRINITY_DN12933_c0_g2	1008	0.00	0.00	181.32	7.33	10.48	Zinc finger CCCH domain-containing protein 48
11	TRINITY_DN26089_c0_g2	923	0.00	0.00	177.10	7.82	10.45	Protein NRT1/ PTR FAMILY 5.8
12	TRINITY_DN13191_c0_g2	1449	1.33	0.04	1487.22	41.82	10.33	50S ribosomal protein L10, chloroplastic
13	TRINITY_DN3585_c0_g2	581	0.00	0.00	161.30	11.31	10.31	Bifunctional protein FolD 2
14	TRINITY_DN33589_c0_g9	711	0.37	0.02	153.86	8.82	10.25	Protein TIC 40, chloroplastic
15	TRINITY_DN33589_c0_g4	711	0.34	0.02	144.85	8.30	10.16	Protein TIC 40, chloroplastic
16	TRINITY_DN33589_c0_g5	711	0.30	0.02	135.51	7.77	10.07	Protein TIC 40, chloroplastic
17	TRINITY_DN22587_c0_g2	536	0.00	0.00	120.87	9.19	9.90	Reticulon-like protein B12
18	TRINITY_DN33888_c1_g2	447	0.00	0.00	106.50	9.71	9.71	Importin subunit alpha-9
19	TRINITY_DN34803_c0_g1	1361	0.00	0.00	93.53	2.80	9.54	Protein SUPPRESSOR OF PHYA-105 1
20	TRINITY_DN33423_c0_g3	550	0.00	0.00	85.85	6.36	9.41	Replication protein A 70 kDa DNA-binding subunit B
21	TRINITY_DN24429_c0_g1	557	0.00	0.00	85.29	6.24	9.39	Psoralen synthase
22	TRINITY_DN4826_c0_g2	1555	0.43	0.01	80.07	2.10	9.30	U-box domain-containing protein 8
23	TRINITY_DN35266_c1_g1	743	0.00	0.00	78.78	2.24	9.29	Cytochrome c oxidase subunit 1
24	TRINITY_DN23630_c0_g2	853	0.00	0.00	77.94	3.72	9.27	LIM domain-containing protein PLIM2c
25	TRINITY_DN30910_c0_g1	1455	0.00	0.00	75.32	2.11	9.21	Dof zinc finger protein DOF5.3

*logFC: the logarithm to base 2 of fold change (Salt/Control)

**TPM: transcripts per million

Supplementary Table 1. (Cont) Annotation profile of DEG in ice plant seedlings treated with 200 mM NaCl (|FC|>4, FDR<0.001)

No.	Transcript ID	Length	Control reads	Control reads (TPM**)	Salt reads	Salt reads (TPM)	logFC*	Annotation
26	TRINITY_DN24409_c1_g1	1252	0.00	0.00	75.05	2.44	9.21	LINE-1 retrotransposable element ORF2 protein
27	TRINITY_DN30229_c0_g2	2815	0.00	0.00	75.28	1.09	9.21	TPR repeat-containing thioredoxin TTL1
28	TRINITY_DN33246_c0_g2	2079	0.13	0.00	73.65	1.44	9.19	RING finger protein 141
29	TRINITY_DN24274_c0_g2	973	2.28	0.10	1222.03	51.18	9.13	Protein HHL1, chloroplastic
30	TRINITY_DN31037_c0_g1	473	0.00	0.00	69.69	6.00	9.11	Glycerol-3-phosphate acyltransferase 5
31	TRINITY_DN33589_c0_g8	716	0.15	0.01	65.67	3.74	9.03	Protein TIC 40, chloroplastic
32	TRINITY_DN14313_c0_g2	635	0.00	0.00	60.74	3.90	8.91	Stellacyanin
33	TRINITY_DN28719_c0_g1	1194	0.00	0.00	59.37	2.03	8.87	Receptor-like serine/threonine-protein kinase At1g78530
34	TRINITY_DN26365_c1_g1	239	0.00	0.00	55.89	9.53	8.79	3-ketoacyl-CoA thiolase 2, peroxisomal
35	TRINITY_DN33210_c3_g1	289	0.00	0.00	56.39	7.95	8.79	Peroxidase 4
36	TRINITY_DN30270_c0_g2	299	0.00	0.00	54.72	7.46	8.76	Peroxidase 71
37	TRINITY_DN34560_c2_g1	234	0.00	0.00	54.09	9.42	8.74	Protein PIN-LIKES 5
38	TRINITY_DN31660_c0_g4	578	0.00	0.00	51.59	3.64	8.68	Probable aldo-keto reductase 6
39	TRINITY_DN34587_c3_g2	480	0.00	0.00	51.60	4.38	8.68	Polyubiquitin 11
40	TRINITY_DN9695_c0_g2	606	0.00	0.00	51.06	3.43	8.66	Caffeic acid 3-O-methyltransferase
41	TRINITY_DN29985_c0_g3	964	0.41	0.02	51.13	2.16	8.66	Indole-3-acetic acid-induced protein ARG7
42	TRINITY_DN32193_c0_g2	808	0.00	0.00	48.66	2.45	8.60	Transmembrane protein 64
43	TRINITY_DN29384_c0_g2	2280	0.00	0.00	49.48	0.88	8.60	Vicilin-like antimicrobial peptides 2-2
44	TRINITY_DN7678_c0_g1	405	0.00	0.00	48.10	4.84	8.57	Bifunctional dethiobiotin synthetase/7,8-diamino-pelargonic acid aminotransferase, mitochondrial
45	TRINITY_DN33820_c1_g3	1645	0.00	0.00	47.58	1.18	8.57	Retrovirus-related Pol polyprotein from transposon TNT 1-94
46	TRINITY_DN18000_c0_g2	241	0.00	0.00	46.44	7.85	8.51	Protein DETOXIFICATION 28
47	TRINITY_DN25647_c0_g1	1064	0.35	0.01	44.78	1.71	8.48	pEARLI1-like lipid transfer protein 1
48	TRINITY_DN8869_c0_g1	474	0.00	0.00	43.81	3.77	8.44	Defensin-like protein 19
49	TRINITY_DN28967_c0_g2	1301	0.00	0.00	43.45	1.36	8.41	Sister chromatid cohesion 1 protein 2
50	TRINITY_DN34868_c0_g3	558	0.00	0.00	43.30	3.16	8.41	Riboflavin biosynthesis protein PYRR, chloroplastic

*logFC: the logarithm to base 2 of fold change (Salt/Control)

**TPM: transcripts per million

Supplementary Table 1. (Cont) Annotation profile of DEG in ice plant seedlings treated with 200 mM NaCl (|FC|>4, FDR<0.001)

No.	Transcript ID	Length	Control reads	Control reads (TPM**)	Salt reads	Salt reads (TPM)	logFC*	Annotation
51	TRINITY_DN32086_c0_g1	237	0.00	0.00	42.38	7.29	8.38	DEAD-box ATP-dependent RNA helicase 35
52	TRINITY_DN22511_c0_g2	682	0.00	0.00	42.47	2.54	8.38	Heavy metal-associated isoprenylated plant protein 7
53	TRINITY_DN32193_c0_g1	808	0.00	0.00	40.35	2.04	8.31	Transmembrane protein 64
54	TRINITY_DN32887_c2_g2	1494	6.78	0.20	2310.70	63.02	8.30	Probable aquaporin PIP1-4
55	TRINITY_DN14328_c0_g1	463	0.00	0.00	38.58	3.40	8.27	Vacuolar iron transporter homolog 1
56	TRINITY_DN34821_c1_g2	446	0.00	0.00	37.87	3.46	8.23	Cellulose synthase-like protein E1
57	TRINITY_DN35197_c0_g3	3697	0.00	0.00	38.13	0.42	8.23	Sister chromatid cohesion 1 protein 4
58	TRINITY_DN34338_c0_g1	892	0.00	0.00	37.61	1.72	8.23	Exocyst complex component EXO70A1
59	TRINITY_DN55762_c0_g1	450	0.00	0.00	36.58	3.31	8.19	Receptor-like protein kinase HSL1
60	TRINITY_DN25727_c0_g2	720	0.00	0.00	35.64	2.02	8.15	Thaumatin-like protein 1
61	TRINITY_DN29590_c0_g2	1524	0.08	0.00	36.33	0.97	8.15	Uncharacterized calcium-binding protein At1g02270
62	TRINITY_DN54533_c0_g1	362	0.00	0.00	35.55	4.00	8.15	Delta(12)-fatty-acid desaturase FAD2
63	TRINITY_DN33589_c0_g10	726	0.09	0.01	35.15	1.97	8.11	Protein TIC 40, chloroplastic
64	TRINITY_DN29633_c1_g1	259	0.00	0.00	35.03	5.51	8.11	GATA transcription factor 21
65	TRINITY_DN33576_c0_g1	276.5	0.00	0.00	35.19	4.34	8.11	Cytochrome c oxidase subunit 3
66	TRINITY_DN52284_c0_g1	237	0.00	0.00	33.64	5.78	8.07	37S ribosomal protein SWS2, mitochondrial
67	TRINITY_DN32012_c0_g2	845	0.00	0.00	33.74	1.63	8.07	Bifunctional protein FolD 1, mitochondrial
68	TRINITY_DN35256_c0_g1	448.6	0.00	0.00	33.65	2.37	8.07	Myosin regulatory light chain 2, ventricular/cardiac muscle isoform
69	TRINITY_DN18647_c0_g1	775	0.00	0.00	33.91	1.78	8.07	2-oxoglutarate-Fe(II) type oxidoreductase
70	TRINITY_DN719_c0_g1	819	0.00	0.00	32.85	1.63	8.03	Threonine--tRNA ligase, mitochondrial 1
71	TRINITY_DN31870_c0_g1	294	0.00	0.00	33.41	4.63	8.03	Xyloglucan endotransglucosylase/hydrolase 2
72	TRINITY_DN33386_c0_g1	297	0.00	0.00	33.17	4.55	8.03	UDP-glucose flavonoid 3-O-glucosyltransferase 7
73	TRINITY_DN18035_c0_g1	1112	1.03	0.04	296.12	10.85	8.00	Zinc finger A20 and AN1 domain-containing stress-associated protein 1
74	TRINITY_DN24274_c0_g3	986	1.31	0.06	295.95	12.23	8.00	Protein HHL1, chloroplastic
75	TRINITY_DN33589_c0_g7	726	0.08	0.00	31.13	1.75	7.94	Protein TIC 40, chloroplastic

*logFC: the logarithm to base 2 of fold change (Salt/Control)

**TPM: transcripts per million

Supplementary Table 1. (Cont) Annotation profile of DEG in ice plant seedlings treated with 200 mM NaCl (|FC|>4, FDR<0.001)

No.	Transcript ID	Length	Control reads	Control reads (TPM**)	Salt reads	Salt reads (TPM)	logFC*	Annotation
76	TRINITY_DN50802_c0_g1	293	0.00	0.00	31.17	4.34	7.94	Delta(12)-acyl-lipid-desaturase
77	TRINITY_DN8632_c0_g2	2156	0.00	0.00	30.67	0.58	7.94	Lysine-specific histone demethylase 1 homolog 1
78	TRINITY_DN26567_c2_g1	241	0.00	0.00	29.91	5.06	7.89	DNA repair endonuclease UVH1
79	TRINITY_DN33589_c0_g1	724	0.07	0.00	29.63	1.67	7.89	Protein TIC 40, chloroplastic
80	TRINITY_DN54664_c0_g1	438	0.00	0.00	29.64	2.76	7.89	Probable flavin-containing monooxygenase 1
81	TRINITY_DN42563_c1_g1	588	0.00	0.00	30.03	2.08	7.89	Ethylene-responsive transcription factor ERF094
82	TRINITY_DN42205_c0_g1	341	0.00	0.00	30.33	3.62	7.89	Putative cytochrome c biosynthesis ccmC-like mitochondrial protein
83	TRINITY_DN33589_c0_g2	724	0.08	0.00	29.65	1.67	7.89	Protein TIC 40, chloroplastic
84	TRINITY_DN32695_c0_g14	552	0.41	0.03	30.29	2.24	7.89	Cellulose synthase-like protein D1
85	TRINITY_DN44604_c0_g1	1992	0.01	0.00	29.75	0.61	7.89	Protein DETOXIFICATION 49
86	TRINITY_DN23995_c0_g1	480	0.00	0.00	28.89	2.45	7.84	Tetraspanin-7
87	TRINITY_DN22315_c0_g1	1141	0.00	0.00	29.10	1.04	7.84	Tetraspanin-19
88	TRINITY_DN33589_c0_g11	724	0.07	0.00	27.95	1.57	7.79	Protein TIC 40, chloroplastic
89	TRINITY_DN18089_c0_g1	364	0.00	0.00	27.89	3.12	7.79	Methylcrotonoyl-CoA carboxylase subunit alpha, mitochondrial
90	TRINITY_DN51283_c0_g1	275	0.00	0.00	27.61	4.09	7.79	Beta-glucosidase 44
91	TRINITY_DN30024_c0_g2	453	0.00	0.00	27.89	2.51	7.79	Probable 2-oxoglutarate-dependent dioxygenase At5g05600
92	TRINITY_DN35287_c0_g1	254	0.00	0.00	28.18	4.25	7.79	Myosin-7
93	TRINITY_DN34905_c0_g1	287	0.00	0.00	28.17	4.00	7.79	WAT1-related protein At5g40230
94	TRINITY_DN53810_c0_g1	429	0.00	0.00	26.96	2.56	7.74	Anthocyanidin 3-O-glucosyltransferase 5
95	TRINITY_DN31429_c0_g2	384	0.21	0.02	25.68	2.72	7.69	Probable fatty acyl-CoA reductase 4
96	TRINITY_DN33589_c0_g6	724	0.06	0.00	26.25	1.48	7.69	Protein TIC 40, chloroplastic
97	TRINITY_DN20595_c0_g1	480	0.00	0.00	25.73	2.18	7.69	Endochitinase EP3
98	TRINITY_DN29285_c0_g2	764	0.00	0.00	24.77	1.32	7.63	Cysteine-rich receptor-like protein kinase 8
99	TRINITY_DN40554_c0_g1	232	0.00	0.00	23.96	4.21	7.57	Mitochondrial inner membrane protease subunit 1
100	TRINITY_DN55104_c0_g1	397	0.00	0.00	24.27	2.49	7.57	Germin-like protein 8-2

*logFC: the logarithm to base 2 of fold change (Salt/Control)

**TPM: transcripts per million

Supplementary Table 1. (Cont) Annotation profile of DEG in ice plant seedlings treated with 200 mM NaCl (|FC|>4, FDR<0.001)

No.	Transcript ID	Length	Control reads	Control reads (TPM**)	Salt reads	Salt reads (TPM)	logFC*	Annotation
101	TRINITY_DN36009_c0_g1	342	0.00	0.00	23.77	2.83	7.57	Metal transporter Nramp5
102	TRINITY_DN8294_c0_g2	1628	0.00	0.00	24.39	0.61	7.57	Polygalacturonase inhibitor
103	TRINITY_DN39372_c0_g1	254	0.00	0.00	24.08	3.86	7.57	DNA topoisomerase 2
104	TRINITY_DN53709_c0_g1	233	0.00	0.00	24.25	4.24	7.57	Transcription factor MYB78
105	TRINITY_DN35226_c1_g1	1062	0.00	0.00	24.26	0.93	7.57	Pentatricopeptide repeat-containing protein At1g20230
106	TRINITY_DN8937_c0_g1	765	0.00	0.00	24.49	1.30	7.57	Polygalacturonase
107	TRINITY_DN26806_c0_g2	1708	1.36	0.04	215.13	5.13	7.54	Protein NUCLEAR FUSION DEFECTIVE 4
108	TRINITY_DN5449_c0_g1	320	0.00	0.00	22.51	2.87	7.51	Gibberellin-regulated protein 6
109	TRINITY_DN23565_c1_g1	252	0.00	0.00	23.10	3.74	7.51	G-type lectin S-receptor-like serine/threonine-protein kinase
110	TRINITY_DN31429_c1_g1	303	0.00	0.00	22.78	3.06	7.51	Fatty acyl-CoA reductase 1
111	TRINITY_DN15146_c0_g2	518	0.00	0.00	22.64	1.78	7.51	Protein DETOXIFICATION 40
112	TRINITY_DN57957_c0_g1	276	0.00	0.00	22.89	3.38	7.51	Antimicrobial peptide 1
113	TRINITY_DN34096_c0_g3	320	0.00	0.00	21.76	2.77	7.45	Protein SRG1
114	TRINITY_DN7724_c1_g1	232	0.00	0.00	22.49	3.95	7.45	Protein NEN2
115	TRINITY_DN25562_c0_g1	680	0.00	0.00	21.96	1.32	7.45	Putative pectinesterase 11
116	TRINITY_DN35287_c0_g3	394.33	0.00	0.00	22.46	2.05	7.45	Myosin-7
117	TRINITY_DN54904_c0_g1	329	0.00	0.00	21.62	2.68	7.45	Kallikrein-14
118	TRINITY_DN1826_c0_g2	1536	0.04	0.00	21.50	0.57	7.45	UPF0496 protein 4
119	TRINITY_DN32683_c0_g2	650	0.00	0.00	22.03	1.38	7.45	Probable serine/threonine-protein kinase DDB_G0272254
120	TRINITY_DN14025_c1_g1	270	0.00	0.00	22.41	3.38	7.45	E3 ubiquitin-protein ligase RKP
121	TRINITY_DN35424_c1_g3	503.15	0.00	0.00	21.07	0.86	7.38	Myosin-7
122	TRINITY_DN6738_c0_g1	240	0.00	0.00	20.91	3.55	7.38	Cellulose synthase-like protein D1
123	TRINITY_DN40692_c0_g1	251	0.00	0.00	21.07	3.42	7.38	54S ribosomal protein RTC6, mitochondrial
124	TRINITY_DN8288_c0_g2	1639	0.01	0.00	21.41	0.53	7.38	Hydroxyproline O-galactosyltransferase GALT2
125	TRINITY_DN35199_c0_g1	282	0.00	0.00	20.55	2.97	7.38	Leucine-rich repeat receptor-like serine/threonine-protein kinase BAM1

*logFC: the logarithm to base 2 of fold change (Salt/Control)

**TPM: transcripts per million

Supplementary Table 1. (Cont) Annotation profile of DEG in ice plant seedlings treated with 200 mM NaCl (|FC|>4, FDR<0.001)

No.	Transcript ID	Length	Control reads	Control reads (TPM**)	Salt reads	Salt reads (TPM)	logFC*	Annotation
126	TRINITY_DN46502_c0_g1	246	0.00	0.00	20.77	3.44	7.38	ABC transporter G family member 22
127	TRINITY_DN50314_c0_g1	239	0.00	0.00	20.78	3.54	7.38	Cytochrome P450 711A1
128	TRINITY_DN25719_c1_g1	478	0.00	0.00	20.74	1.77	7.38	Zinc finger protein ZAT5
129	TRINITY_DN32695_c0_g1	552	0.41	0.03	19.97	1.47	7.31	Cellulose synthase-like protein D1
130	TRINITY_DN34275_c2_g3	495	0.01	0.00	19.63	1.62	7.31	E3 ubiquitin-protein ligase ATL9
131	TRINITY_DN34738_c1_g3	970	0.00	0.00	20.32	0.85	7.31	E3 ubiquitin-protein ligase SHPRH
132	TRINITY_DN23776_c0_g2	280	0.00	0.00	20.26	2.95	7.31	Omega-6 fatty acid desaturase, endoplasmic reticulum isozyme 2
133	TRINITY_DN42510_c0_g1	459	0.00	0.00	19.59	1.74	7.31	G-type lectin S-receptor-like serine/threonine-protein kinase SD1-13
134	TRINITY_DN34742_c0_g2	676	0.00	0.00	19.74	1.19	7.31	ABC transporter B family member 26, chloroplastic
135	TRINITY_DN26065_c0_g3	918	0.00	0.00	20.23	0.90	7.31	Peroxisomal membrane protein PMP22
136	TRINITY_DN55539_c0_g1	290	0.00	0.00	19.90	2.80	7.31	40S ribosomal protein S3-A
137	TRINITY_DN35110_c10_g1	245	0.00	0.00	20.42	3.40	7.31	AT-hook motif nuclear-localized protein 20
138	TRINITY_DN34196_c0_g2	386	0.00	0.00	19.92	1.43	7.31	Cytochrome c oxidase subunit 2
139	TRINITY_DN27215_c0_g1	294	0.00	0.00	18.83	2.61	7.24	Probable NADP-dependent mannitol dehydrogenase
140	TRINITY_DN35295_c2_g7	429	0.00	0.00	19.08	1.81	7.24	Squalene synthase 1
141	TRINITY_DN26571_c0_g3	618	0.02	0.00	18.51	1.22	7.24	E3 ubiquitin-protein ligase PUB22
142	TRINITY_DN31840_c0_g5	442	0.00	0.00	19.34	1.78	7.24	Peroxidase 16
143	TRINITY_DN24370_c0_g1	425	0.00	0.00	18.73	1.80	7.24	Peroxidase 57
144	TRINITY_DN41470_c0_g9	676	0.00	0.00	18.60	1.12	7.24	Mitochondrial import inner membrane translocase subunit TIM8
145	TRINITY_DN32292_c0_g3	294	0.00	0.00	18.99	2.63	7.24	Ceramide kinase
146	TRINITY_DN28025_c1_g1	336	0.00	0.00	18.86	2.29	7.24	Polyvinylalcohol dehydrogenase
147	TRINITY_DN52352_c0_g1	284	0.00	0.00	19.38	2.78	7.24	RNA polymerase sigma factor sigA
148	TRINITY_DN42942_c0_g1	233	0.00	0.00	18.55	3.24	7.24	Pleiotropic drug resistance protein 2
149	TRINITY_DN43931_c0_g1	260	0.00	0.00	18.88	2.96	7.24	Camelliol C synthase
150	TRINITY_DN17833_c0_g1	442	0.00	0.00	19.22	1.77	7.24	Probable pectinesterase 68

*logFC: the logarithm to base 2 of fold change (Salt/Control)

**TPM: transcripts per million

Supplementary Table 1. (Cont) Annotation profile of DEG in ice plant seedlings treated with 200 mM NaCl (|FC|>4, FDR<0.001)

No.	Transcript ID	Length	Control reads	Control reads (TPM**)	Salt reads	Salt reads (TPM)	logFC*	Annotation
151	TRINITY_DN48478_c0_g1	485	0.00	0.00	19.24	1.62	7.24	Probable cyclic nucleotide-gated ion channel 14
152	TRINITY_DN30101_c0_g2	1185	0.77	0.03	169.00	5.81	7.19	Caffeic acid 3-O-methyltransferase
153	TRINITY_DN26671_c1_g1	252	0.00	0.00	17.97	2.91	7.16	V-type proton ATPase 16 kDa proteolipid subunit
154	TRINITY_DN52245_c0_g1	333	0.00	0.00	17.54	2.15	7.16	Protein TIC 100
155	TRINITY_DN39777_c0_g1	320	0.00	0.00	17.90	2.28	7.16	ATP synthase subunit a
156	TRINITY_DN33330_c1_g1	250	0.00	0.00	18.41	3.00	7.16	Nuclear pore complex protein GP210
157	TRINITY_DN43781_c0_g1	290	0.00	0.00	17.69	2.49	7.16	E3 ubiquitin-protein ligase SINAT5
158	TRINITY_DN40742_c0_g1	370	0.00	0.00	17.69	1.95	7.16	Pantothenate transporter FEN2
159	TRINITY_DN897_c0_g1	235	0.00	0.00	18.21	3.16	7.16	Pentatricopeptide repeat-containing protein At3g12770
160	TRINITY_DN35810_c0_g1	342	0.00	0.00	17.89	2.13	7.16	Probable serine/threonine-protein kinase PBL22
161	TRINITY_DN35247_c0_g5	442	0.00	0.00	17.99	1.66	7.16	Phospholipid-transporting ATPase 2
162	TRINITY_DN57630_c0_g1	261	0.00	0.00	17.56	2.74	7.16	Probable polyol transporter 4
163	TRINITY_DN24045_c0_g3	842	0.00	0.00	18.50	0.90	7.16	Glucan endo-1,3-beta-glucosidase 8
164	TRINITY_DN28686_c0_g1	2192	0.01	0.00	17.82	0.33	7.16	Protein trichome birefringence-like 11
165	TRINITY_DN55500_c0_g1	262	0.00	0.00	17.63	2.74	7.16	MDIS1-interacting receptor like kinase 1
166	TRINITY_DN3261_c0_g1	335	0.00	0.00	17.27	2.10	7.08	Uncharacterized RNA-binding protein C23A1.09
167	TRINITY_DN9773_c0_g1	268	0.00	0.00	16.64	2.53	7.08	Wall-associated receptor kinase-like 9
168	TRINITY_DN53250_c0_g1	268	0.00	0.00	16.84	2.56	7.08	Protein NRT1/ PTR FAMILY 3.1
169	TRINITY_DN32920_c0_g4	441	0.00	0.00	17.32	1.60	7.08	DNA polymerase epsilon catalytic subunit A
170	TRINITY_DN44781_c0_g1	245	0.00	0.00	17.08	2.84	7.08	Pentatricopeptide repeat-containing protein At1g11290, chloroplastic
171	TRINITY_DN26034_c0_g1	621	0.00	0.00	17.17	1.13	7.08	Zinc finger protein WIP3
172	TRINITY_DN35025_c12_g1	301	0.00	0.00	17.09	2.31	7.08	3,9-dihydroxypterocarpan 6A-monooxygenase
173	TRINITY_DN12425_c0_g1	501	0.00	0.00	17.40	1.42	7.08	Caffeoylshikimate esterase
174	TRINITY_DN26161_c3_g1	294	0.00	0.00	16.55	2.29	7.08	Pentatricopeptide repeat-containing protein At4g35130, chloroplastic
175	TRINITY_DN57040_c0_g1	345	0.00	0.00	16.83	1.99	7.08	UDP-glycosyltransferase 73B3

*logFC: the logarithm to base 2 of fold change (Salt/Control)

**TPM: transcripts per million

Supplementary Table 1. (Cont) Annotation profile of DEG in ice plant seedlings treated with 200 mM NaCl (|FC|>4, FDR<0.001)

No.	Transcript ID	Length	Control reads	Control reads (TPM**)	Salt reads	Salt reads (TPM)	logFC*	Annotation
176	TRINITY_DN14398_c0_g2	338	0.00	0.00	17.24	2.08	7.08	NAD(P)H-quinone oxidoreductase subunit 5, chloroplastic
177	TRINITY_DN39076_c0_g1	314	0.00	0.00	16.61	2.16	7.08	Pentatricopeptide repeat-containing protein At2g22070
178	TRINITY_DN50886_c0_g1	376	0.00	0.00	16.77	1.82	7.08	Deoxyribonuclease Tat-D
179	TRINITY_DN30389_c1_g1	257	0.00	0.00	17.14	2.72	7.08	Probable leucine-rich repeat receptor-like protein kinase At5g63930
180	TRINITY_DN51754_c0_g1	232	0.00	0.00	16.72	2.94	7.08	Agamous-like MADS-box protein AGL16
181	TRINITY_DN52039_c0_g1	275	0.00	0.00	16.98	2.52	7.08	Leptomycin B resistance protein pmd1
182	TRINITY_DN41151_c0_g1	252	0.00	0.00	17.12	2.77	7.08	Fructose-bisphosphate aldolase
183	TRINITY_DN15133_c0_g1	422	0.00	0.00	17.17	1.66	7.08	Translationally-controlled tumor protein
184	TRINITY_DN31773_c0_g2	1200	0.00	0.00	17.18	0.58	7.08	Serine/threonine-protein kinase 24
185	TRINITY_DN30673_c3_g1	460	0.00	0.00	16.79	1.49	7.08	DmX-like protein 1
186	TRINITY_DN33773_c1_g2	273	0.00	0.00	16.82	2.51	7.08	LEAF RUST 10 DISEASE-RESISTANCE LOCUS RECEPTOR-LIKE PROTEIN KINASE-like 2.5
187	TRINITY_DN37613_c0_g1	448	0.00	0.00	16.83	1.53	7.08	Polygalacturonase inhibitor
188	TRINITY_DN23953_c0_g2	1162	0.00	0.00	17.41	0.61	7.08	Tetraketide alpha-pyrone reductase 1
189	TRINITY_DN43806_c0_g1	287	0.00	0.00	17.06	2.42	7.08	Proline-rich receptor-like protein kinase PERK4
190	TRINITY_DN43456_c0_g1	374	0.00	0.00	17.16	1.87	7.08	Cytochrome c oxidase subunit NDUFA4
191	TRINITY_DN51700_c0_g1	240	0.00	0.00	17.06	2.90	7.08	Delta-aminolevulinic acid dehydratase
192	TRINITY_DN22623_c0_g2	508	0.00	0.00	16.56	1.33	7.08	Copia protein
193	TRINITY_DN27686_c1_g1	1864	0.99	0.02	148.92	3.26	7.01	Nuclear inhibitor of protein phosphatase 1
194	TRINITY_DN2948_c0_g1	644	0.00	0.00	16.20	1.03	6.99	Dihydrolipoyllysine-residue acetyltransferase component of pyruvate dehydrogenase complex, mitochondrial
195	TRINITY_DN38881_c0_g1	254	0.00	0.00	16.37	2.63	6.99	Proteasome subunit alpha type-4-2
196	TRINITY_DN37167_c0_g1	328	0.00	0.00	15.67	1.95	6.99	Glucomannan 4-beta-mannosyltransferase 9
197	TRINITY_DN16688_c0_g1	518	0.00	0.00	16.09	1.27	6.99	Secologanin synthase
198	TRINITY_DN24487_c0_g2	2141	0.00	0.00	16.17	0.31	6.99	Protein NRT1/ PTR FAMILY 1.2
199	TRINITY_DN22718_c1_g1	385	0.00	0.00	15.64	1.66	6.99	Stomatin-like protein 2, mitochondrial
200	TRINITY_DN20995_c0_g2	916	0.00	0.00	16.30	0.73	6.99	Polygalacturonase

*logFC: the logarithm to base 2 of fold change (Salt/Control)

**TPM: transcripts per million

Supplementary Table 1. (Cont) Annotation profile of DEG in ice plant seedlings treated with 200 mM NaCl (|FC|>4, FDR<0.001)

No.	Transcript ID	Length	Control reads	Control reads (TPM**)	Salt reads	Salt reads (TPM)	logFC*	Annotation
201	TRINITY_DN56613_c0_g1	258	0.00	0.00	16.32	2.58	6.99	Collagen alpha-1(III) chain
202	TRINITY_DN31525_c0_g3	1241	0.00	0.00	16.22	0.53	6.99	F-box/kelch-repeat protein At3g23880
203	TRINITY_DN43573_c0_g1	335	0.00	0.00	16.47	2.00	6.99	High affinity sulfate transporter 1
204	TRINITY_DN39259_c0_g1	376	0.00	0.00	16.07	1.74	6.99	GCR1-dependent translation factor 1
205	TRINITY_DN18966_c0_g1	376	0.00	0.00	15.73	1.70	6.99	Cytochrome c oxidase subunit 3
206	TRINITY_DN42320_c0_g2	367	0.00	0.00	15.74	1.75	6.99	RING-H2 finger protein ATL54
207	TRINITY_DN44189_c0_g1	295	0.00	0.00	16.15	2.23	6.99	DNA mismatch repair protein MSH6
208	TRINITY_DN51290_c0_g1	235	0.00	0.00	15.89	2.76	6.99	NifU-like protein C1709.19c
209	TRINITY_DN1430_c0_g1	518	0.00	0.00	15.68	1.23	6.99	RING-H2 finger protein ATL20
210	TRINITY_DN37170_c0_g1	241	0.00	0.00	15.58	2.63	6.99	Light-harvesting complex-like protein 3 isotype 1, chloroplastic
211	TRINITY_DN28040_c0_g1	260	0.00	0.00	16.37	2.57	6.99	ABC transporter G family member 14
212	TRINITY_DN49545_c0_g1	323	0.00	0.00	15.51	1.96	6.99	Endoribonuclease Dicer homolog 4
213	TRINITY_DN25414_c3_g1	233	0.00	0.00	15.69	2.74	6.99	Homeobox-leucine zipper protein HDG11
214	TRINITY_DN35034_c4_g1	279	0.00	0.00	15.68	2.29	6.99	Putative receptor protein kinase ZmPK1
215	TRINITY_DN38940_c0_g1	395	0.00	0.00	15.53	1.60	6.99	LEAF RUST 10 DISEASE-RESISTANCE LOCUS RECEPTOR-LIKE PROTEIN KINASE-like 1.2
216	TRINITY_DN4996_c1_g1	296	0.00	0.00	15.26	2.10	6.90	2-oxoisovalerate dehydrogenase subunit alpha 2, mitochondrial
217	TRINITY_DN1297_c0_g1	248	0.00	0.00	14.73	2.42	6.90	Retrovirus-related Pol polyprotein from transposon TNT 1-94
218	TRINITY_DN23146_c0_g1	267.33	0.00	0.00	14.61	1.59	6.90	Myosin-7
219	TRINITY_DN2779_c0_g2	258	0.00	0.00	15.03	2.37	6.90	Putative ribonuclease H protein At1g65750
220	TRINITY_DN21429_c0_g1	270	0.00	0.00	14.99	2.26	6.90	Uncharacterized membrane protein At3g27390
221	TRINITY_DN3703_c1_g1	242	0.00	0.00	15.00	2.53	6.90	Linoleate 9S-lipoxygenase 5, chloroplastic
222	TRINITY_DN32358_c0_g2	701	0.00	0.00	15.20	0.88	6.90	Exocyst complex component SEC6
223	TRINITY_DN34226_c0_g1	558.17	0.00	0.00	14.80	0.81	6.90	Troponin C, slow skeletal and cardiac muscles
224	TRINITY_DN4828_c0_g1	276	0.00	0.00	15.34	2.26	6.90	NADH-ubiquinone oxidoreductase chain 6
225	TRINITY_DN51607_c0_g1	303	0.00	0.00	15.08	2.03	6.90	Cytochrome P450 83B1

*logFC: the logarithm to base 2 of fold change (Salt/Control)

**TPM: transcripts per million

Supplementary Table 1. (Cont) Annotation profile of DEG in ice plant seedlings treated with 200 mM NaCl (|FC|>4, FDR<0.001)

No.	Transcript ID	Length	Control reads	Control reads (TPM**)	Salt reads	Salt reads (TPM)	logFC*	Annotation
226	TRINITY_DN30997_c0_g1	434	0.00	0.00	14.64	1.37	6.90	EPIDERMAL PATTERNING FACTOR-like protein 8
227	TRINITY_DN34063_c0_g3	527	0.00	0.00	14.84	1.15	6.90	Calpain-type cysteine protease DEK1
228	TRINITY_DN3849_c0_g2	883	0.00	0.00	14.95	0.69	6.90	Triose phosphate/phosphate translocator, chloroplastic
229	TRINITY_DN33913_c0_g1	2265	0.00	0.00	15.24	0.27	6.90	C2 and GRAM domain-containing protein At5g50170
230	TRINITY_DN39706_c0_g1	274	0.00	0.00	14.59	2.17	6.90	Exopolygalacturonase clone GBGA483
231	TRINITY_DN25934_c1_g1	257	0.00	0.00	15.22	2.41	6.90	Multiple organellar RNA editing factor 8, chloroplastic/mitochondrial
232	TRINITY_DN34296_c0_g1	348	0.00	0.00	15.07	1.76	6.90	Tetraketide alpha-pyrone reductase 1
233	TRINITY_DN25088_c1_g1	426	0.00	0.00	15.01	1.44	6.90	Cytochrome P450 94C1
234	TRINITY_DN13223_c0_g1	750	0.32	0.02	14.99	0.81	6.90	E3 ubiquitin-protein ligase ATL6
235	TRINITY_DN9827_c0_g1	265	0.00	0.00	14.94	2.30	6.90	ATP-dependent DNA helicase DDX11
236	TRINITY_DN44760_c0_g1	351	0.00	0.00	14.80	1.72	6.90	Protein NUCLEAR FUSION DEFECTIVE 4
237	TRINITY_DN7605_c0_g1	486	0.00	0.00	14.83	1.24	6.90	Inactive protein RESTRICTED TEV MOVEMENT 1
238	TRINITY_DN23055_c2_g1	244	0.00	0.00	14.70	2.46	6.90	Pentatricopeptide repeat-containing protein At3g22690
239	TRINITY_DN29246_c0_g1	1359	0.00	0.00	14.85	0.45	6.90	Probable dolichyl pyrophosphate Man9GlcNAc2 alpha-1,3-glucosyltransferase
240	TRINITY_DN34353_c0_g2	590	0.02	0.00	14.55	1.00	6.90	Hydroxyproline O-galactosyltransferase HPGT3
241	TRINITY_DN24942_c0_g2	237	0.71	0.13	104.80	18.02	6.51	Expansin-A8
242	TRINITY_DN31880_c0_g1	244	2.55	0.46	255.29	42.63	6.31	Tuberculostearic acid methyltransferase UfaA1
243	TRINITY_DN27659_c0_g2	266	1.34	0.22	70.84	10.85	5.94	Histidine-containing phosphotransfer protein 2
244	TRINITY_DN30101_c0_g1	1177	0.74	0.03	71.44	2.47	5.94	Caffeic acid 3-O-methyltransferase
245	TRINITY_DN31048_c0_g1	259	1.83	0.31	118.29	18.61	5.76	UDP-glucosyl transferase 73B2
246	TRINITY_DN52613_c0_g5	727	11.05	0.67	552.19	30.95	5.59	Glycine-rich RNA-binding protein RZ1A
247	TRINITY_DN22554_c0_g2	757	2.83	0.17	150.46	8.10	5.55	Probable beta-1,3-galactosyltransferase 14
248	TRINITY_DN22359_c0_g1	1432	1.24	0.04	51.74	1.47	5.50	DNA polymerase zeta processivity subunit
249	TRINITY_DN13039_c0_g1	256	1.10	0.19	49.08	7.81	5.41	Fructose-bisphosphate aldolase 1, chloroplastic
250	TRINITY_DN22389_c0_g1	1959	2.37	0.05	91.55	1.90	5.40	Lipoamide acyltransferase component of branched-chain alpha-keto acid dehydrogenase complex, mitochondrial

*logFC: the logarithm to base 2 of fold change (Salt/Control)

**TPM: transcripts per million

Supplementary Table 1. (Cont) Annotation profile of DEG in ice plant seedlings treated with 200 mM NaCl (|FC|>4, FDR<0.001)

No.	Transcript ID	Length	Control reads	Control reads (TPM**)	Salt reads	Salt reads (TPM)	logFC*	Annotation
251	TRINITY_DN17115_c0_g3	472	75.95	7.12	3247.58	280.36	5.37	Glyceraldehyde-3-phosphate dehydrogenase GAPB, chloroplastic
252	TRINITY_DN52613_c0_g4	737	1.55	0.09	86.99	4.81	5.32	Glycine-rich RNA-binding protein RZ1A
253	TRINITY_DN34397_c0_g4	3799	1.16	0.01	43.39	0.47	5.22	Symplekin
254	TRINITY_DN30277_c0_g2	1791	2.26	0.06	75.08	1.71	5.10	Protein disulfide isomerase-like 1-3
255	TRINITY_DN20008_c0_g1	1264	2.63	0.09	108.83	3.51	5.09	Cysteine-rich receptor-like protein kinase 25
256	TRINITY_DN27474_c0_g1	235	2.16	0.41	73.68	12.77	5.08	Pathogenesis-related protein STH-21
257	TRINITY_DN15425_c0_g1	263	0.52	0.09	38.54	5.97	5.08	NADPH-dependent aldo-keto reductase, chloroplastic
258	TRINITY_DN35357_c2_g1	245	2.23	0.40	72.92	12.13	5.07	Alpha-amylase isozyme 3B
259	TRINITY_DN9438_c0_g1	3384	13.19	0.17	447.42	5.39	5.05	Protein LHY
260	TRINITY_DN35406_c0_g3	245	1.92	0.35	69.63	11.58	5.00	Twinkle homolog protein, chloroplastic/mitochondrial
261	TRINITY_DN27879_c0_g1	312	1.82	0.26	67.64	8.83	4.96	Germin-like protein subfamily 2 member 4
262	TRINITY_DN32499_c0_g3	830	0.74	0.04	34.65	1.70	4.93	Peroxidase 27
263	TRINITY_DN32767_c1_g2	830	3.10	0.17	96.09	4.72	4.90	Defensin-like protein 182
264	TRINITY_DN35326_c1_g3	1368	2.49	0.08	62.48	1.86	4.83	ABC transporter C family member 2
265	TRINITY_DN30100_c0_g1	248	1.02	0.18	31.90	5.24	4.80	Endonuclease 4
266	TRINITY_DN34889_c1_g1	236	1.75	0.33	60.46	10.44	4.78	Vacuolar iron transporter homolog 3
267	TRINITY_DN24973_c0_g3	1353	1.10	0.04	30.59	0.92	4.75	DExH-box ATP-dependent RNA helicase DExH8
268	TRINITY_DN41360_c0_g1	857	1.46	0.08	29.85	1.42	4.70	Peroxidase 5
269	TRINITY_DN2016_c0_g1	258	1.12	0.19	29.24	4.62	4.66	Actin-depolymerizing factor 2
270	TRINITY_DN33453_c0_g1	930	0.55	0.03	29.10	1.28	4.66	NDR1/HIN1-like protein 1
271	TRINITY_DN5416_c0_g1	266	1.08	0.18	28.43	4.35	4.61	RING-box protein 1A
272	TRINITY_DN31525_c0_g2	1450	2.30	0.07	53.22	1.50	4.60	F-box/kelch-repeat protein At3g23880
273	TRINITY_DN31807_c0_g3	238	4.27	0.79	101.82	17.43	4.59	18.1 kDa class I heat shock protein
274	TRINITY_DN34215_c0_g3	701	1.33	0.08	27.19	1.58	4.55	Carbon catabolite repressor protein 4 homolog 4
275	TRINITY_DN6500_c0_g1	621	1.79	0.13	51.34	3.37	4.55	Protein C2-DOMAIN ABA-RELATED 4

*logFC: the logarithm to base 2 of fold change (Salt/Control)

**TPM: transcripts per million

Supplementary Table 1. (Cont) Annotation profile of DEG in ice plant seedlings treated with 200 mM NaCl (|FC|>4, FDR<0.001)

No.	Transcript ID	Length	Control reads	Control reads (TPM**)	Salt reads	Salt reads (TPM)	logFC*	Annotation
276	TRINITY_DN35030_c0_g2	1089	2.50	0.10	50.29	1.88	4.52	Probable lysophospholipase BODYGUARD 4
277	TRINITY_DN31640_c0_g1	701	2.09	0.13	49.99	2.91	4.52	DNA topoisomerase 3-alpha
278	TRINITY_DN29987_c0_g1	3552	7.31	0.09	167.29	1.92	4.51	E3 ubiquitin-protein ligase BRE1-like 2
279	TRINITY_DN17115_c0_g1	487	0.53	0.05	25.78	2.16	4.50	Glyceraldehyde-3-phosphate dehydrogenase GAPB, chloroplastic
280	TRINITY_DN29512_c0_g1	788	1.47	0.08	26.42	1.37	4.50	3-ketoacyl-CoA synthase 19
281	TRINITY_DN33064_c2_g1	608	8.09	0.59	186.51	12.50	4.48	Organic cation/carnitine transporter 4
282	TRINITY_DN27124_c0_g1	1254	2.97	0.10	70.68	2.30	4.47	Late embryogenesis abundant protein At1g64065
283	TRINITY_DN3770_c0_g1	1213	2.24	0.08	47.34	1.59	4.43	Potassium channel AKT1
284	TRINITY_DN33907_c0_g3	985	2.39	0.11	46.13	1.91	4.40	AAA-ATPase At2g46620
285	TRINITY_DN25039_c0_g2	1331	1.29	0.04	24.07	0.74	4.38	Anaphase-promoting complex subunit 5
286	TRINITY_DN33877_c2_g2	1153	14.21	0.55	301.68	10.66	4.38	Transcription factor ICE1
287	TRINITY_DN34393_c6_g1	251	10.46	1.84	214.66	34.85	4.37	Bidirectional sugar transporter SWEET12
288	TRINITY_DN35387_c0_g2	1475.4	35.77	1.11	760.28	21.61	4.35	Delta(12)-fatty-acid desaturase FAD2
289	TRINITY_DN26840_c0_g1	985	2.88	0.13	65.10	2.69	4.34	Mitochondrial intermembrane space import and assembly protein 40 homolog
290	TRINITY_DN35033_c0_g1	239	5.73	1.06	125.32	21.37	4.31	Probable aldo-keto reductase 2
291	TRINITY_DN35246_c1_g1	232	2.27	0.43	42.52	7.47	4.30	Putative cysteine-rich receptor-like protein kinase 35
292	TRINITY_DN22209_c0_g1	711	4.33	0.27	82.93	4.75	4.29	Probable aldo-keto reductase 4
293	TRINITY_DN5954_c0_g1	249	1.09	0.19	21.59	3.53	4.26	Nuclear movement protein nudC
294	TRINITY_DN26476_c0_g1	530	0.54	0.05	22.46	1.73	4.26	Gibberellin-regulated protein 6
295	TRINITY_DN17783_c0_g2	848	7.98	0.42	159.36	7.66	4.25	Thaumatin-like protein
296	TRINITY_DN30280_c2_g1	247	8.59	1.54	172.77	28.50	4.21	Cytochrome c-2
297	TRINITY_DN25619_c0_g5	966	3.37	0.15	59.05	2.49	4.20	ATP-dependent Clp protease proteolytic subunit 2, mitochondrial
298	TRINITY_DN27174_c0_g1	724	4.12	0.25	74.48	4.19	4.13	Probable 2-oxoglutarate-dependent dioxygenase ANS
299	TRINITY_DN55783_c0_g1	252	1.17	0.21	20.16	3.26	4.12	Protein RALF-like 22
300	TRINITY_DN31765_c0_g1	256	1.18	0.20	19.55	3.11	4.12	40S ribosomal protein S27-2

*logFC: the logarithm to base 2 of fold change (Salt/Control)

**TPM: transcripts per million

Supplementary Table 1. (Cont) Annotation profile of DEG in ice plant seedlings treated with 200 mM NaCl (|FC|>4, FDR<0.001)

No.	Transcript ID	Length	Control reads	Control reads (TPM**)	Salt reads	Salt reads (TPM)	logFC*	Annotation
301	TRINITY_DN55608_c0_g1	260	8.53	1.45	155.78	24.41	4.06	Probable aquaporin PIP2-2
302	TRINITY_DN19168_c0_g1	1736	1.21	0.03	19.01	0.45	4.05	Serine/threonine-protein kinase OXI1
303	TRINITY_DN29298_c1_g1	251	1.03	0.18	18.92	3.07	4.05	Squalene synthase 1
304	TRINITY_DN4295_c0_g3	1161	1.47	0.06	18.56	0.65	4.05	DNA repair protein XRCC3 homolog
305	TRINITY_DN33645_c1_g2	261	3.28	0.56	53.34	8.33	4.05	Long-chain-alcohol oxidase FAO1
306	TRINITY_DN34758_c1_g1	1378	4.93	0.16	85.25	2.52	4.01	Dehydrodolichyl diphosphate synthase 2
307	TRINITY_DN29468_c0_g1	1737	12.89	0.33	214.32	5.03	3.99	Ethylene-responsive transcription factor ERF061
308	TRINITY_DN18720_c0_g1	619	8.55	0.61	142.79	9.40	3.93	Defensin-like protein 19
309	TRINITY_DN34625_c0_g1	1024	14.15	0.61	220.79	8.79	3.93	Endochitinase EP3
310	TRINITY_DN52889_c0_g1	743	181.11	10.78	2799.86	153.55	3.91	Wound-induced protein WIN1
311	TRINITY_DN33164_c1_g2	749	4.87	0.29	78.11	4.25	3.89	Brefeldin A-inhibited guanine nucleotide-exchange protein 1
312	TRINITY_DN4475_c0_g2	727	2.36	0.14	32.07	1.80	3.88	Stress-response A/B barrel domain-containing protein At5g22580
313	TRINITY_DN22686_c0_g1	509	2.29	0.20	32.41	2.59	3.88	Protein SAR DEFICIENT 1
314	TRINITY_DN32035_c0_g1	550	1.85	0.15	32.17	3.77	3.88	Germin-like protein 12-1
315	TRINITY_DN13270_c0_g1	461	2.43	0.23	31.23	2.76	3.83	Aldehyde dehydrogenase family 7 member A1
316	TRINITY_DN30814_c0_g4	3376	3.51	0.05	59.07	0.71	3.80	Pentatricopeptide repeat-containing protein At2g42920, chloroplastic
317	TRINITY_DN27522_c0_g1	668	5.12	0.34	72.92	4.45	3.79	Peroxidase 12
318	TRINITY_DN47970_c0_g1	1026	36.67	1.58	523.97	20.81	3.78	Auxin-binding protein ABP19a
319	TRINITY_DN32395_c0_g1	212	2.33	0.49	28.98	5.57	3.74	MLP-like protein 43
320	TRINITY_DN7042_c0_g1	1322	24.22	0.81	324.45	10.00	3.71	Peroxidase 4
321	TRINITY_DN33963_c1_g3	232	2.76	0.53	41.40	7.27	3.68	Cytosolic sulfotransferase 8
322	TRINITY_DN25719_c0_g1	564	4.78	0.38	66.24	4.79	3.65	Zinc finger protein ZAT5
323	TRINITY_DN7583_c0_g1	1100	3.06	0.12	39.98	1.48	3.64	AAA-ATPase At2g46620
324	TRINITY_DN35231_c1_g1	233	2.40	0.46	27.39	4.79	3.63	Spermidine hydroxycinnamoyl transferase
325	TRINITY_DN22456_c0_g1	1979	20.78	0.46	267.28	5.50	3.62	Leucine-rich repeat extensin-like protein 2

*logFC: the logarithm to base 2 of fold change (Salt/Control)

**TPM: transcripts per million

Supplementary Table 1. (Cont) Annotation profile of DEG in ice plant seedlings treated with 200 mM NaCl (|FC|>4, FDR<0.001)

No.	Transcript ID	Length	Control reads	Control reads (TPM**)	Salt reads	Salt reads (TPM)	logFC*	Annotation
326	TRINITY_DN34991_c0_g3	848	11.58	0.60	152.57	7.33	3.62	Dehydrodolichyl diphosphate synthase 2
327	TRINITY_DN6358_c0_g1	258	10.15	1.74	126.71	20.01	3.61	Beta carbonic anhydrase 2, chloroplastic
328	TRINITY_DN28755_c2_g1	607	5.84	0.43	75.78	5.09	3.60	Glucosidase 2 subunit beta
329	TRINITY_DN4129_c1_g1	554	2.23	0.18	25.99	1.91	3.58	Zinc finger protein WIP3
330	TRINITY_DN18478_c0_g1	543	2.27	0.19	25.59	1.92	3.58	Choline-phosphate cytidylyltransferase 1
331	TRINITY_DN29375_c0_g1	1399	3.26	0.10	36.90	1.07	3.53	Equilibrative nucleotide transporter 3
332	TRINITY_DN36693_c0_g1	646	1.98	0.14	24.88	1.57	3.52	2,3-bisphosphoglycerate-independent phosphoglycerate mutase
333	TRINITY_DN8763_c0_g1	552	2.15	0.17	25.27	1.87	3.52	Peroxidase 55
334	TRINITY_DN47298_c0_g1	1046	42.56	1.80	497.05	19.36	3.49	Germin-like protein
335	TRINITY_DN42158_c0_g1	581	1.81	0.14	24.01	1.68	3.47	Probable xyloglucan endotransglucosylase/hydrolase protein 25
336	TRINITY_DN37105_c0_g1	1601	145.96	4.03	1652.51	42.06	3.46	E3 ubiquitin-protein ligase RING1-like
337	TRINITY_DN28289_c0_g2	2141	131.74	2.72	1491.96	28.39	3.46	Phosphoinositide phospholipase C 4
338	TRINITY_DN25628_c0_g2	1133	3.74	0.15	45.47	1.64	3.41	Probable protein phosphatase 2C 30
339	TRINITY_DN34050_c0_g3	763	3.26	0.19	33.55	2.03	3.41	Putative cysteine-rich receptor-like protein kinase 35
340	TRINITY_DN35189_c7_g1	1908	3.20	0.07	33.65	0.72	3.41	Zinc finger A20 and AN1 domain-containing stress-associated protein 5
341	TRINITY_DN30995_c0_g1	810	17.63	0.96	194.62	9.79	3.39	Protein YLS3
342	TRINITY_DN37634_c0_g1	947	15.02	0.70	161.95	6.97	3.38	CASP-like protein 4D1
343	TRINITY_DN11019_c0_g1	737	3.08	0.18	32.97	1.82	3.37	Lanosterol 14-alpha demethylase
344	TRINITY_DN15005_c0_g1	2057	12.29	0.26	127.02	2.52	3.35	Gamma-glutamyltranspeptidase 1
345	TRINITY_DN25840_c0_g1	2708	12.79	0.21	137.03	2.06	3.34	VIN3-like protein 2
346	TRINITY_DN6117_c0_g1	243	1.98	0.36	22.07	3.70	3.34	Riboflavin kinase
347	TRINITY_DN25497_c0_g1	1419	7.81	0.24	84.00	2.41	3.33	Ras-related protein RABD2a
348	TRINITY_DN30338_c0_g1	1079	21.66	0.89	226.62	8.56	3.32	Proline-rich receptor-like protein kinase PERK15
349	TRINITY_DN14985_c0_g1	335	4.10	0.51	40.78	4.21	3.28	Gibberellin 20 oxidase 3
350	TRINITY_DN34387_c1_g2	1526	2.55	0.07	31.28	0.84	3.28	Probable protein phosphatase 2C 55

*logFC: the logarithm to base 2 of fold change (Salt/Control)

**TPM: transcripts per million

Supplementary Table 1. (Cont) Annotation profile of DEG in ice plant seedlings treated with 200 mM NaCl (|FC|>4, FDR<0.001)

No.	Transcript ID	Length	Control reads	Control reads (TPM**)	Salt reads	Salt reads (TPM)	logFC*	Annotation
351	TRINITY_DN50186_c0_g1	415	2.61	0.28	31.46	3.09	3.28	Tobamovirus multiplication protein 1
352	TRINITY_DN46365_c0_g1	247	1.89	0.34	20.97	3.46	3.27	Probable U6 snRNA-associated Sm-like protein LSm3
353	TRINITY_DN34142_c2_g1	1584	2.18	0.06	21.37	0.55	3.27	Dynein regulatory complex subunit 7
354	TRINITY_DN32126_c0_g1	1287	6.32	0.22	60.12	1.90	3.25	Transposon Ty3-G Gag-Pol polyprotein
355	TRINITY_DN4742_c0_g1	915	10.24	0.50	98.21	4.37	3.24	Hevamine-A
356	TRINITY_DN24054_c0_g1	827	89.23	4.77	862.77	42.51	3.23	Major allergen Mal d 1
357	TRINITY_DN8864_c0_g1	866	2.72	0.14	30.30	1.43	3.23	Rust resistance kinase Lr10
358	TRINITY_DN12445_c0_g8	981	3.15	0.14	29.50	1.23	3.23	Peroxisomal membrane protein PEX14
359	TRINITY_DN25161_c0_g1	271	9.55	1.56	95.70	14.39	3.21	Nectarin-1
360	TRINITY_DN17969_c0_g1	997	3.79	0.17	39.04	1.60	3.21	Bifunctional 3-dehydroquinate dehydratase/shikimate dehydrogenase, chloroplastic
361	TRINITY_DN12952_c0_g1	1055	44.03	1.85	415.32	16.04	3.19	Cysteine-rich repeat secretory protein 55
362	TRINITY_DN28236_c0_g2	693	19.00	1.21	179.24	10.54	3.19	WD40 repeat-containing protein HOS15
363	TRINITY_DN24314_c0_g1	583	3.19	0.24	29.33	2.05	3.18	Pyruvate kinase
364	TRINITY_DN31430_c0_g2	3762	43.23	0.51	388.60	4.21	3.13	Structural maintenance of chromosomes protein 1
365	TRINITY_DN32667_c0_g3	656	2.84	0.19	27.75	1.72	3.13	Probable alpha-mannosidase At5g13980
366	TRINITY_DN32426_c0_g4	931	3.15	0.15	27.94	1.22	3.13	Mitochondrial inner membrane protein OXA1-like
367	TRINITY_DN30910_c0_g2	1539	9.97	0.29	90.87	2.41	3.13	Dof zinc finger protein DOF5.3
368	TRINITY_DN30987_c0_g3	1721	22.53	0.58	207.41	4.91	3.12	Protein EI24 homolog
369	TRINITY_DN33379_c4_g1	253	7.51	1.31	72.04	11.60	3.11	Glutathione S-transferase U17
370	TRINITY_DN28570_c0_g1	3061	6.61	0.10	63.19	0.84	3.11	UPF0505 protein
371	TRINITY_DN30695_c2_g1	233	18.35	3.48	160.42	28.05	3.10	Signal peptidase complex subunit 3B
372	TRINITY_DN33810_c1_g2	745	17.31	1.03	150.43	8.23	3.09	BAG-associated GRAM protein 1
373	TRINITY_DN42227_c0_g1	829	615.42	32.83	5391.03	264.98	3.09	Major allergen Mal d 1
374	TRINITY_DN22740_c0_g1	2042	6.98	0.15	62.36	1.24	3.08	Myosin-binding protein 2
375	TRINITY_DN30116_c2_g1	727	23.25	1.41	200.85	11.26	3.08	1-aminocyclopropane-1-carboxylate oxidase homolog 1

*logFC: the logarithm to base 2 of fold change (Salt/Control)

**TPM: transcripts per million

Supplementary Table 1. (Cont) Annotation profile of DEG in ice plant seedlings treated with 200 mM NaCl (|FC|>4, FDR<0.001)

No.	Transcript ID	Length	Control reads	Control reads (TPM**)	Salt reads	Salt reads (TPM)	logFC*	Annotation
376	TRINITY_DN15965_c0_g1	1322	3.33	0.11	26.98	0.83	3.08	Peroxidase 5
377	TRINITY_DN21822_c0_g1	460	2.62	0.25	27.48	2.43	3.08	Cytochrome P450 CYP72A219
378	TRINITY_DN52619_c0_g1	2664	1447.77	24.03	12576.81	192.37	3.08	Pyruvate decarboxylase 1
379	TRINITY_DN31736_c1_g1	1178	15.21	0.57	130.88	4.53	3.08	Transcription initiation factor TFIID subunit 1
380	TRINITY_DN29316_c0_g1	836	14.99	0.79	131.38	6.40	3.08	Cytochrome P450 714A1
381	TRINITY_DN31220_c4_g1	296	8.85	1.32	78.50	10.81	3.06	Glutathione S-transferase U9
382	TRINITY_DN24370_c1_g1	637	3.74	0.26	34.91	2.23	3.05	Peroxidase 57
383	TRINITY_DN35074_c2_g1	596	5.85	0.43	51.95	3.55	3.05	Putative cysteine-rich receptor-like protein kinase 31
384	TRINITY_DN39356_c0_g1	934	14.11	0.67	119.18	5.20	3.04	AAA-ATPase At3g28600
385	TRINITY_DN23907_c0_g2	671	3.27	0.22	25.97	1.58	3.02	DNA polymerase lambda
386	TRINITY_DN31330_c0_g1	1358	5.56	0.18	50.95	1.53	3.02	Protein OSB1, mitochondrial
387	TRINITY_DN53158_c0_g2	3564	32.04	0.40	265.08	3.03	3.00	Nuclear pore complex protein NUP98A
388	TRINITY_DN32346_c0_g2	349	5.04	0.64	41.88	4.89	3.00	SKP1-like protein 1B
389	TRINITY_DN28639_c0_g3	779	11.52	0.65	98.78	5.17	2.99	Protein AE7
390	TRINITY_DN22506_c0_g1	1387	87.01	2.77	708.13	20.80	2.98	BMP-binding endothelial regulator protein
391	TRINITY_DN33959_c0_g1	851.33	11.32	0.60	89.64	3.24	2.98	Benzyl alcohol O-benzoyltransferase
392	TRINITY_DN30173_c0_g1	1903	2.54	0.06	24.53	0.53	2.97	Probable WRKY transcription factor 23
393	TRINITY_DN39897_c0_g1	397	2.97	0.33	25.38	2.61	2.97	Protein KES1
394	TRINITY_DN34002_c0_g2	1314	3.50	0.12	32.73	1.01	2.97	Short-chain type dehydrogenase/reductase
395	TRINITY_DN30838_c0_g1	797	5.61	0.31	49.35	2.52	2.96	RING-H2 finger protein ATL43
396	TRINITY_DN39395_c0_g1	615	5.88	0.42	49.15	3.26	2.96	NDR1/HIN1-like protein 13
397	TRINITY_DN38464_c0_g1	630	8.83	0.62	73.10	4.73	2.96	Cysteine-rich receptor-like protein kinase 10
398	TRINITY_DN35350_c0_g2	2802	27.06	0.43	214.73	3.12	2.95	Probable leucine-rich repeat receptor-like protein kinase At5g49770
399	TRINITY_DN18460_c0_g1	1085	46.13	1.88	363.41	13.65	2.94	22.0 kDa heat shock protein
400	TRINITY_DN50748_c0_g1	448	5.75	0.57	47.52	4.32	2.93	Shikimate O-hydroxycinnamoyltransferase

*logFC: the logarithm to base 2 of fold change (Salt/Control)

**TPM: transcripts per million

Supplementary Table 1. (Cont) Annotation profile of DEG in ice plant seedlings treated with 200 mM NaCl (|FC|>4, FDR<0.001)

No.	Transcript ID	Length	Control reads	Control reads (TPM**)	Salt reads	Salt reads (TPM)	logFC*	Annotation
401	TRINITY_DN34165_c0_g2	2012	2.67	0.06	23.82	0.48	2.91	Transmembrane protein 8A
402	TRINITY_DN26386_c0_g1	762	62.42	3.62	476.83	25.50	2.90	Putative lipid-binding protein AIR1
403	TRINITY_DN53984_c0_g1	842	11.99	0.63	92.03	4.45	2.88	Basic secretory protease
404	TRINITY_DN18358_c0_g1	852	309.19	16.05	2291.12	109.57	2.85	Pathogenesis-related protein PR-4B
405	TRINITY_DN20713_c0_g1	545	6.24	0.51	44.53	3.33	2.84	Cell number regulator 10
406	TRINITY_DN34413_c0_g2	860	9.48	0.49	66.87	3.17	2.84	GPI ethanolamine phosphate transferase 3
407	TRINITY_DN55194_c0_g1	2027	14.56	0.32	111.10	2.23	2.84	Protein NUCLEAR FUSION DEFECTIVE 4
408	TRINITY_DN34324_c1_g1	1434	123.80	3.82	906.52	25.76	2.83	Electron transfer flavoprotein subunit alpha, mitochondrial
409	TRINITY_DN22372_c0_g1	573	4.39	0.34	30.02	2.13	2.83	Protein ACCELERATED CELL DEATH 6
410	TRINITY_DN32499_c0_g2	1118	23.57	0.93	175.21	6.39	2.82	Peroxidase 2
411	TRINITY_DN55224_c0_g1	843	6.14	0.32	44.32	2.14	2.81	BON1-associated protein 2
412	TRINITY_DN35145_c6_g1	984.2	29.87	1.08	214.80	9.52	2.80	Calcineurin B-like protein 7
413	TRINITY_DN26652_c0_g1	791	38.99	2.18	279.37	14.39	2.79	Protein MALE DISCOVERER 2
414	TRINITY_DN25606_c1_g1	921	17.31	0.83	120.62	5.34	2.78	Probable glycosyltransferase At5g25310
415	TRINITY_DN26821_c0_g1	1670	268.22	7.10	1896.32	46.27	2.78	Auxin-responsive protein IAA7
416	TRINITY_DN30407_c0_g4	203	18.50	4.03	127.59	25.61	2.78	Chlorophyll a-b binding protein 40, chloroplastic
417	TRINITY_DN8198_c0_g1	358	4.13	0.51	29.10	3.31	2.78	Mitochondrial pyruvate carrier 4
418	TRINITY_DN35023_c1_g1	4399	3.51	0.04	29.37	0.27	2.78	Mediator of RNA polymerase II transcription subunit 33A
419	TRINITY_DN32055_c0_g4	2459	3.70	0.07	28.96	0.48	2.78	E3 ubiquitin-protein ligase RFWD3
420	TRINITY_DN7335_c0_g1	745	3.60	0.21	29.14	1.59	2.78	E3 ubiquitin-protein ligase ATL6
421	TRINITY_DN36850_c0_g1	306	6.77	0.98	50.22	6.69	2.77	Bifunctional purple acid phosphatase 26
422	TRINITY_DN30956_c1_g4	2105	8.97	0.19	64.31	1.24	2.77	Acyl-coenzyme A oxidase 2, peroxisomal
423	TRINITY_DN26776_c0_g1	1811	9.74	0.24	70.87	1.59	2.77	TPR repeat-containing thioredoxin TTL1
424	TRINITY_DN35079_c7_g1	1039	10.82	0.46	77.96	3.06	2.77	Ethylene-responsive transcription factor ERF053
425	TRINITY_DN34622_c1_g1	593	14.00	1.04	99.07	6.81	2.77	Putative DNA (cytosine-5)-methyltransferase CMT1

*logFC: the logarithm to base 2 of fold change (Salt/Control)

**TPM: transcripts per million

Supplementary Table 1. (Cont) Annotation profile of DEG in ice plant seedlings treated with 200 mM NaCl (|FC|>4, FDR<0.001)

No.	Transcript ID	Length	Control reads	Control reads (TPM**)	Salt reads	Salt reads (TPM)	logFC*	Annotation
426	TRINITY_DN49060_c0_g6	1514	34.09	1.00	237.64	6.40	2.76	Chitotriosidase-1
427	TRINITY_DN22478_c0_g1	744.33	9.88	0.58	70.18	3.80	2.75	Acidic mammalian chitinase
428	TRINITY_DN24111_c0_g2	1062	7.22	0.30	48.93	1.84	2.74	dCTP pyrophosphatase 1
429	TRINITY_DN29024_c0_g1	1423	6.86	0.21	49.19	1.41	2.74	Probable indole-3-pyruvate monooxygenase YUCCA3
430	TRINITY_DN28289_c0_g1	2555	24.76	0.43	172.83	2.76	2.74	Phosphoinositide phospholipase C 6
431	TRINITY_DN4073_c0_g1	1997	6.50	0.14	41.74	0.85	2.74	Cytochrome P450 76AD1
432	TRINITY_DN4353_c0_g2	546	10.93	0.89	76.18	5.69	2.73	Cysteine proteinase inhibitor 5
433	TRINITY_DN31637_c0_g1	1523.2	39.32	1.10	265.33	6.12	2.72	Protein NRT1/ PTR FAMILY 5.5
434	TRINITY_DN27256_c0_g2	1346	9.50	0.31	67.96	2.06	2.71	Protein YIF1A
435	TRINITY_DN22807_c0_g4	1883	82.61	1.94	557.54	12.06	2.71	Actin-related protein 4
436	TRINITY_DN12952_c0_g2	1054	357.26	14.99	2374.44	91.79	2.69	Cysteine-rich repeat secretory protein 55
437	TRINITY_DN26555_c0_g2	2272	19.33	0.38	127.33	2.28	2.69	Nucleolar complex protein 4 homolog B
438	TRINITY_DN13009_c0_g3	1197	50.51	1.87	336.88	11.47	2.68	Probable glutathione peroxidase 4
439	TRINITY_DN15201_c0_g1	752	26.82	1.58	177.52	9.62	2.67	Phytosulfokines 5
440	TRINITY_DN49471_c0_g1	1041	13.64	0.58	91.79	3.59	2.66	Transcription factor MYB44
441	TRINITY_DN28889_c0_g1	1418	50.85	1.59	330.75	9.50	2.65	12-oxophytodienoate reductase 3
442	TRINITY_DN47275_c0_g4	1120	7.20	0.28	46.41	1.69	2.65	Zinc finger protein ZAT10
443	TRINITY_DN48219_c0_g1	1366	19.89	0.64	130.42	3.89	2.65	Polygalacturonase inhibitor
444	TRINITY_DN13755_c0_g3	1531	23.67	0.68	154.86	4.12	2.64	Protein ORANGE-LIKE, chloroplastic
445	TRINITY_DN35295_c0_g1	538	65.94	5.42	425.12	32.20	2.64	Squalene synthase 1
446	TRINITY_DN8436_c1_g1	668	5.82	0.39	38.69	2.36	2.63	Potassium transporter 5
447	TRINITY_DN2345_c0_g1	456	14.74	1.43	95.50	8.53	2.63	Acidic endochitinase SP2
448	TRINITY_DN33064_c1_g2	1734	15.75	0.40	102.03	2.40	2.62	Leucine-rich repeat extensin-like protein 4
449	TRINITY_DN30449_c0_g4	2030	27.44	0.60	170.97	3.43	2.62	Alcohol dehydrogenase-like 7
450	TRINITY_DN33064_c1_g1	1989	65.70	1.46	416.54	8.53	2.62	Leucine-rich repeat extensin-like protein 4

*logFC: the logarithm to base 2 of fold change (Salt/Control)

**TPM: transcripts per million

Supplementary Table 1. (Cont) Annotation profile of DEG in ice plant seedlings treated with 200 mM NaCl (|FC|>4, FDR<0.001)

No.	Transcript ID	Length	Control reads	Control reads (TPM**)	Salt reads	Salt reads (TPM)	logFC*	Annotation
451	TRINITY_DN53014_c0_g1	730	5.26	0.32	31.96	1.78	2.61	Putative cysteine-rich repeat secretory protein 7
452	TRINITY_DN33489_c4_g3	2834	183.19	2.86	1131.24	16.27	2.59	Cytochrome b559 subunit alpha
453	TRINITY_DN34238_c0_g1	1231	46.45	1.67	280.02	9.27	2.56	Embryo-specific protein ATS3B
454	TRINITY_DN36994_c0_g1	465	48.77	4.64	298.01	26.11	2.56	Germin-like protein 5-1
455	TRINITY_DN19927_c0_g1	1188	6.42	0.24	36.80	1.26	2.56	Putative ribonuclease H protein At1g65750
456	TRINITY_DN22359_c0_g2	1383	6.03	0.19	36.82	1.08	2.56	DNA polymerase zeta processivity subunit
457	TRINITY_DN33386_c3_g1	621	23.95	1.71	145.71	9.56	2.56	UDP-glycosyltransferase 73B3
458	TRINITY_DN34115_c1_g1	1415	160.22	5.01	969.04	27.90	2.56	Ribosomal RNA-processing protein 14
459	TRINITY_DN33012_c0_g1	610	9.82	0.71	60.99	4.07	2.55	Choline monooxygenase, chloroplastic
460	TRINITY_DN12345_c0_g1	783	19.33	1.09	115.01	5.99	2.55	NDR1/HIN1-like protein 2
461	TRINITY_DN54018_c0_g1	1665	36.65	0.97	223.05	5.46	2.55	Sugar transporter ERD6-like 16
462	TRINITY_DN1619_c0_g1	1365	22.52	0.73	137.63	4.11	2.54	Cadmium/zinc-transporting ATPase HMA2
463	TRINITY_DN33489_c4_g2	2836	199.55	3.11	1193.47	17.15	2.53	Cytochrome b559 subunit alpha
464	TRINITY_DN31446_c0_g2	4240	167.98	1.75	1002.02	9.63	2.53	NAC domain-containing protein 89
465	TRINITY_DN24564_c0_g1	2118	8.91	0.19	53.79	1.03	2.53	Receptor-like serine/threonine-protein kinase At4g25390
466	TRINITY_DN26328_c0_g1	1055.2	206.86	8.50	1227.72	44.56	2.53	Vicilin-like seed storage protein At2g18540
467	TRINITY_DN28308_c0_g1	2049	620.74	13.40	3647.01	72.53	2.51	3-ketoacyl-CoA synthase 5
468	TRINITY_DN24202_c0_g1	1416	245.99	7.68	1439.60	41.43	2.51	Cysteine-rich repeat secretory protein 55
469	TRINITY_DN24449_c0_g1	2398.3	560.10	10.25	3232.63	54.76	2.49	Probable sulfate transporter 3.5
470	TRINITY_DN26280_c0_g1	826	58.81	3.15	340.92	16.82	2.49	Major allergen Mal d 1
471	TRINITY_DN11888_c0_g2	1858	10.93	0.26	64.46	1.41	2.49	Polyvinylalcohol dehydrogenase
472	TRINITY_DN33450_c1_g3	1424	8.55	0.27	51.85	1.48	2.47	Cytochrome P450 76A2
473	TRINITY_DN35014_c1_g4	723	8.55	0.52	52.34	2.95	2.47	Wall-associated receptor kinase-like 22
474	TRINITY_DN33169_c0_g2	1150	5.08	0.20	28.63	1.01	2.47	Uncharacterized WD repeat-containing protein C3H5.08c
475	TRINITY_DN13187_c0_g1	632	256.82	17.97	1458.62	94.04	2.46	Expansin-A2

*logFC: the logarithm to base 2 of fold change (Salt/Control)

**TPM: transcripts per million

Supplementary Table 1. (Cont) Annotation profile of DEG in ice plant seedlings treated with 200 mM NaCl (|FC|>4, FDR<0.001)

No.	Transcript ID	Length	Control reads	Control reads (TPM**)	Salt reads	Salt reads (TPM)	logFC*	Annotation
476	TRINITY_DN30396_c0_g2	1325	17.18	0.57	97.34	2.99	2.46	Probable E3 ubiquitin-protein ligase ARI2
477	TRINITY_DN29569_c0_g2	2096	9.76	0.21	57.46	1.12	2.46	Serine/threonine-protein kinase 38-like
478	TRINITY_DN42622_c0_g1	807	129.37	7.09	727.54	36.74	2.45	Oleosin 16 kDa
479	TRINITY_DN47012_c0_g4	1304	11.56	0.39	68.31	2.13	2.45	ER membrane protein complex subunit 4
480	TRINITY_DN48379_c0_g4	1637	18.65	0.50	106.80	2.66	2.44	WAT1-related protein At1g09380
481	TRINITY_DN27447_c0_g3	2806	98.54	1.55	554.02	8.05	2.44	Glycine-rich domain-containing protein 2
482	TRINITY_DN21713_c0_g1	1008	28.38	1.24	157.47	6.37	2.44	Probable carboxylesterase 8
483	TRINITY_DN25551_c0_g1	3795	27.57	0.32	157.46	1.69	2.44	Exosome complex exonuclease RRP44 homolog A
484	TRINITY_DN41471_c0_g4	1258	71.18	2.50	396.54	12.84	2.44	Pre-mRNA cleavage factor Im 25 kDa subunit 2
485	TRINITY_DN34738_c0_g2	2397	6.37	0.12	34.06	0.58	2.44	E3 ubiquitin-protein ligase SHPRH
486	TRINITY_DN43311_c0_g1	1372	15.29	0.49	83.85	2.49	2.43	Probable carboxylesterase 17
487	TRINITY_DN15965_c0_g3	1190	30.52	1.13	172.97	5.92	2.43	Peroxidase 5
488	TRINITY_DN26460_c0_g1	3163	26.21	0.37	144.89	1.87	2.43	Type II inositol polyphosphate 5-phosphatase 15
489	TRINITY_DN27048_c0_g1	2682	36.31	0.60	200.08	3.04	2.43	Transcriptional corepressor LEUNIG
490	TRINITY_DN48352_c0_g1	1090	158.47	6.43	874.13	32.68	2.43	Heavy metal-associated isoprenylated plant protein 47
491	TRINITY_DN28735_c0_g1	2123	145.83	3.04	807.04	15.49	2.42	Heavy metal-associated isoprenylated plant protein 7
492	TRINITY_DN41676_c0_g2	1987	50.96	1.13	281.60	5.77	2.42	Protein AUXIN RESPONSE 4
493	TRINITY_DN25472_c0_g2	1043	39.95	1.69	221.17	8.64	2.42	Copper transporter 6
494	TRINITY_DN23211_c0_g2	1960	261.77	5.91	1442.26	29.98	2.42	Sugar transporter ERD6-like 16
495	TRINITY_DN33057_c0_g2	739	48.51	2.90	269.62	14.87	2.42	Mediator of RNA polymerase II transcription subunit 14
496	TRINITY_DN35425_c4_g9	1626	11.19	0.30	61.27	1.54	2.42	Transcription activator GLK1
497	TRINITY_DN33877_c3_g2	950	116.78	5.44	638.24	27.38	2.40	Transcription factor SCREAM2
498	TRINITY_DN32892_c0_g1	323.24	51.15	6.91	278.34	31.88	2.40	IN2-2 protein
499	TRINITY_DN54078_c0_g1	1156	22.10	0.85	120.49	4.25	2.40	Basic endochitinase
500	TRINITY_DN35359_c6_g3	2442.7	218.24	1.88	1182.48	11.77	2.40	Pyrogallol hydroxytransferase small subunit

*logFC: the logarithm to base 2 of fold change (Salt/Control)

**TPM: transcripts per million

Supplementary Table 1. (Cont) Annotation profile of DEG in ice plant seedlings treated with 200 mM NaCl (|FC|>4, FDR<0.001)

No.	Transcript ID	Length	Control reads	Control reads (TPM**)	Salt reads	Salt reads (TPM)	logFC*	Annotation
501	TRINITY_DN28014_c0_g2	1877	15.16	0.36	81.06	1.76	2.38	Putative aminoacrylate hydrolase RutD
502	TRINITY_DN11092_c0_g1	1659	14.54	0.39	81.26	2.00	2.38	Glutamate receptor 2.5
503	TRINITY_DN35387_c0_g3	1540.9	71.91	2.34	385.50	12.88	2.38	Delta(12)-fatty-acid desaturase FAD2
504	TRINITY_DN50330_c0_g1	524	7.05	0.59	37.73	2.93	2.38	Probable E3 ubiquitin-protein ligase RHC2A
505	TRINITY_DN4118_c0_g1	1395	21.75	0.69	116.98	3.42	2.36	Ankyrin repeat-containing protein BDA1
506	TRINITY_DN26185_c0_g3	2505	18.66	0.33	101.00	1.64	2.36	Probable inactive purple acid phosphatase 2
507	TRINITY_DN26169_c0_g2	1736	30.27	0.77	159.07	3.73	2.36	Polygalacturonase At1g48100
508	TRINITY_DN35425_c4_g8	1626	40.96	1.11	217.26	5.44	2.36	Transcription activator GLK1
509	TRINITY_DN32880_c1_g1	1535	8.99	0.26	48.28	1.28	2.36	Protein PHOTOPERIOD-INDEPENDENT EARLY FLOWERING 1
510	TRINITY_DN5912_c0_g1	605	5.93	0.43	32.40	2.18	2.35	CASP-like protein 4B3
511	TRINITY_DN42018_c0_g1	900	64.11	3.15	334.28	15.13	2.34	Protein SRC2 homolog
512	TRINITY_DN28983_c0_g2	824	8.00	0.43	42.24	2.09	2.33	Probable zinc metallopeptidase EGY3, chloroplastic
513	TRINITY_DN33440_c1_g2	1650	8.33	0.22	41.92	1.04	2.33	Zinc finger CCCH domain-containing protein 12
514	TRINITY_DN28241_c0_g2	1417	50.41	1.57	259.38	7.46	2.33	Two-component response regulator ARR9
515	TRINITY_DN29676_c0_g5	1355	15.73	0.51	83.44	2.51	2.33	Thioredoxin M4, chloroplastic
516	TRINITY_DN25005_c0_g1	1274	10.39	0.36	51.62	1.65	2.32	Hyoscyamine 6-dioxygenase
517	TRINITY_DN51114_c0_g1	579	17.46	1.33	87.65	6.17	2.32	Cytochrome P450 76C3
518	TRINITY_DN35425_c4_g2	1625	11.69	no	61.56	no	2.32	Transcription activator GLK1
519	TRINITY_DN47309_c0_g1	1033	13.67	0.59	71.96	2.84	2.31	Protein SAR DEFICIENT 1
520	TRINITY_DN21137_c0_g1	788	6.86	0.38	36.43	1.88	2.30	Tetraspanin-9
521	TRINITY_DN22315_c0_g2	1282	9.90	0.34	50.63	1.61	2.30	Tetraspanin-19
522	TRINITY_DN31596_c1_g2	1565	16.45	0.46	80.77	2.10	2.29	Abscisic acid 8'-hydroxylase 2
523	TRINITY_DN30202_c0_g1	1051	17.96	0.76	91.32	3.54	2.29	Exonuclease DPD1, chloroplastic/mitochondrial
524	TRINITY_DN29528_c0_g1	4157	18.75	0.20	96.38	0.94	2.29	RRP12-like protein
525	TRINITY_DN49060_c0_g2	1514	554.40	16.19	2776.74	74.73	2.28	Chitotriosidase-1

*logFC: the logarithm to base 2 of fold change (Salt/Control)

**TPM: transcripts per million

Supplementary Table 1. (Cont) Annotation profile of DEG in ice plant seedlings treated with 200 mM NaCl (|FC|>4, FDR<0.001)

No.	Transcript ID	Length	Control reads	Control reads (TPM**)	Salt reads	Salt reads (TPM)	logFC*	Annotation
526	TRINITY_DN31695_c0_g1	1836.3	65.22	1.28	326.28	5.28	2.28	Putative phospholipid-transporting ATPase 9
527	TRINITY_DN31202_c0_g1	2119	257.56	5.37	1290.27	24.81	2.28	Homeobox-leucine zipper protein ATHB-16
528	TRINITY_DN11388_c0_g2	873	7.72	0.39	39.58	1.85	2.26	Protein MIZU-KUSSEI 1
529	TRINITY_DN52928_c0_g1	1704	662.79	17.20	3243.66	77.56	2.25	Basic 7S globulin
530	TRINITY_DN41616_c1_g1	1352	231.43	7.57	1124.57	33.89	2.24	Strigolactone esterase D14
531	TRINITY_DN22916_c0_g1	989	10.94	0.49	53.99	2.22	2.24	Epidermis-specific secreted glycoprotein EP1
532	TRINITY_DN31550_c0_g4	1618.7	3249.73	95.30	15775.11	476.36	2.24	Alcohol dehydrogenase 1
533	TRINITY_DN32524_c0_g2	1048	14.57	0.61	72.87	2.83	2.23	Formamidopyrimidine-DNA glycosylase
534	TRINITY_DN25259_c0_g2	1378	11.07	0.36	53.02	1.57	2.21	Capsid protein
535	TRINITY_DN29595_c0_g1	5428	229.73	1.92	1097.79	8.49	2.21	7-hydroxymethyl chlorophyll a reductase, chloroplastic
536	TRINITY_DN21508_c0_g1	446	15.34	1.52	71.76	6.56	2.21	Probable transcription factor KAN2
537	TRINITY_DN29199_c0_g2	2063	724.65	15.53	3457.04	68.28	2.21	Cytochrome P450 94A2
538	TRINITY_DN24629_c0_g1	1916	30.84	0.71	148.26	3.15	2.21	Suppressor of glycerol defect protein 1
539	TRINITY_DN22973_c0_g1	565.66	13.61	0.82	67.37	3.03	2.21	Phototropin-2
540	TRINITY_DN23593_c0_g2	1084	17.11	0.70	80.80	3.04	2.20	Transmembrane protein 208 homolog
541	TRINITY_DN35142_c3_g2	1885	383.31	8.99	1815.33	39.24	2.20	Zinc finger protein CONSTANS-LIKE 16
542	TRINITY_DN34948_c2_g2	752	13.24	0.78	62.01	3.36	2.20	Putative ribonuclease H protein At1g65750
543	TRINITY_DN30396_c0_g1	2202	12.69	0.25	62.18	1.15	2.20	Probable E3 ubiquitin-protein ligase ARI2
544	TRINITY_DN30283_c0_g1	2325	1192.81	22.69	5633.34	98.73	2.20	Methylcrotonoyl-CoA carboxylase beta chain, mitochondrial
545	TRINITY_DN28473_c0_g1	1590	15.33	0.43	71.01	1.82	2.19	Protein PIN-LIKES 1
546	TRINITY_DN41534_c0_g1	1435	73.10	2.25	342.67	9.73	2.19	Cysteine proteinase inhibitor 10
547	TRINITY_DN35590_c0_g1	1570	388.94	10.95	1818.62	47.20	2.18	BURP domain protein RD22
548	TRINITY_DN48286_c0_g1	1299	31.69	1.08	150.16	4.71	2.18	Desiccation-related protein PCC13-62
549	TRINITY_DN9054_c0_g1	1160	61.98	2.36	288.93	10.15	2.18	Cysteine-rich repeat secretory protein 1
550	TRINITY_DN33447_c0_g1	624.6	37.61	2.57	176.54	10.79	2.18	Transcription factor MYB62

*logFC: the logarithm to base 2 of fold change (Salt/Control)

**TPM: transcripts per million

Supplementary Table 1. (Cont) Annotation profile of DEG in ice plant seedlings treated with 200 mM NaCl (|FC|>4, FDR<0.001)

No.	Transcript ID	Length	Control reads	Control reads (TPM**)	Salt reads	Salt reads (TPM)	logFC*	Annotation
551	TRINITY_DN47131_c0_g1	885	21.45	1.07	97.88	4.51	2.17	Protein DETOXIFICATION 54
552	TRINITY_DN36406_c0_g1	1894	478.99	11.18	2208.21	47.51	2.16	Aldehyde dehydrogenase family 2 member B7, mitochondrial
553	TRINITY_DN15959_c0_g1	808	201.78	11.04	931.08	46.95	2.16	Kunitz trypsin inhibitor 2
554	TRINITY_DN34715_c2_g1	1683	11.48	0.30	51.35	1.24	2.16	Probable RNA-binding protein ARP1
555	TRINITY_DN30272_c0_g1	2445.8	100.31	1.82	457.23	6.60	2.15	ATP-dependent zinc metalloprotease FTSH 6, chloroplastic
556	TRINITY_DN33057_c0_g3	8644	151.64	0.78	693.87	3.27	2.15	Mediator of RNA polymerase II transcription subunit 14
557	TRINITY_DN20600_c0_g1	595	10.34	0.77	46.27	3.17	2.15	Petal death protein
558	TRINITY_DN9096_c0_g1	1215	19.39	0.71	87.39	2.93	2.15	Putative pectinesterase 11
559	TRINITY_DN33210_c2_g1	1320	220.01	7.37	1000.60	30.89	2.14	Peroxidase 4
560	TRINITY_DN30284_c0_g3	701	12.21	0.77	55.37	3.22	2.14	Protein BRASSINOSTEROID INSENSITIVE 1
561	TRINITY_DN17012_c0_g1	2232	599.59	11.88	2727.93	49.80	2.14	Cytochrome P450 CYP73A100
562	TRINITY_DN28702_c0_g1	1423	288.46	8.96	1306.69	37.42	2.14	Peroxidase 12
563	TRINITY_DN30920_c0_g1	1037	8.95	0.38	40.75	1.60	2.13	Basic 7S globulin
564	TRINITY_DN27088_c0_g2	1112	13.21	0.53	59.08	2.17	2.13	Protein ACCELERATED CELL DEATH 6
565	TRINITY_DN34017_c2_g1	3481	108.19	1.37	485.82	5.69	2.13	Pleiotropic drug resistance protein 1
566	TRINITY_DN31550_c0_g2	1165	93.87	3.56	423.37	14.81	2.13	Alcohol dehydrogenase 2
567	TRINITY_DN33873_c0_g2	1867	55.92	1.32	251.09	5.48	2.12	Geraniol 8-hydroxylase
568	TRINITY_DN26723_c0_g1	1068	19.36	0.80	85.20	3.25	2.11	BURP domain protein RD22
569	TRINITY_DN39906_c0_g1	633	8.38	0.59	35.56	2.29	2.11	Probable polyol transporter 4
570	TRINITY_DN32585_c0_g1	495	14.73	1.32	66.96	5.51	2.11	Geranylgeranyl transferase type-1 subunit beta
571	TRINITY_DN35425_c4_g6	1646	59.47	1.60	259.61	6.43	2.10	Transcription activator GLK1
572	TRINITY_DN11691_c0_g1	683	14.20	0.92	61.68	3.68	2.10	Protein PLANT CADMIUM RESISTANCE 12
573	TRINITY_DN13778_c0_g2	310	8.93	1.27	39.54	5.20	2.10	Vacuolar protein sorting-associated protein 36
574	TRINITY_DN30272_c0_g2	1200	97.14	3.58	427.12	14.50	2.10	ATP-dependent zinc metalloprotease FTSH 6, chloroplastic
575	TRINITY_DN3005_c0_g1	1093	92.35	3.74	405.30	15.11	2.10	Cysteine-rich repeat secretory protein 38

*logFC: the logarithm to base 2 of fold change (Salt/Control)

**TPM: transcripts per million

Supplementary Table 1. (Cont) Annotation profile of DEG in ice plant seedlings treated with 200 mM NaCl (|FC|>4, FDR<0.001)

No.	Transcript ID	Length	Control reads	Control reads (TPM**)	Salt reads	Salt reads (TPM)	logFC*	Annotation
576	TRINITY_DN30117_c0_g1	526	35.55	2.99	158.11	12.25	2.09	Pectin acetylesterase 3
577	TRINITY_DN31884_c1_g1	1322	114.65	3.83	503.29	15.51	2.09	EID1-like F-box protein 3
578	TRINITY_DN32210_c0_g5	1347	23.99	0.79	104.53	3.16	2.08	INO80 complex subunit C
579	TRINITY_DN21154_c0_g1	388	36.07	4.11	156.78	16.46	2.08	Acidic endochitinase SP2
580	TRINITY_DN5321_c0_g1	675	198.77	13.02	853.27	51.51	2.06	Defensin-like protein 1
581	TRINITY_DN15052_c0_g1	1362	83.15	2.70	355.37	10.63	2.05	Probable inactive leucine-rich repeat receptor kinase XIAO
582	TRINITY_DN23333_c0_g2	1499	39.05	1.15	167.28	4.55	2.05	Polygalacturonase
583	TRINITY_DN26106_c0_g3	2881	294.73	4.52	1259.63	17.82	2.05	Monosaccharide-sensing protein 2
584	TRINITY_DN4187_c0_g1	770	33.43	1.92	140.67	7.44	2.05	Probable disease resistance protein At4g33300
585	TRINITY_DN44604_c0_g3	1992	21.99	0.49	93.73	1.92	2.05	Protein DETOXIFICATION 49
586	TRINITY_DN23167_c0_g1	1124	155.41	6.11	656.23	23.79	2.04	Xyloglucan endotransglucosylase/hydrolase protein 22
587	TRINITY_DN14965_c0_g1	1582	13.02	0.36	54.96	1.42	2.03	Senescence/dehydration-associated protein At4g35985, chloroplastic
588	TRINITY_DN35295_c2_g8	563	28.10	2.21	117.51	8.51	2.03	Squalene synthase 1
589	TRINITY_DN6767_c0_g1	944	671.80	31.47	2818.67	121.67	2.03	pEARLI1-like lipid transfer protein 3
590	TRINITY_DN33129_c0_g2	890	214.07	10.64	897.36	41.08	2.03	Pathogenesis-related protein STH-21
591	TRINITY_DN33129_c0_g1	859.68	150.58	7.83	632.83	32.53	2.03	Pathogenesis-related protein STH-21
592	TRINITY_DN47342_c0_g1	736	224.66	13.50	942.54	52.18	2.03	Defensin-like protein 19
593	TRINITY_DN28726_c0_g2	549	120.19	9.68	501.60	37.23	2.02	2-Cys peroxiredoxin BAS1, chloroplastic
594	TRINITY_DN33210_c0_g1	726	115.60	7.04	485.01	27.22	2.02	Peroxidase 4
595	TRINITY_DN32364_c1_g2	1199	59.60	2.20	250.17	8.50	2.02	Enoyl-CoA delta isomerase 2, peroxisomal
596	TRINITY_DN35406_c0_g1	494	11.26	1.01	45.79	3.78	2.01	Twinkle homolog protein, chloroplastic/mitochondrial
597	TRINITY_DN54797_c0_g2	1729	39.36	1.01	162.04	3.82	2.01	Ribosomal L1 domain-containing protein 1
598	TRINITY_DN33489_c4_g1	2835	1713.79	26.73	7103.04	102.09	2.01	Cytochrome b559 subunit alpha
599	TRINITY_DN24942_c0_g1	487	13.46	1.22	53.70	4.49	2.00	Expansin-A8

*logFC: the logarithm to base 2 of fold change (Salt/Control)

**TPM: transcripts per million

Supplementary Table 1. Annotation profile of DEG in ice plant seedlings treated with 200 mM NaCl (|FC|>4, FDR<0.001)

No.	Transcript ID	Length	Control reads	Control reads (TPM**)	Salt reads	Salt reads (TPM)	logFC*	Annotation
1	TRINITY_DN31919_c0_g2	1538	430.11	12.37	0.01	0.00	-11.77	Protein ASPARTIC PROTEASE IN GUARD CELL 1
2	TRINITY_DN6557_c0_g4	1550	412.80	11.78	0.25	0.01	-11.71	Auxin-responsive protein IAA16
3	TRINITY_DN6557_c0_g1	1556	406.07	11.54	0.21	0.01	-11.69	Auxin-responsive protein IAA16
4	TRINITY_DN17177_c0_g1	813	392.12	21.33	0.01	0.00	-11.64	ADP-ribosylation factor-like protein 8a
5	TRINITY_DN6557_c0_g2	1547	345.37	9.87	0.27	0.01	-11.45	Auxin-responsive protein IAA16
6	TRINITY_DN34937_c0_g1	1617.96	334.41	8.79	0.00	0.00	-11.40	Cytochrome c oxidase subunit 1
7	TRINITY_DN7126_c0_g1	594	326.42	24.30	0.00	0.00	-11.37	Oleosin 16.4 kDa
8	TRINITY_DN34372_c0_g1	957.62	267.96	12.53	0.00	0.00	-11.09	60S ribosomal protein L12
9	TRINITY_DN29194_c0_g1	778	248.61	14.13	0.00	0.00	-10.98	Cytochrome c oxidase subunit 3
10	TRINITY_DN53398_c0_g1	627	242.83	17.13	0.02	0.00	-10.95	MFP1 attachment factor 1
11	TRINITY_DN35169_c0_g1	1359.99	243.38	7.09	0.00	0.00	-10.95	Collagen alpha-1(I) chain
12	TRINITY_DN4455_c0_g1	2069	240.28	5.14	0.01	0.00	-10.93	Scarecrow-like protein 3
13	TRINITY_DN13046_c0_g1	1149	237.44	9.14	0.00	0.00	-10.91	UDP-galactose/UDP-glucose transporter 5
14	TRINITY_DN53398_c0_g3	626	208.89	14.76	0.01	0.00	-10.73	MFP1 attachment factor 1
15	TRINITY_DN33122_c0_g1	2098	141.98	2.99	0.00	0.00	-10.17	Rop guanine nucleotide exchange factor 7
16	TRINITY_DN28146_c0_g1	1151	135.50	5.21	0.00	0.00	-10.11	Cationic amino acid transporter 7, chloroplastic
17	TRINITY_DN31091_c0_g2	1830	133.76	3.23	0.00	0.00	-10.09	RHOMBOID-like protein 13
18	TRINITY_DN6277_c0_g3	826	125.76	6.73	0.00	0.00	-10.00	ATP synthase subunit a
19	TRINITY_DN30794_c0_g2	667	121.70	8.07	0.03	0.00	-9.95	ABC transporter B family member 29, chloroplastic
20	TRINITY_DN30954_c0_g1	693	118.21	7.54	0.35	0.02	-9.90	Polygalacturonase non-catalytic subunit AroGP2
21	TRINITY_DN29518_c0_g1	1005	114.45	5.04	0.00	0.00	-9.86	Metal tolerance protein 12
22	TRINITY_DN35126_c0_g2	1658.44	111.01	2.95	0.00	0.00	-9.82	Collagen alpha-2(I) chain
23	TRINITY_DN54200_c0_g2	569	108.68	8.45	0.00	0.00	-9.79	Probable isoaspartyl peptidase/L-asparaginase 2
24	TRINITY_DN55647_c0_g1	286	101.52	15.70	0.00	0.00	-9.69	Chlorophyll a-b binding protein 1, chloroplastic
25	TRINITY_DN30404_c0_g2	1472	99.72	3.00	0.00	0.00	-9.67	Growth-regulating factor 10

*logFC: the logarithm to base 2 of fold change (Salt/Control)

**TPM: transcripts per million

Supplementary Table 1. (Cont) Annotation profile of DEG in ice plant seedlings treated with 200 mM NaCl (|FC|>4, FDR<0.001)

No.	Transcript ID	Length	Control reads	Control reads (TPM**)	Salt reads	Salt reads (TPM)	logFC*	Annotation
26	TRINITY_DN31990_c0_g1	702	95.06	5.99	0.00	0.00	-9.59	Keratin, type I cytoskeletal 50 kDa
27	TRINITY_DN27843_c0_g2	1369	92.97	3.00	0.01	0.00	-9.56	Oxygen-independent coproporphyrinogen-III oxidase-like protein sll1917
28	TRINITY_DN14846_c0_g2	526	92.87	7.81	0.00	0.00	-9.56	Rhomboid-like protein 11, chloroplastic
29	TRINITY_DN29642_c0_g1	707	90.06	5.63	0.00	0.00	-9.51	Transcription factor MYB3R-3
30	TRINITY_DN28239_c0_g1	1079	83.47	3.42	0.00	0.00	-9.40	1-aminocyclopropane-1-carboxylate synthase 7
31	TRINITY_DN23004_c0_g1	363	81.86	9.97	0.00	0.00	-9.38	SPX domain-containing membrane protein At4g11810
32	TRINITY_DN30900_c0_g1	331	80.62	10.77	0.00	0.00	-9.36	60S ribosomal protein L37
33	TRINITY_DN39940_c0_g1	280	78.23	12.35	0.00	0.00	-9.31	Ribulose bisphosphate carboxylase small chain 1A, chloroplastic
34	TRINITY_DN26810_c0_g1	1029	78.08	3.36	0.01	0.00	-9.31	Pentatricopeptide repeat-containing protein At2g21090
35	TRINITY_DN33801_c0_g1	324	77.40	7.67	0.00	0.00	-9.29	40S ribosomal protein S24
36	TRINITY_DN33170_c0_g1	270.61	74.55	6.84	0.00	0.00	-9.25	60S ribosomal protein L32
37	TRINITY_DN31246_c0_g1	2023	74.24	1.62	0.00	0.00	-9.23	Metal tolerance protein C4
38	TRINITY_DN43628_c0_g1	317	73.67	10.28	0.00	0.00	-9.23	60S ribosomal protein L29
39	TRINITY_DN33114_c0_g2	799	72.49	4.01	0.00	0.00	-9.19	Protein PALE CRESS, chloroplastic
40	TRINITY_DN33745_c0_g7	560	72.26	5.71	0.00	0.00	-9.19	Retrovirus-related Pol polyprotein from transposon TNT 1-94
41	TRINITY_DN25875_c0_g1	1060	70.90	2.96	0.00	0.00	-9.17	Pro-Pol polyprotein
42	TRINITY_DN24328_c0_g1	534	68.74	5.69	0.00	0.00	-9.13	40S ribosomal protein S18
43	TRINITY_DN30840_c0_g2	518	66.52	5.68	0.00	0.00	-9.09	Translationally-controlled tumor protein homolog
44	TRINITY_DN30122_c0_g1	639.93	66.43	4.69	0.00	0.00	-9.07	Actin, muscle
45	TRINITY_DN35338_c5_g2	271	64.99	10.60	0.00	0.00	-9.05	Probable 3-hydroxyisobutyryl-CoA hydrolase 2
46	TRINITY_DN13184_c0_g3	745	60.55	3.59	0.00	0.00	-8.95	DNA polymerase delta subunit 4
47	TRINITY_DN36649_c0_g2	2648	57.55	0.96	0.00	0.00	-8.88	Putative pentatricopeptide repeat-containing protein At1g64310
48	TRINITY_DN29045_c0_g2	642	58.50	4.03	0.34	0.02	-8.88	40S ribosomal protein S26E
49	TRINITY_DN33410_c0_g1	692.04	57.87	3.69	0.00	0.00	-8.88	SPARC
50	TRINITY_DN41470_c0_g1	678	56.90	3.71	0.00	0.00	-8.86	Mitochondrial import inner membrane translocase subunit TIM8

*logFC: the logarithm to base 2 of fold change (Salt/Control)

**TPM: transcripts per million

Supplementary Table 1. (Cont) Annotation profile of DEG in ice plant seedlings treated with 200 mM NaCl (|FC|>4, FDR<0.001)

No.	Transcript ID	Length	Control reads	Control reads (TPM**)	Salt reads	Salt reads (TPM)	logFC*	Annotation
51	TRINITY_DN33572_c0_g1	424.02	56.15	5.91	0.00	0.00	-8.83	40S ribosomal protein S25
52	TRINITY_DN32238_c0_g1	395	55.86	6.25	0.00	0.00	-8.83	60S ribosomal protein L36a
53	TRINITY_DN45102_c0_g1	283	54.81	8.56	0.00	0.00	-8.81	Transposon Ty3-I Gag-Pol polyprotein
54	TRINITY_DN32703_c0_g1	619	55.34	3.95	0.00	0.00	-8.81	40S ribosomal protein S7
55	TRINITY_DN1461_c0_g2	500	55.23	4.88	0.00	0.00	-8.81	40S ribosomal protein S20
56	TRINITY_DN25561_c0_g2	268	55.34	9.13	0.00	0.00	-8.81	40S ribosomal protein S19
57	TRINITY_DN53410_c0_g1	463	54.35	5.19	0.00	0.00	-8.78	Transposon Ty3-I Gag-Pol polyprotein
58	TRINITY_DN28197_c1_g2	2545	54.21	0.94	0.02	0.00	-8.78	Pentatricopeptide repeat-containing protein At2g03380, mitochondrial
59	TRINITY_DN34049_c1_g1	1076	53.50	2.20	0.00	0.00	-8.78	60S acidic ribosomal protein P0
60	TRINITY_DN36918_c0_g1	687	53.69	3.46	0.00	0.00	-8.78	Retrovirus-related Pol polyprotein from transposon 17.6
61	TRINITY_DN34776_c1_g3	1799	51.62	1.27	0.29	0.01	-8.72	Protein FAR1-RELATED SEQUENCE 3
62	TRINITY_DN56229_c0_g1	274	51.59	8.33	0.00	0.00	-8.72	Keratin, type I cytoskeletal 50 kDa
63	TRINITY_DN33626_c0_g1	586.62	50.62	3.81	0.00	0.00	-8.70	40S ribosomal protein SA
64	TRINITY_DN33664_c0_g1	548	50.27	4.06	0.00	0.00	-8.67	60S ribosomal protein L21
65	TRINITY_DN23825_c0_g1	369	49.13	5.89	0.00	0.00	-8.64	60S ribosomal protein L39
66	TRINITY_DN28081_c0_g1	403	48.86	5.36	0.00	0.00	-8.64	60S acidic ribosomal protein P2
67	TRINITY_DN33455_c0_g1	680	49.31	3.21	0.00	0.00	-8.64	40S ribosomal protein S8
68	TRINITY_DN21510_c0_g1	467	49.40	4.68	0.00	0.00	-8.64	Chlorophyll a-b binding protein 3, chloroplastic
69	TRINITY_DN34427_c0_g1	1030	48.02	2.06	0.01	0.00	-8.61	Probable galacturonosyltransferase-like 2
70	TRINITY_DN31999_c0_g3	1011	47.93	2.10	0.00	0.00	-8.61	VQ motif-containing protein 31
71	TRINITY_DN36465_c0_g1	285	47.56	7.38	0.00	0.00	-8.61	50S ribosomal protein L2, chloroplastic
72	TRINITY_DN22297_c0_g1	713	45.72	2.84	0.00	0.00	-8.55	Two-component response regulator ORR9
73	TRINITY_DN7981_c0_g1	280	46.25	7.30	0.00	0.00	-8.55	Ethylene receptor 1
74	TRINITY_DN55594_c0_g1	302	44.82	6.56	0.00	0.00	-8.52	Protein Ycf2
75	TRINITY_DN3695_c0_g1	1152	44.87	1.72	0.17	0.01	-8.52	PLAT domain-containing protein 2

*logFC: the logarithm to base 2 of fold change (Salt/Control)

**TPM: transcripts per million

Supplementary Table 1. (Cont) Annotation profile of DEG in ice plant seedlings treated with 200 mM NaCl (|FC|>4, FDR<0.001)

No.	Transcript ID	Length	Control reads	Control reads (TPM**)	Salt reads	Salt reads (TPM)	logFC*	Annotation
76	TRINITY_DN31685_c0_g1	467	44.86	4.25	0.00	0.00	-8.52	60S ribosomal protein L14
77	TRINITY_DN32559_c1_g2	376	44.76	5.26	0.00	0.00	-8.52	Copia protein
78	TRINITY_DN29350_c0_g1	480	44.06	4.06	0.00	0.00	-8.48	60S ribosomal protein L23
79	TRINITY_DN49811_c0_g1	274	43.97	7.10	0.00	0.00	-8.48	26S proteasome non-ATPase regulatory subunit 10
80	TRINITY_DN32228_c0_g1	820	44.18	2.38	0.00	0.00	-8.48	40S ribosomal protein S3a
81	TRINITY_DN23491_c0_g2	439	43.82	4.41	0.00	0.00	-8.48	60S ribosomal protein L35
82	TRINITY_DN33228_c0_g1	828	43.03	2.30	0.00	0.00	-8.45	Carboxylesterase 1
83	TRINITY_DN5042_c0_g1	267	42.71	7.07	0.00	0.00	-8.45	UPF0271 protein TTHB195
84	TRINITY_DN34118_c2_g1	370	43.38	5.18	0.06	0.01	-8.45	Cytochrome P450 78A5
85	TRINITY_DN22559_c0_g1	317	43.02	6.00	0.00	0.00	-8.45	MLP-like protein 43
86	TRINITY_DN34347_c0_g1	1505	41.79	1.23	0.06	0.00	-8.42	Regulator of nonsense transcripts UPF2
87	TRINITY_DN17219_c0_g1	436	42.01	4.26	0.00	0.00	-8.42	Eukaryotic initiation factor 4A-9
88	TRINITY_DN30954_c0_g4	709	40.75	2.54	0.14	0.01	-8.38	Polygalacturonase non-catalytic subunit AroGP2
89	TRINITY_DN28694_c0_g1	817	41.07	2.22	0.00	0.00	-8.38	40S ribosomal protein S3
90	TRINITY_DN20089_c0_g1	652	41.30	2.80	0.00	0.00	-8.38	Keratin, type I cytoskeletal 50 kDa
91	TRINITY_DN35429_c0_g1	576	39.59	3.04	0.00	0.00	-8.35	Transposon Tf2-11 polyprotein
92	TRINITY_DN37713_c0_g1	282	39.81	6.24	0.00	0.00	-8.35	Pol polyprotein
93	TRINITY_DN740_c1_g1	331	39.59	5.29	0.00	0.00	-8.35	Acetoacetyl-CoA synthetase
94	TRINITY_DN32900_c0_g1	456	39.51	3.83	0.00	0.00	-8.35	60S ribosomal protein L28
95	TRINITY_DN51664_c0_g1	273	40.23	6.52	0.00	0.00	-8.35	Maturase K
96	TRINITY_DN29468_c0_g2	1736	40.34	1.03	0.32	0.01	-8.35	Ethylene-responsive transcription factor ERF061
97	TRINITY_DN30954_c0_g3	709	39.65	2.47	0.09	0.00	-8.35	Polygalacturonase-1 non-catalytic subunit beta
98	TRINITY_DN27754_c2_g1	301	39.93	5.87	0.00	0.00	-8.35	Pentatricopeptide repeat-containing protein At1g05750, chloroplastic
99	TRINITY_DN43337_c0_g1	333	39.00	5.18	0.00	0.00	-8.31	Receptor-like protein kinase
100	TRINITY_DN34275_c4_g3	1313	39.18	1.32	0.26	0.01	-8.31	Peptide methionine sulfoxide reductase B2, chloroplastic

*logFC: the logarithm to base 2 of fold change (Salt/Control)

**TPM: transcripts per million

Supplementary Table 1. (Cont) Annotation profile of DEG in ice plant seedlings treated with 200 mM NaCl (|FC|>4, FDR<0.001)

No.	Transcript ID	Length	Control reads	Control reads (TPM**)	Salt reads	Salt reads (TPM)	logFC*	Annotation
101	TRINITY_DN22638_c0_g1	396	38.75	4.33	0.00	0.00	-8.31	40S ribosomal protein S15a
102	TRINITY_DN31153_c0_g1	506	38.60	3.37	0.00	0.00	-8.31	40S ribosomal protein S14
103	TRINITY_DN29125_c0_g1	433.31	39.40	4.02	0.00	0.00	-8.31	60S ribosomal protein L37a
104	TRINITY_DN29492_c0_g1	541	37.94	3.10	0.00	0.00	-8.27	60S ribosomal protein L19
105	TRINITY_DN44920_c0_g1	287	37.67	5.80	0.00	0.00	-8.27	Retrovirus-related Pol polyprotein from transposon 17.6
106	TRINITY_DN41709_c0_g1	532	37.68	3.13	0.00	0.00	-8.27	Serpin-ZX
107	TRINITY_DN29413_c0_g1	609	37.66	2.73	0.00	0.00	-8.27	60S ribosomal protein L17
108	TRINITY_DN29990_c0_g2	384	38.15	4.39	0.00	0.00	-8.27	60S ribosomal protein L34
109	TRINITY_DN28345_c0_g1	1474	38.00	1.14	0.02	0.00	-8.27	Pentatricopeptide repeat-containing protein At1g77360, mitochondrial
110	TRINITY_DN25110_c0_g1	602	37.65	2.77	0.00	0.00	-8.27	60S ribosomal protein L18
111	TRINITY_DN33556_c0_g1	854	36.64	1.90	0.00	0.00	-8.23	60S ribosomal protein L6
112	TRINITY_DN23265_c0_g2	600	36.66	2.70	0.00	0.00	-8.23	Intermediate filament protein ON3
113	TRINITY_DN7912_c0_g1	867	36.60	1.87	0.00	0.00	-8.23	Retrovirus-related Pol polyprotein from transposon 17.6
114	TRINITY_DN22878_c0_g1	938	37.11	1.75	0.00	0.00	-8.23	DNA repair protein RAD51 homolog 3
115	TRINITY_DN26380_c0_g1	820	36.48	1.97	0.00	0.00	-8.20	40S ribosomal protein S6
116	TRINITY_DN50905_c0_g1	386	36.13	4.14	0.00	0.00	-8.20	Ribosome biogenesis protein RLP24
117	TRINITY_DN43146_c0_g1	995	35.67	1.59	0.00	0.00	-8.20	60S ribosomal protein L5
118	TRINITY_DN35028_c6_g9	1937	34.91	0.80	0.13	0.00	-8.16	Phosphoribosylaminoimidazole-succinocarboxamide synthase, chloroplastic
119	TRINITY_DN30199_c0_g2	919	35.12	1.69	0.00	0.00	-8.16	BEL1-like homeodomain protein 11
120	TRINITY_DN48732_c0_g1	276	35.30	5.66	0.00	0.00	-8.16	Elongation factor 2
121	TRINITY_DN31669_c0_g2	685	35.12	2.27	0.00	0.00	-8.16	40S ribosomal protein S4, X isoform
122	TRINITY_DN1440_c0_g1	512	34.89	3.01	0.00	0.00	-8.16	Late embryogenesis abundant protein 46
123	TRINITY_DN29284_c0_g1	487	34.58	3.14	0.00	0.00	-8.16	60S ribosomal protein L23a
124	TRINITY_DN34821_c1_g1	418	34.54	3.65	0.01	0.00	-8.16	Cellulose synthase-like protein E1
125	TRINITY_DN34162_c0_g1	279	34.32	5.44	0.00	0.00	-8.11	Auxin response factor 5

*logFC: the logarithm to base 2 of fold change (Salt/Control)

**TPM: transcripts per million

Supplementary Table 1. (Cont) Annotation profile of DEG in ice plant seedlings treated with 200 mM NaCl (|FC|>4, FDR<0.001)

No.	Transcript ID	Length	Control reads	Control reads (TPM**)	Salt reads	Salt reads (TPM)	logFC*	Annotation
126	TRINITY_DN30766_c1_g1	474	33.70	3.14	0.00	0.00	-8.11	60S ribosomal protein L13a
127	TRINITY_DN40335_c0_g1	344	34.42	4.42	0.00	0.00	-8.11	Transposon Tf2-9 polyprotein
128	TRINITY_DN57710_c0_g1	394	33.59	3.77	0.00	0.00	-8.11	Photosystem II protein D1
129	TRINITY_DN30712_c0_g1	715	34.48	2.13	0.00	0.00	-8.11	Heat shock cognate 71 kDa protein
130	TRINITY_DN50615_c0_g1	282	33.81	5.30	0.00	0.00	-8.11	40S ribosomal protein S23
131	TRINITY_DN31764_c0_g3	2373	32.88	0.61	0.32	0.01	-8.07	Protein STRUBBELIG-RECEPTOR FAMILY 8
132	TRINITY_DN43226_c0_g1	290	33.26	5.07	0.00	0.00	-8.07	Protein Ycf2
133	TRINITY_DN33786_c0_g3	753	32.54	1.91	0.00	0.00	-8.07	Transcription factor BIM1
134	TRINITY_DN49605_c0_g1	350	32.89	4.16	0.00	0.00	-8.07	Retrovirus-related Pol polyprotein from transposon 17.6
135	TRINITY_DN54643_c0_g1	320	33.40	4.62	0.00	0.00	-8.07	Type I inositol polyphosphate 5-phosphatase 12
136	TRINITY_DN45315_c0_g1	269	31.68	5.21	0.00	0.00	-8.03	Pleckstrin-2
137	TRINITY_DN1196_c0_g1	278	31.91	5.08	0.00	0.00	-8.03	Zinc/cadmium resistance protein
138	TRINITY_DN32183_c1_g3	588	31.96	2.40	0.00	0.00	-8.03	Gamma-glutamyl hydrolase
139	TRINITY_DN13090_c0_g1	349	31.75	4.02	0.00	0.00	-8.03	Natural killer cells antigen CD94
140	TRINITY_DN34250_c0_g2	1172	30.72	1.16	0.37	0.01	-7.98	Probable dolichyl pyrophosphate Glc1Man9GlcNAc2 alpha-1,3-glucosyltransferase
141	TRINITY_DN5734_c0_g2	485	31.41	2.86	0.00	0.00	-7.98	Heavy metal-associated isoprenylated plant protein 26
142	TRINITY_DN27516_c0_g2	621	30.63	2.18	0.00	0.00	-7.98	Protein EPIDERMAL PATTERNING FACTOR 1
143	TRINITY_DN51163_c0_g1	293	30.69	4.63	0.00	0.00	-7.98	Transposon Tf2-11 polyprotein
144	TRINITY_DN35654_c0_g1	483	30.88	2.83	0.00	0.00	-7.98	Leucine-rich repeat extensin-like protein 4
145	TRINITY_DN52172_c0_g1	290	31.17	4.75	0.00	0.00	-7.98	Ribulose bisphosphate carboxylase small chain 3B, chloroplastic
146	TRINITY_DN26850_c0_g1	571	31.37	2.43	0.00	0.00	-7.98	Endoglucanase 2
147	TRINITY_DN22386_c0_g1	981	30.94	1.39	0.00	0.00	-7.98	Probable BOI-related E3 ubiquitin-protein ligase 3
148	TRINITY_DN44241_c0_g1	315	30.98	4.35	0.00	0.00	-7.98	Retrovirus-related Pol polyprotein from transposon 297
149	TRINITY_DN28300_c1_g1	365	29.63	3.59	0.00	0.00	-7.93	Putative pentatricopeptide repeat-containing protein At5g37570
150	TRINITY_DN17136_c0_g1	1923	30.43	0.70	0.00	0.00	-7.93	Pentatricopeptide repeat-containing protein At2g45350, chloroplastic

*logFC: the logarithm to base 2 of fold change (Salt/Control)

**TPM: transcripts per million

Supplementary Table 1. (Cont) Annotation profile of DEG in ice plant seedlings treated with 200 mM NaCl (|FC|>4, FDR<0.001)

No.	Transcript ID	Length	Control reads	Control reads (TPM**)	Salt reads	Salt reads (TPM)	logFC*	Annotation
151	TRINITY_DN24855_c0_g1	688	29.71	1.91	0.00	0.00	-7.93	Translationally-controlled tumor protein homolog
152	TRINITY_DN42809_c0_g1	419	29.61	3.13	0.00	0.00	-7.93	Protein Ycf2
153	TRINITY_DN26901_c0_g2	2273	29.88	0.58	0.38	0.01	-7.93	Kinesin-like protein KIN-7E
154	TRINITY_DN39156_c0_g1	285	29.67	4.60	0.00	0.00	-7.93	Copia protein
155	TRINITY_DN43811_c0_g1	315	30.12	4.23	0.00	0.00	-7.93	40S ribosomal protein S23
156	TRINITY_DN33036_c0_g1	517	30.06	2.57	0.00	0.00	-7.93	60S ribosomal protein L10a
157	TRINITY_DN22003_c0_g4	454	28.55	2.78	0.00	0.00	-7.88	40S ribosomal protein S10
158	TRINITY_DN34952_c0_g2	1795	29.22	0.72	0.00	0.00	-7.88	E3 ubiquitin-protein ligase listerin
159	TRINITY_DN46492_c0_g1	285	29.45	4.57	0.00	0.00	-7.88	Protein Ycf2
160	TRINITY_DN23272_c0_g1	363	29.22	3.56	0.00	0.00	-7.88	Chaperone protein DnaK
161	TRINITY_DN35170_c0_g4	1228	28.67	1.03	0.00	0.00	-7.88	Protein SUPPRESSOR OF PHYA-105 1
162	TRINITY_DN35468_c0_g1	309	28.64	4.10	0.00	0.00	-7.88	Alpha,alpha-trehalose-phosphate synthase
163	TRINITY_DN47567_c0_g1	267	28.51	4.72	0.00	0.00	-7.88	Uncharacterized mitochondrial protein AtMg00750
164	TRINITY_DN24335_c0_g1	668	28.20	1.87	0.00	0.00	-7.83	Uncharacterized protein At2g39795, mitochondrial
165	TRINITY_DN42375_c0_g1	277	28.26	4.51	0.00	0.00	-7.83	Phosphoinositide phospholipase C 4
166	TRINITY_DN27975_c0_g1	635	28.15	1.96	0.00	0.00	-7.83	WUSCHEL-related homeobox 9
167	TRINITY_DN21777_c1_g1	271	27.89	4.55	0.00	0.00	-7.83	Pentatricopeptide repeat-containing protein At3g26782, mitochondrial
168	TRINITY_DN23154_c0_g3	1380	27.51	0.88	0.38	0.01	-7.83	Protein IQ-DOMAIN 32
169	TRINITY_DN54640_c0_g1	302	27.75	4.06	0.00	0.00	-7.83	40S ribosomal protein S15
170	TRINITY_DN39608_c0_g1	301	28.30	4.16	0.00	0.00	-7.83	Pro-Pol polyprotein
171	TRINITY_DN24543_c0_g1	457	27.78	2.69	0.00	0.00	-7.83	60S ribosomal protein L10-like
172	TRINITY_DN11532_c0_g1	505	27.74	2.43	0.00	0.00	-7.83	60S ribosomal protein L11
173	TRINITY_DN9090_c1_g1	313	27.50	3.89	0.00	0.00	-7.83	Retrovirus-related Pol polyprotein from transposon 297
174	TRINITY_DN48207_c0_g2	798	28.07	1.56	0.44	0.02	-7.83	DNA-directed RNA polymerases II, IV and V subunit 11
175	TRINITY_DN32385_c0_g1	481	27.76	2.55	0.00	0.00	-7.83	60S ribosomal protein L7a

*logFC: the logarithm to base 2 of fold change (Salt/Control)

**TPM: transcripts per million

Supplementary Table 1. (Cont) Annotation profile of DEG in ice plant seedlings treated with 200 mM NaCl (|FC|>4, FDR<0.001)

No.	Transcript ID	Length	Control reads	Control reads (TPM**)	Salt reads	Salt reads (TPM)	logFC*	Annotation
176	TRINITY_DN14112_c0_g2	294	28.37	4.27	0.00	0.00	-7.83	Uncharacterized protein ORF91
177	TRINITY_DN23435_c0_g1	312	27.88	3.95	0.00	0.00	-7.83	40S ribosomal protein S12
178	TRINITY_DN19045_c0_g1	605	26.62	1.95	0.00	0.00	-7.78	Collagen alpha-1(VIII) chain
179	TRINITY_DN37239_c0_g1	298	26.86	3.99	0.00	0.00	-7.78	Protein Ycf2
180	TRINITY_DN33497_c0_g1	826	26.57	1.42	0.00	0.00	-7.78	Keratin, type II cytoskeletal 8
181	TRINITY_DN29712_c0_g1	666	26.66	1.76	0.00	0.00	-7.78	60S ribosomal protein L9
182	TRINITY_DN27971_c0_g2	288	26.75	4.11	0.00	0.00	-7.78	60S ribosomal protein L10
183	TRINITY_DN1196_c1_g1	269	26.61	4.37	0.00	0.00	-7.78	Zinc homeostasis factor 1
184	TRINITY_DN30163_c1_g1	297	25.95	3.86	0.00	0.00	-7.73	Phosphoenolpyruvate carboxykinase
185	TRINITY_DN27828_c0_g1	601	25.87	1.90	0.00	0.00	-7.73	Protein argonaute 14
186	TRINITY_DN5483_c0_g1	534	25.96	2.15	0.00	0.00	-7.73	60S ribosomal protein L24
187	TRINITY_DN47612_c0_g1	873	25.52	1.29	0.00	0.00	-7.73	Catalase isozyme A
188	TRINITY_DN23650_c0_g1	343	25.70	3.31	0.00	0.00	-7.73	60S ribosomal protein L26
189	TRINITY_DN4476_c0_g1	2450	25.53	0.46	0.01	0.00	-7.73	Zinc finger BED domain-containing protein RICESLEEPER 2
190	TRINITY_DN8394_c1_g1	377	26.45	3.10	0.00	0.00	-7.73	Probable glucan endo-1,3-beta-glucosidase BG4
191	TRINITY_DN28626_c0_g2	411	25.63	2.76	0.00	0.00	-7.73	Pentatricopeptide repeat-containing protein At3g49740
192	TRINITY_DN28457_c0_g1	304	25.66	3.73	0.00	0.00	-7.73	Ribonuclease S-2
193	TRINITY_DN29209_c0_g1	766	25.87	1.49	0.00	0.00	-7.73	Collagen alpha-1(I) chain
194	TRINITY_DN26938_c0_g3	538	25.88	2.13	0.00	0.00	-7.73	Inorganic phosphate transporter 1-4
195	TRINITY_DN47887_c0_g1	455	25.60	2.49	0.00	0.00	-7.73	Maturase K
196	TRINITY_DN39401_c0_g1	296	26.32	3.93	0.00	0.00	-7.73	Chlorophyll a-b binding protein 1D
197	TRINITY_DN28299_c0_g1	556	25.77	2.05	0.00	0.00	-7.73	Elongation factor 1-alpha
198	TRINITY_DN32482_c0_g1	550	25.79	2.07	0.00	0.00	-7.73	60S ribosomal protein L15
199	TRINITY_DN12390_c0_g2	413	25.21	2.70	0.00	0.00	-7.67	Uncharacterized protein YpeP
200	TRINITY_DN44668_c0_g1	383	24.62	2.84	0.00	0.00	-7.67	Oxygen-evolving enhancer protein 2-1, chloroplastic

*logFC: the logarithm to base 2 of fold change (Salt/Control)

**TPM: transcripts per million

Supplementary Table 1. (Cont) Annotation profile of DEG in ice plant seedlings treated with 200 mM NaCl (|FC|>4, FDR<0.001)

No.	Transcript ID	Length	Control reads	Control reads (TPM**)	Salt reads	Salt reads (TPM)	logFC*	Annotation
201	TRINITY_DN28615_c0_g2	552	25.28	2.02	0.00	0.00	-7.67	ATP-dependent DNA helicase SRS2-like protein At4g25120
202	TRINITY_DN49707_c0_g1	267	24.95	4.13	0.00	0.00	-7.67	Glycine-rich RNA-binding protein
203	TRINITY_DN30734_c1_g1	318	24.55	3.41	0.00	0.00	-7.67	Keratin, type I cytoskeletal 18
204	TRINITY_DN25353_c0_g2	966	25.20	1.15	0.02	0.00	-7.67	U1 small nuclear ribonucleoprotein A
205	TRINITY_DN30526_c0_g1	1003	25.48	1.12	0.00	0.00	-7.67	60S ribosomal protein L3
206	TRINITY_DN2947_c0_g1	3259	25.07	0.34	0.31	0.00	-7.67	Pentatricopeptide repeat-containing protein At1g25360
207	TRINITY_DN32132_c0_g1	443	25.01	2.44	0.00	0.00	-7.67	Photosystem II 10 kDa polypeptide, chloroplastic
208	TRINITY_DN28855_c0_g1	338	25.29	3.31	0.00	0.00	-7.67	40S ribosomal protein S19
209	TRINITY_DN31763_c3_g1	298	24.60	3.65	0.00	0.00	-7.67	Callose synthase 11
210	TRINITY_DN45960_c0_g1	283	25.30	3.95	0.00	0.00	-7.67	Golgi apparatus membrane protein TVP23
211	TRINITY_DN34184_c0_g3	1422	25.04	0.78	0.00	0.00	-7.67	Uncharacterized protein At1g04910
212	TRINITY_DN35724_c0_g1	289	25.20	3.86	0.00	0.00	-7.67	Elongation factor 1-delta
213	TRINITY_DN53938_c0_g2	1595	1212.99	33.63	5.57	0.14	-7.67	50S ribosomal protein L1
214	TRINITY_DN13750_c0_g1	2468	23.71	0.42	0.16	0.00	-7.61	Separase
215	TRINITY_DN23435_c0_g2	312	23.52	3.33	0.00	0.00	-7.61	40S ribosomal protein S12
216	TRINITY_DN6799_c0_g1	282	23.79	3.73	0.00	0.00	-7.61	Glutathione S-transferase U16
217	TRINITY_DN29762_c0_g1	1005	24.33	1.07	0.00	0.00	-7.61	Kelch-like protein 4
218	TRINITY_DN29731_c0_g1	579.02	24.48	1.36	0.00	0.00	-7.61	60S ribosomal protein L4
219	TRINITY_DN37673_c0_g1	370	23.60	2.82	0.00	0.00	-7.61	Photosystem I reaction center subunit VIII
220	TRINITY_DN24558_c0_g2	870	24.40	1.24	0.00	0.00	-7.61	Kinesin-like protein NACK1
221	TRINITY_DN5082_c1_g1	288	23.96	3.68	0.00	0.00	-7.61	Serine/threonine-protein kinase RUNKEL
222	TRINITY_DN31604_c0_g1	464	24.06	2.29	0.00	0.00	-7.61	60S acidic ribosomal protein P1
223	TRINITY_DN20569_c0_g1	317	23.52	3.28	0.00	0.00	-7.61	Conserved oligomeric Golgi complex subunit 5
224	TRINITY_DN31527_c0_g2	413	24.44	2.62	0.00	0.00	-7.61	60S ribosomal protein L31
225	TRINITY_DN48643_c1_g1	302	23.70	3.47	0.00	0.00	-7.61	Pro-Pol polyprotein

*logFC: the logarithm to base 2 of fold change (Salt/Control)

**TPM: transcripts per million

Supplementary Table 1. (Cont) Annotation profile of DEG in ice plant seedlings treated with 200 mM NaCl (|FC|>4, FDR<0.001)

No.	Transcript ID	Length	Control reads	Control reads (TPM**)	Salt reads	Salt reads (TPM)	logFC*	Annotation
226	TRINITY_DN54428_c0_g1	333	24.25	3.22	0.00	0.00	-7.61	Cytochrome b-c1 complex subunit 2, mitochondrial
227	TRINITY_DN35107_c0_g4	759	23.57	1.37	0.00	0.00	-7.61	ABC transporter A family member 12
228	TRINITY_DN29109_c0_g1	387	23.80	2.72	0.00	0.00	-7.61	60S ribosomal protein L22
229	TRINITY_DN46756_c0_g1	291	23.24	3.53	0.00	0.00	-7.55	Retrovirus-related Pol polyprotein from transposon opus
230	TRINITY_DN42687_c0_g1	328	22.95	3.09	0.00	0.00	-7.55	Probable LRR receptor-like serine/threonine-protein kinase RPK1
231	TRINITY_DN7001_c0_g1	297	23.45	3.49	0.00	0.00	-7.55	NifU-like protein C1709.19c
232	TRINITY_DN47745_c0_g1	959	22.88	1.05	0.00	0.00	-7.55	YTH domain-containing family protein 1
233	TRINITY_DN31608_c0_g1	2453	23.22	0.42	0.14	0.00	-7.55	Scarecrow-like protein 14
234	TRINITY_DN37440_c0_g1	371	23.15	2.76	0.00	0.00	-7.55	Thymosin beta-11
235	TRINITY_DN22110_c0_g2	283	22.66	3.54	0.00	0.00	-7.55	60S ribosomal protein L36
236	TRINITY_DN38264_c0_g1	426	22.73	2.36	0.00	0.00	-7.55	Histidinol-phosphate aminotransferase
237	TRINITY_DN29722_c0_g1	474	22.67	2.12	0.00	0.00	-7.55	Glycine-rich RNA-binding protein
238	TRINITY_DN28275_c0_g2	365	22.79	2.76	0.00	0.00	-7.55	Actin, cytoplasmic 1
239	TRINITY_DN31696_c0_g1	389	21.74	2.47	0.00	0.00	-7.49	Indole-3-acetic acid-amido synthetase GH3.3
240	TRINITY_DN2412_c0_g1	420	22.30	2.35	0.00	0.00	-7.49	54S ribosomal protein L24, mitochondrial
241	TRINITY_DN39704_c0_g1	289	22.06	3.38	0.00	0.00	-7.49	DnaJ homolog subfamily B member 4
242	TRINITY_DN22003_c0_g1	454	21.64	2.11	0.00	0.00	-7.49	40S ribosomal protein S10
243	TRINITY_DN33412_c1_g1	492	22.23	2.00	0.00	0.00	-7.49	Vacuolar protein sorting-associated protein 54, chloroplastic
244	TRINITY_DN43028_c0_g1	280	21.68	3.42	0.00	0.00	-7.49	Fructose-bisphosphate aldolase, chloroplastic
245	TRINITY_DN30789_c0_g1	469	22.17	2.09	0.00	0.00	-7.49	60S ribosomal protein L27
246	TRINITY_DN27573_c0_g1	267	21.72	3.60	0.00	0.00	-7.49	Homocitrate synthase, mitochondrial
247	TRINITY_DN44098_c0_g1	296	22.15	3.31	0.00	0.00	-7.49	Gamma-glutamyltranspeptidase 1
248	TRINITY_DN7951_c0_g2	472	21.84	2.05	0.00	0.00	-7.49	Pentatricopeptide repeat-containing protein At3g16610
249	TRINITY_DN45420_c0_g1	298	22.33	3.31	0.00	0.00	-7.49	Peptidyl-prolyl cis-trans isomerase NIMA-interacting 4
250	TRINITY_DN32268_c0_g3	420	21.74	2.29	0.00	0.00	-7.49	60S ribosomal protein L23a

*logFC: the logarithm to base 2 of fold change (Salt/Control)

**TPM: transcripts per million

Supplementary Table 1. (Cont) Annotation profile of DEG in ice plant seedlings treated with 200 mM NaCl (|FC|>4, FDR<0.001)

No.	Transcript ID	Length	Control reads	Control reads (TPM**)	Salt reads	Salt reads (TPM)	logFC*	Annotation
251	TRINITY_DN10327_c0_g2	601	22.10	1.63	0.00	0.00	-7.49	60S ribosomal protein L8
252	TRINITY_DN30179_c0_g2	397	22.03	2.45	0.00	0.00	-7.49	Putative pentatricopeptide repeat-containing protein At5g08310, mitochondrial
253	TRINITY_DN3863_c0_g2	1060	21.83	0.91	0.39	0.02	-7.49	Glutamate receptor 2.6
254	TRINITY_DN24746_c0_g1	356	22.11	2.75	0.00	0.00	-7.49	3-ketoacyl-CoA thiolase, peroxisomal
255	TRINITY_DN55477_c0_g1	279	21.64	3.43	0.00	0.00	-7.49	LRR receptor kinase BAK1
256	TRINITY_DN31120_c0_g1	1521	22.35	0.65	0.00	0.00	-7.49	Chloroplast sensor kinase, chloroplastic
257	TRINITY_DN33350_c0_g3	1085	21.61	0.88	0.00	0.00	-7.49	Tyrosine aminotransferase
258	TRINITY_DN45756_c0_g1	278	22.27	3.54	0.00	0.00	-7.49	NEDD8-activating enzyme E1 regulatory subunit
259	TRINITY_DN41825_c0_g2	1702	21.82	0.57	0.00	0.00	-7.49	Serine/threonine-protein kinase mph1
260	TRINITY_DN54687_c0_g2	525	21.84	1.84	0.00	0.00	-7.49	60S ribosomal protein L5
261	TRINITY_DN13371_c0_g1	480	22.02	2.03	0.00	0.00	-7.49	AT-rich interactive domain-containing protein 3
262	TRINITY_DN44534_c0_g1	285	21.04	3.26	0.00	0.00	-7.42	Phosphoserine aminotransferase
263	TRINITY_DN22716_c0_g2	618	20.56	1.47	0.00	0.00	-7.42	Mitochondrial import inner membrane translocase subunit TIM23-1
264	TRINITY_DN24278_c0_g2	300	20.93	3.08	0.00	0.00	-7.42	Eukaryotic translation initiation factor 5A-2
265	TRINITY_DN35028_c0_g2	460	21.23	2.04	0.00	0.00	-7.42	Sialyltransferase-like protein 1
266	TRINITY_DN43304_c0_g1	335	21.18	2.80	0.00	0.00	-7.42	50S ribosomal protein L2-A, chloroplastic
267	TRINITY_DN53862_c0_g1	698	21.44	1.36	0.00	0.00	-7.42	BURP domain protein USPL1
268	TRINITY_DN21851_c0_g1	750	21.44	1.26	0.00	0.00	-7.42	Abscisic stress-ripening protein 3
269	TRINITY_DN30526_c0_g2	1003	21.45	0.95	0.00	0.00	-7.42	60S ribosomal protein L3
270	TRINITY_DN3617_c0_g1	378	20.67	2.42	0.00	0.00	-7.42	40S ribosomal protein S2
271	TRINITY_DN42250_c0_g1	301	20.51	3.01	0.00	0.00	-7.42	Protein Ycf2
272	TRINITY_DN53672_c0_g1	453	21.32	2.08	0.00	0.00	-7.42	ATP synthase subunit alpha, chloroplastic
273	TRINITY_DN26697_c3_g1	871	20.62	1.05	0.43	0.02	-7.42	Probable E3 ubiquitin-protein ligase ARI8
274	TRINITY_DN49220_c0_g1	618	21.18	1.52	0.00	0.00	-7.42	Nucleoside diphosphate kinase
275	TRINITY_DN1333_c0_g1	268	21.35	3.52	0.00	0.00	-7.42	UDP-glucose:glycoprotein glucosyltransferase

*logFC: the logarithm to base 2 of fold change (Salt/Control)

**TPM: transcripts per million

Supplementary Table 1. (Cont) Annotation profile of DEG in ice plant seedlings treated with 200 mM NaCl (|FC|>4, FDR<0.001)

No.	Transcript ID	Length	Control reads	Control reads (TPM**)	Salt reads	Salt reads (TPM)	logFC*	Annotation
276	TRINITY_DN18691_c0_g2	566	21.23	1.66	0.15	0.01	-7.42	Thioredoxin H-type
277	TRINITY_DN39635_c0_g1	291	20.83	3.17	0.00	0.00	-7.42	Putative ribosome biogenesis protein C8F11.04
278	TRINITY_DN22141_c0_g1	342	20.75	2.68	0.00	0.00	-7.42	Oryzain alpha chain
279	TRINITY_DN50470_c0_g2	546	20.10	1.63	0.00	0.00	-7.35	Phosphoglycerate kinase
280	TRINITY_DN3130_c0_g1	333	20.45	2.72	0.00	0.00	-7.35	Ribulose bisphosphate carboxylase small chain
281	TRINITY_DN36672_c1_g1	308	20.03	2.88	0.00	0.00	-7.35	Pro-Pol polyprotein
282	TRINITY_DN35418_c3_g6	960	19.72	0.91	0.00	0.00	-7.35	Peroxidase 41
283	TRINITY_DN37888_c0_g1	269	20.47	3.36	0.00	0.00	-7.35	Chlorophyll a-b binding protein 4, chloroplastic
284	TRINITY_DN29761_c0_g1	463	19.68	1.88	0.00	0.00	-7.35	Copper transport protein ATX1
285	TRINITY_DN25190_c0_g2	1190	19.91	0.74	0.00	0.00	-7.35	tRNA
286	TRINITY_DN30467_c0_g1	2051	20.32	0.44	0.00	0.00	-7.35	Phosphoglucan phosphatase LSF1, chloroplastic
287	TRINITY_DN51399_c0_g1	409	19.51	2.11	0.00	0.00	-7.35	Guanine nucleotide-binding protein subunit beta-2-like 1
288	TRINITY_DN54045_c0_g1	317	19.75	2.75	0.00	0.00	-7.35	Eukaryotic peptide chain release factor GTP-binding subunit
289	TRINITY_DN49912_c0_g1	382	19.64	2.27	0.00	0.00	-7.35	Endoplasmin homolog
290	TRINITY_DN54595_c0_g1	321	19.89	2.74	0.00	0.00	-7.35	Succinyl-CoA:3-ketoacid coenzyme A transferase 1, mitochondrial
291	TRINITY_DN25390_c0_g3	924	19.88	0.95	0.00	0.00	-7.35	Abscisic stress-ripening protein 2
292	TRINITY_DN49172_c0_g1	315	20.02	2.81	0.00	0.00	-7.35	DNA-directed RNA polymerase subunit beta"
293	TRINITY_DN54370_c0_g1	292	19.89	3.01	0.00	0.00	-7.35	3'(2'),5'-bisphosphate nucleotidase 1
294	TRINITY_DN8195_c0_g2	751	20.02	1.18	0.02	0.00	-7.35	Ubiquitin-fold modifier-conjugating enzyme 1
295	TRINITY_DN39000_c0_g1	280	19.60	3.10	0.00	0.00	-7.35	Nucleosome assembly protein 1;2
296	TRINITY_DN447_c0_g1	270	19.00	3.11	0.00	0.00	-7.28	DNA polymerase epsilon subunit C
297	TRINITY_DN27814_c0_g1	273	19.43	3.15	0.00	0.00	-7.28	Cysteine proteinase 2
298	TRINITY_DN49836_c0_g1	394	19.21	2.16	0.00	0.00	-7.28	Uncharacterized mitochondrial protein AtMg00300
299	TRINITY_DN52872_c0_g1	686	19.24	1.24	0.00	0.00	-7.28	DEAD-box ATP-dependent RNA helicase 26
300	TRINITY_DN42823_c0_g1	402	18.91	2.08	0.00	0.00	-7.28	Protein Ycf2

*logFC: the logarithm to base 2 of fold change (Salt/Control)

**TPM: transcripts per million

Supplementary Table 1. (Cont) Annotation profile of DEG in ice plant seedlings treated with 200 mM NaCl (|FC|>4, FDR<0.001)

No.	Transcript ID	Length	Control reads	Control reads (TPM**)	Salt reads	Salt reads (TPM)	logFC*	Annotation
301	TRINITY_DN40359_c0_g1	278	19.07	3.03	0.00	0.00	-7.28	40S ribosomal protein S12
302	TRINITY_DN40316_c0_g1	271	19.21	3.14	0.00	0.00	-7.28	Keratin, type I cytoskeletal 18
303	TRINITY_DN38142_c0_g1	267	19.48	3.23	0.00	0.00	-7.28	Thymosin beta-a
304	TRINITY_DN22886_c0_g1	269	19.45	3.20	0.00	0.00	-7.28	DnaJ protein homolog
305	TRINITY_DN46110_c0_g1	274	19.04	3.07	0.00	0.00	-7.28	Light-independent protochlorophyllide reductase iron-sulfur ATP-binding protein
306	TRINITY_DN2347_c0_g1	269	18.63	3.06	0.00	0.00	-7.28	Protein HIR1
307	TRINITY_DN53680_c0_g1	292	19.05	2.88	0.00	0.00	-7.28	60S ribosomal protein L26
308	TRINITY_DN52006_c0_g1	270	19.14	3.14	0.00	0.00	-7.28	Chaperone protein dnaJ 3
309	TRINITY_DN45481_c0_g1	288	19.11	2.93	0.00	0.00	-7.28	Protein SMAX1-LIKE 3
310	TRINITY_DN37646_c0_g1	368	19.10	2.29	0.00	0.00	-7.28	Calcium-transporting ATPase 12, plasma membrane-type
311	TRINITY_DN23091_c0_g1	521	19.23	1.63	0.00	0.00	-7.28	Putative ribonuclease H protein At1g65750
312	TRINITY_DN55150_c0_g1	596	18.85	1.40	0.00	0.00	-7.28	Light-independent protochlorophyllide reductase iron-sulfur ATP-binding protein
313	TRINITY_DN54687_c0_g1	525	19.18	1.62	0.00	0.00	-7.28	60S ribosomal protein L5
314	TRINITY_DN43328_c0_g1	540	19.25	1.58	0.00	0.00	-7.28	Elongation factor 1-gamma
315	TRINITY_DN7847_c0_g2	851	19.24	1.00	0.00	0.00	-7.28	Profilin-2
316	TRINITY_DN5599_c0_g1	294	19.37	2.91	0.00	0.00	-7.28	Telomerase reverse transcriptase
317	TRINITY_DN17880_c0_g1	336	18.63	2.45	0.00	0.00	-7.28	Tetraspanin-19
318	TRINITY_DN30677_c0_g2	1208	161.19	5.90	0.82	0.03	-7.20	Mitochondrial carrier protein MTM1
319	TRINITY_DN23405_c0_g1	286	18.34	2.84	0.00	0.00	-7.20	Probable 2-methylcitrate dehydratase
320	TRINITY_DN46939_c0_g1	309	17.82	2.55	0.00	0.00	-7.20	Protein-lysine 6-oxidase
321	TRINITY_DN28494_c0_g1	543	17.50	1.43	0.00	0.00	-7.20	Light-regulated protein
322	TRINITY_DN31241_c1_g1	279	18.24	2.89	0.00	0.00	-7.20	MAG2-interacting protein 2
323	TRINITY_DN56424_c0_g1	352	18.45	2.32	0.00	0.00	-7.20	Chlorophyll a-b binding protein 6A, chloroplastic
324	TRINITY_DN57679_c0_g1	321	18.18	2.50	0.00	0.00	-7.20	Keratin, type I cytoskeletal 47 kDa
325	TRINITY_DN38341_c0_g1	478	17.81	1.65	0.00	0.00	-7.20	Cytochrome c oxidase subunit 6B1

*logFC: the logarithm to base 2 of fold change (Salt/Control)

**TPM: transcripts per million

Supplementary Table 1. (Cont) Annotation profile of DEG in ice plant seedlings treated with 200 mM NaCl (|FC|>4, FDR<0.001)

No.	Transcript ID	Length	Control reads	Control reads (TPM**)	Salt reads	Salt reads (TPM)	logFC*	Annotation
326	TRINITY_DN37024_c0_g1	319	17.63	2.44	0.00	0.00	-7.20	DNA-directed RNA polymerase subunit beta'
327	TRINITY_DN9090_c0_g1	329	18.48	2.48	0.00	0.00	-7.20	Transposon Tf2-11 polyprotein
328	TRINITY_DN53710_c0_g1	306	17.53	2.53	0.00	0.00	-7.20	YTH domain-containing family protein 2
329	TRINITY_DN41989_c0_g1	441	18.35	1.84	0.00	0.00	-7.20	Heat shock protein 78, mitochondrial
330	TRINITY_DN30065_c0_g1	548	17.96	1.45	0.00	0.00	-7.20	Nijmegen breakage syndrome 1 protein
331	TRINITY_DN51433_c0_g1	294	17.97	2.70	0.00	0.00	-7.20	Remorin
332	TRINITY_DN41592_c0_g1	348	17.77	2.26	0.00	0.00	-7.20	Nicotinamide riboside kinase 2
333	TRINITY_DN29268_c0_g1	324	17.73	2.42	0.00	0.00	-7.20	Protein CHROMOSOME TRANSMISSION FIDELITY 7
334	TRINITY_DN22922_c0_g1	722	18.31	1.12	0.00	0.00	-7.20	Elongation factor 1-alpha
335	TRINITY_DN15206_c0_g1	276	18.23	2.92	0.00	0.00	-7.20	DELLA protein RGA2
336	TRINITY_DN51744_c0_g1	307	17.65	2.54	0.00	0.00	-7.20	Ketol-acid reductoisomerase, chloroplastic
337	TRINITY_DN37261_c0_g1	337	18.35	2.41	0.00	0.00	-7.20	Protein FAM50A
338	TRINITY_DN22886_c0_g2	346	17.50	2.24	0.00	0.00	-7.20	DnaJ protein homolog
339	TRINITY_DN42572_c0_g1	378	18.08	2.12	0.00	0.00	-7.20	Purine nucleoside phosphorylase
340	TRINITY_DN29024_c0_g2	846	17.58	0.92	0.00	0.00	-7.20	Probable indole-3-pyruvate monooxygenase YUCCA9
341	TRINITY_DN1062_c0_g1	456	17.92	1.74	0.00	0.00	-7.20	Annexin A2
342	TRINITY_DN55187_c0_g1	313	17.10	2.42	0.00	0.00	-7.12	Glycine dehydrogenase
343	TRINITY_DN50048_c0_g1	310	17.05	2.43	0.00	0.00	-7.12	CTD kinase subunit alpha
344	TRINITY_DN46258_c0_g1	341	16.61	2.15	0.00	0.00	-7.12	V-type proton ATPase catalytic subunit A
345	TRINITY_DN27843_c0_g3	1437	16.52	0.51	0.00	0.00	-7.12	Oxygen-independent coproporphyrinogen-III oxidase-like protein sll1917
346	TRINITY_DN49367_c0_g1	279	17.40	2.76	0.00	0.00	-7.12	DNA-directed RNA polymerase I subunit RPA190
347	TRINITY_DN49196_c0_g1	285	17.22	2.67	0.00	0.00	-7.12	Retrovirus-related Pol polyprotein
348	TRINITY_DN48197_c0_g1	407	17.37	1.89	0.00	0.00	-7.12	60S ribosomal protein L30
349	TRINITY_DN52389_c0_g1	273	17.48	2.83	0.00	0.00	-7.12	Cystathionine gamma-synthase 1, chloroplastic
350	TRINITY_DN56550_c0_g1	267	17.48	2.90	0.00	0.00	-7.12	60S ribosomal protein L14-1

*logFC: the logarithm to base 2 of fold change (Salt/Control)

**TPM: transcripts per million

Supplementary Table 1. (Cont) Annotation profile of DEG in ice plant seedlings treated with 200 mM NaCl (|FC|>4, FDR<0.001)

No.	Transcript ID	Length	Control reads	Control reads (TPM**)	Salt reads	Salt reads (TPM)	logFC*	Annotation
351	TRINITY_DN43844_c0_g1	471	17.41	1.63	0.00	0.00	-7.12	Glycine-rich RNA-binding protein 3, mitochondrial
352	TRINITY_DN46206_c0_g1	279	16.51	2.62	0.00	0.00	-7.12	Large subunit GTPase 1 homolog
353	TRINITY_DN30590_c0_g2	574	17.14	1.32	0.00	0.00	-7.12	Pathogenesis-related protein 1
354	TRINITY_DN42393_c0_g1	377	16.69	1.96	0.00	0.00	-7.12	Lipocalin
355	TRINITY_DN36885_c0_g1	384	16.60	1.91	0.00	0.00	-7.12	Cytochrome P450 94A2
356	TRINITY_DN361_c0_g2	377	16.72	1.96	0.00	0.00	-7.12	Ribulose bisphosphate carboxylase small chain, chloroplastic
357	TRINITY_DN24799_c0_g1	405	17.03	1.86	0.00	0.00	-7.12	Alcohol dehydrogenase-like 7
358	TRINITY_DN8756_c0_g1	603	17.48	1.28	0.00	0.00	-7.12	60S ribosomal protein L7
359	TRINITY_DN28966_c0_g2	967	16.77	0.77	0.00	0.00	-7.12	Lys-63-specific deubiquitinase BRCC36
360	TRINITY_DN40869_c0_g1	328	16.73	2.26	0.00	0.00	-7.12	Protein Ycf2
361	TRINITY_DN40106_c0_g1	373	17.15	2.03	0.00	0.00	-7.12	Mitochondrial carrier protein RIM2
362	TRINITY_DN14710_c0_g2	285	17.26	2.68	0.00	0.00	-7.12	Alkane hydroxylase MAH1
363	TRINITY_DN54661_c0_g1	543	16.77	1.37	0.00	0.00	-7.12	envelope membrane protein, chloroplastic
364	TRINITY_DN41425_c0_g1	309	17.45	2.50	0.00	0.00	-7.12	Beta-glucosidase 1
365	TRINITY_DN16096_c0_g1	310	17.27	2.46	0.00	0.00	-7.12	Solute carrier family 25 member 38 homolog
366	TRINITY_DN14639_c0_g1	295	16.98	2.55	0.00	0.00	-7.12	ADP-ribosylation factor
367	TRINITY_DN57284_c0_g1	270	17.37	2.84	0.00	0.00	-7.12	Nicotinamide riboside kinase 2
368	TRINITY_DN37507_c0_g1	305	17.01	2.47	0.00	0.00	-7.12	Retrovirus-related Pol polyprotein from transposon 17.6
369	TRINITY_DN30624_c0_g1	1991	16.85	0.37	0.00	0.00	-7.12	Sesquiterpene synthase
370	TRINITY_DN3909_c0_g1	544	15.64	1.27	0.00	0.00	-7.03	NADH-ubiquinone oxidoreductase chain 5
371	TRINITY_DN27653_c1_g1	286	15.77	2.44	0.00	0.00	-7.03	Syntaxin-124
372	TRINITY_DN30057_c0_g1	282	15.70	2.46	0.00	0.00	-7.03	Pentatricopeptide repeat-containing protein At5g66500, mitochondrial
373	TRINITY_DN42253_c0_g1	302	16.45	2.41	0.00	0.00	-7.03	Guanine nucleotide-binding protein subunit beta-2-like 1
374	TRINITY_DN48160_c0_g1	408	16.40	1.78	0.00	0.00	-7.03	Ribulose bisphosphate carboxylase large chain
375	TRINITY_DN48131_c0_g1	424	15.58	1.62	0.00	0.00	-7.03	Cytochrome P450 71A1

*logFC: the logarithm to base 2 of fold change (Salt/Control)

**TPM: transcripts per million

Supplementary Table 1. (Cont) Annotation profile of DEG in ice plant seedlings treated with 200 mM NaCl (|FC|>4, FDR<0.001)

No.	Transcript ID	Length	Control reads	Control reads (TPM**)	Salt reads	Salt reads (TPM)	logFC*	Annotation
376	TRINITY_DN25596_c1_g2	1057	16.32	0.68	0.09	0.00	-7.03	Cysteine-rich receptor-like protein kinase 25
377	TRINITY_DN50401_c0_g1	360	16.24	2.00	0.00	0.00	-7.03	Anaphase-promoting complex subunit 5
378	TRINITY_DN53827_c0_g1	437	16.35	1.65	0.00	0.00	-7.03	Complex I intermediate-associated protein 30, mitochondrial
379	TRINITY_DN39341_c0_g1	362	16.24	1.98	0.00	0.00	-7.03	Alanine--glyoxylate aminotransferase 1
380	TRINITY_DN26170_c1_g1	284	15.88	2.47	0.00	0.00	-7.03	78 kDa glucose-regulated protein homolog
381	TRINITY_DN16664_c0_g1	333	16.01	2.13	0.00	0.00	-7.03	Putative protein disulfide-isomerase DDB_G0275025
382	TRINITY_DN11907_c0_g1	281	15.66	2.46	0.00	0.00	-7.03	Elongation factor 1-alpha
383	TRINITY_DN22051_c0_g1	661	16.14	1.08	0.00	0.00	-7.03	Inosine-5'-monophosphate dehydrogenase
384	TRINITY_DN1216_c0_g2	2128	15.90	0.33	0.38	0.01	-7.03	Putative pentatricopeptide repeat-containing protein At1g53330
385	TRINITY_DN49146_c0_g1	304	15.58	2.27	0.00	0.00	-7.03	60S ribosomal protein L35a
386	TRINITY_DN35013_c0_g2	702	16.28	1.03	0.00	0.00	-7.03	Methyltransferase-like protein 23
387	TRINITY_DN49756_c0_g1	340	15.55	2.02	0.00	0.00	-7.03	40S ribosomal protein S27-like
388	TRINITY_DN7733_c0_g1	456	15.74	1.53	0.00	0.00	-7.03	Ribulose bisphosphate carboxylase/oxygenase activase A, chloroplastic
389	TRINITY_DN32261_c0_g5	381	15.14	1.76	0.00	0.00	-6.94	Serine/threonine-protein kinase ATM
390	TRINITY_DN49414_c0_g1	333	14.55	1.93	0.00	0.00	-6.94	Glycerate dehydrogenase
391	TRINITY_DN30206_c0_g1	591	14.55	1.09	0.00	0.00	-6.94	Protein STRICTOSIDINE SYNTHASE-LIKE 2
392	TRINITY_DN37819_c0_g1	378	15.24	1.78	0.00	0.00	-6.94	Uncharacterized mitochondrial protein AtMg00750
393	TRINITY_DN17650_c0_g1	375	15.17	1.79	0.00	0.00	-6.94	Galactomannan galactosyltransferase 1
394	TRINITY_DN9733_c0_g1	314	14.84	2.09	0.00	0.00	-6.94	Phosphoribosylformylglycinamidine synthase
395	TRINITY_DN9976_c0_g1	312	15.33	2.17	0.00	0.00	-6.94	Cinnamoyl-CoA reductase 2
396	TRINITY_DN21836_c0_g2	303	14.62	2.13	0.00	0.00	-6.94	Ubiquitin-60S ribosomal protein L40
397	TRINITY_DN11354_c0_g2	346	15.17	1.94	0.00	0.00	-6.94	40S ribosomal protein S28
398	TRINITY_DN55833_c0_g1	279	15.26	2.42	0.00	0.00	-6.94	Dynamin-related protein 5A
399	TRINITY_DN27515_c0_g1	457	15.48	1.50	0.00	0.00	-6.94	Translationally-controlled tumor protein homolog
400	TRINITY_DN56671_c0_g1	332	15.34	2.04	0.00	0.00	-6.94	Oryzain alpha chain

*logFC: the logarithm to base 2 of fold change (Salt/Control)

**TPM: transcripts per million

Supplementary Table 1. (Cont) Annotation profile of DEG in ice plant seedlings treated with 200 mM NaCl (|FC|>4, FDR<0.001)

No.	Transcript ID	Length	Control reads	Control reads (TPM**)	Salt reads	Salt reads (TPM)	logFC*	Annotation
401	TRINITY_DN29406_c0_g2	433	14.52	1.48	0.00	0.00	-6.94	Probable carboxylesterase 7
402	TRINITY_DN30767_c0_g3	1049	15.15	0.64	0.01	0.00	-6.94	Metallothiol transferase FosB
403	TRINITY_DN25637_c0_g2	289	14.86	2.27	0.00	0.00	-6.94	65-kDa microtubule-associated protein 5
404	TRINITY_DN39589_c0_g1	277	14.60	2.33	0.00	0.00	-6.94	Succinate dehydrogenase
405	TRINITY_DN3435_c0_g1	723	15.03	0.92	0.00	0.00	-6.94	Elongation factor 2
406	TRINITY_DN39784_c0_g1	314	15.00	2.11	0.00	0.00	-6.94	Photosystem I reaction center subunit XI, chloroplastic
407	TRINITY_DN48207_c0_g5	796	14.78	0.82	0.47	0.02	-6.94	DNA-directed RNA polymerases II, IV and V subunit 11
408	TRINITY_DN25368_c0_g1	557	15.43	1.23	0.00	0.00	-6.94	60S ribosomal protein L13
409	TRINITY_DN24567_c1_g1	325	14.54	1.98	0.00	0.00	-6.94	Transaldolase
410	TRINITY_DN55679_c0_g1	324	15.19	2.07	0.00	0.00	-6.94	Photosystem II CP43 reaction center protein
411	TRINITY_DN46172_c0_g1	365	15.32	1.86	0.00	0.00	-6.94	Cysteine proteinase 3
412	TRINITY_DN9486_c0_g1	326	14.79	2.01	0.00	0.00	-6.94	Mannose-specific lectin
413	TRINITY_DN24105_c1_g1	464	14.83	1.41	0.00	0.00	-6.94	Wall-associated receptor kinase 2
414	TRINITY_DN54743_c0_g1	549	15.00	1.21	0.00	0.00	-6.94	Serine--glyoxylate aminotransferase
415	TRINITY_DN30894_c0_g1	966	120.35	5.51	0.88	0.04	-6.78	SH3 domain-containing protein PJ696.02
416	TRINITY_DN24850_c1_g1	1104	90.23	3.61	0.87	0.03	-6.36	Scarecrow-like protein 28
417	TRINITY_DN52832_c0_g1	1434	1659.86	51.18	22.11	0.63	-6.27	Serine carboxypeptidase-like 18
418	TRINITY_DN27916_c0_g1	1505	207.02	6.08	2.92	0.08	-6.09	E3 ubiquitin-protein ligase SIS3
419	TRINITY_DN33560_c1_g4	1089	51.60	2.10	1.00	0.04	-5.57	Pentatricopeptide repeat-containing protein At3g16010
420	TRINITY_DN16179_c0_g2	2228	90.42	1.79	1.73	0.03	-5.45	Arogenate dehydratase/prephenate dehydratase 1, chloroplastic
421	TRINITY_DN24685_c0_g1	1248	128.34	4.55	3.20	0.10	-5.40	Violaxanthin de-epoxidase, chloroplastic
422	TRINITY_DN31130_c0_g2	3289	45.34	0.61	1.04	0.01	-5.36	Pentatricopeptide repeat-containing protein At4g20090
423	TRINITY_DN32304_c0_g1	792	469.33	26.20	12.29	0.63	-5.31	UDP-glycosyltransferase 79B30
424	TRINITY_DN22647_c0_g2	1146	41.57	1.60	1.36	0.05	-5.27	Increased DNA methylation 3
425	TRINITY_DN33665_c0_g2	659	77.18	5.18	1.84	0.11	-5.22	Autophagy-related protein 18b

*logFC: the logarithm to base 2 of fold change (Salt/Control)

**TPM: transcripts per million

Supplementary Table 1. (Cont) Annotation profile of DEG in ice plant seedlings treated with 200 mM NaCl (|FC|>4, FDR<0.001)

No.	Transcript ID	Length	Control reads	Control reads (TPM**)	Salt reads	Salt reads (TPM)	logFC*	Annotation
426	TRINITY_DN35118_c0_g2	1788	37.61	0.93	0.57	0.01	-5.12	Protein TIC 62, chloroplastic
427	TRINITY_DN52959_c0_g1	778	36.42	2.07	1.39	0.07	-5.04	Alpha-glucosides permease MPH2
428	TRINITY_DN31733_c0_g2	1775	67.49	1.68	2.32	0.05	-5.02	Serine/threonine-protein kinase HT1
429	TRINITY_DN31457_c0_g4	1032	34.97	1.50	1.29	0.05	-5.00	Sulfhydryl oxidase 2
430	TRINITY_DN30088_c0_g3	1418	34.22	1.07	1.37	0.04	-4.96	Phosphate transporter PHO1 homolog 1
431	TRINITY_DN33166_c0_g1	724	32.85	2.01	1.41	0.08	-4.92	Receptor protein kinase CLAVATA1
432	TRINITY_DN25039_c0_g1	1326	31.05	1.04	0.55	0.02	-4.83	Anaphase-promoting complex subunit 5
433	TRINITY_DN25235_c1_g1	797	31.39	1.74	1.38	0.07	-4.83	Pentatricopeptide repeat-containing protein At2g02750
434	TRINITY_DN31872_c0_g1	1985	56.74	1.26	2.14	0.04	-4.79	Aldehyde dehydrogenase family 2 member B7, mitochondrial
435	TRINITY_DN35028_c6_g6	1937	29.72	0.68	1.43	0.03	-4.78	Phosphoribosylaminoimidazole-succinocarboxamide synthase, chloroplastic
436	TRINITY_DN23181_c0_g1	995	30.32	1.35	1.36	0.06	-4.78	Thiamine thiazole synthase
437	TRINITY_DN1983_c0_g2	1369	29.02	0.94	0.60	0.02	-4.73	UDP-glycosyltransferase 90A1
438	TRINITY_DN31900_c0_g2	1605	52.79	1.45	2.08	0.05	-4.68	Homeobox-leucine zipper protein HAT5
439	TRINITY_DN26603_c0_g3	1223	28.02	1.01	1.19	0.04	-4.68	Multiple organellar RNA editing factor 3, mitochondrial
440	TRINITY_DN32648_c0_g2	2835	51.56	0.80	1.53	0.02	-4.66	Quinolinate synthase, chloroplastic
441	TRINITY_DN33526_c0_g3	829	27.38	1.46	1.39	0.07	-4.63	Probable methyltransferase PMT2
442	TRINITY_DN38354_c0_g1	636	51.36	3.57	1.67	0.11	-4.63	Transcription factor bHLH84
443	TRINITY_DN33496_c0_g3	1113	26.21	1.04	1.45	0.05	-4.58	7-deoxyloganetin glucosyltransferase
444	TRINITY_DN30814_c0_g1	3388	26.32	0.34	0.57	0.01	-4.58	Pentatricopeptide repeat-containing protein At2g42920, chloroplastic
445	TRINITY_DN34432_c0_g1	1093	25.62	1.04	1.20	0.04	-4.58	Phosphatidylinositol 3-kinase, nodule isoform
446	TRINITY_DN26857_c0_g1	658	24.67	1.66	0.75	0.05	-4.52	Probable sodium/metabolite cotransporter BASS3, chloroplastic
447	TRINITY_DN4840_c0_g1	1590	202.70	5.64	9.34	0.24	-4.52	Cysteine--tRNA ligase, chloroplastic/mitochondrial
448	TRINITY_DN30163_c0_g1	1208	90.22	3.30	3.74	0.13	-4.49	Phosphoenolpyruvate carboxykinase
449	TRINITY_DN32632_c1_g1	411	23.69	2.55	1.09	0.11	-4.46	Jasmonic acid-amido synthetase JAR1
450	TRINITY_DN34819_c0_g4	269	127.29	20.92	5.80	0.88	-4.42	NADP-dependent alkenal double bond reductase P2

*logFC: the logarithm to base 2 of fold change (Salt/Control)

**TPM: transcripts per million

Supplementary Table 1. (Cont) Annotation profile of DEG in ice plant seedlings treated with 200 mM NaCl (|FC|>4, FDR<0.001)

No.	Transcript ID	Length	Control reads	Control reads (TPM**)	Salt reads	Salt reads (TPM)	logFC*	Annotation
451	TRINITY_DN26857_c0_g3	642	207.84	14.32	9.81	0.62	-4.40	Probable sodium/metabolite cotransporter BASS3, chloroplastic
452	TRINITY_DN27359_c0_g1	854	23.13	1.20	1.39	0.07	-4.40	Heavy metal-associated isoprenylated plant protein 21
453	TRINITY_DN30685_c0_g1	1233	42.00	1.51	1.82	0.06	-4.35	tRNA (adenine(37)-N6)-methyltransferase
454	TRINITY_DN13468_c0_g1	481	41.37	3.80	2.10	0.18	-4.31	Magnesium-protoporphyrin IX monomethyl ester
455	TRINITY_DN28828_c0_g2	483	40.17	3.68	1.87	0.16	-4.28	F-actin-capping protein subunit alpha
456	TRINITY_DN21730_c0_g1	1173	19.53	0.74	1.27	0.04	-4.20	Protein SPA1-RELATED 2
457	TRINITY_DN33428_c0_g1	1519	19.63	0.57	1.08	0.03	-4.20	Multicopper oxidase LPR1
458	TRINITY_DN32807_c0_g1	1115	54.52	2.16	2.53	0.09	-4.18	Probable starch synthase 4, chloroplastic/amyloplastic
459	TRINITY_DN19258_c0_g2	371	175.49	20.92	10.14	1.11	-4.15	PHD finger protein ALFIN-LIKE 5
460	TRINITY_DN29792_c0_g2	2242	19.46	0.38	0.93	0.02	-4.13	DNA (cytosine-5)-methyltransferase 1B
461	TRINITY_DN29177_c0_g2	917	35.25	1.70	2.28	0.10	-4.09	Putative transcription factor bHLH107
462	TRINITY_DN31038_c0_g1	838	18.49	0.98	0.61	0.03	-4.05	Protein RALF-like 33
463	TRINITY_DN32611_c0_g3	1674	17.72	0.47	1.16	0.03	-4.05	DNA repair protein RAD4
464	TRINITY_DN31840_c0_g2	485	17.89	1.63	1.23	0.10	-4.05	Peroxidase 45
465	TRINITY_DN32485_c0_g2	494.35	33.79	3.18	2.31	0.20	-4.05	Ubiquitin-60S ribosomal protein L40-2
466	TRINITY_DN30466_c0_g2	668	113.32	7.50	6.52	0.40	-4.03	Dynein light chain, cytoplasmic
467	TRINITY_DN33392_c0_g2	1696	488.53	12.74	31.22	0.75	-4.01	Ribose-phosphate pyrophosphokinase 3, mitochondrial
468	TRINITY_DN6060_c0_g2	1152	33.39	1.28	1.85	0.07	-4.00	snRNA-activating protein complex subunit
469	TRINITY_DN27523_c0_g2	633	32.51	2.27	1.63	0.10	-4.00	Protein MALE DISCOVERER 2
470	TRINITY_DN42877_c0_g3	1282	77.82	2.68	4.99	0.16	-3.97	Mediator-associated protein 2
471	TRINITY_DN43114_c0_g1	428	31.85	3.29	2.36	0.22	-3.96	Acyl-coenzyme A oxidase 1
472	TRINITY_DN31761_c0_g5	1837	32.32	0.78	2.35	0.05	-3.96	E3 ubiquitin-protein ligase ORTHRUS 2
473	TRINITY_DN33441_c0_g3	2457	31.17	0.56	2.34	0.04	-3.91	Pumilio homolog 5
474	TRINITY_DN44038_c0_g1	765	30.56	1.77	1.55	0.08	-3.91	Formate dehydrogenase
475	TRINITY_DN11817_c1_g1	794	43.66	2.43	3.06	0.16	-3.86	Succinate/fumarate mitochondrial transporter

*logFC: the logarithm to base 2 of fold change (Salt/Control)

**TPM: transcripts per million

Supplementary Table 1. (Cont) Annotation profile of DEG in ice plant seedlings treated with 200 mM NaCl (|FC|>4, FDR<0.001)

No.	Transcript ID	Length	Control reads	Control reads (TPM**)	Salt reads	Salt reads (TPM)	logFC*	Annotation
476	TRINITY_DN2366_c0_g2	2386	44.17	0.82	3.32	0.06	-3.86	Putative ribonuclease H protein At1g65750
477	TRINITY_DN23227_c0_g1	1759	43.40	1.09	3.11	0.07	-3.83	Protein ALP1-like
478	TRINITY_DN29749_c0_g1	552	28.88	2.31	1.74	0.13	-3.82	Growth-regulating factor 4
479	TRINITY_DN26938_c0_g2	604	69.61	5.10	4.96	0.33	-3.81	Probable inorganic phosphate transporter 1-2
480	TRINITY_DN37611_c0_g1	317	42.30	5.90	3.39	0.44	-3.79	Isocitrate lyase
481	TRINITY_DN25245_c0_g2	1305	68.09	2.31	5.36	0.17	-3.77	Syntaxin-51
482	TRINITY_DN27490_c0_g2	684	28.35	1.83	1.71	0.10	-3.77	Glycerophosphodiester phosphodiesterase GDPDL4
483	TRINITY_DN27245_c0_g1	613	28.45	2.05	1.82	0.12	-3.77	4-coumarate--CoA ligase-like 3
484	TRINITY_DN34816_c0_g1	558	41.25	3.27	3.38	0.25	-3.76	Caffeic acid 3-O-methyltransferase 1
485	TRINITY_DN33295_c0_g1	677	644.45	42.09	49.23	2.96	-3.75	Probable plastid-lipid-associated protein 13, chloroplastic
486	TRINITY_DN53510_c0_g1	513	27.26	2.35	1.96	0.16	-3.71	Zinc finger BED domain-containing protein RICESLEEPER 2
487	TRINITY_DN33164_c0_g2	1750	39.06	0.99	2.75	0.06	-3.69	Brefeldin A-inhibited guanine nucleotide-exchange protein 1
488	TRINITY_DN30334_c0_g1	1061	39.36	1.64	2.56	0.10	-3.69	Protection of telomeres protein 1b
489	TRINITY_DN35183_c0_g3	494	26.26	2.35	1.89	0.16	-3.66	Protein TRIGALACTOSYLDIACYLGLYCEROL 2, chloroplastic
490	TRINITY_DN28545_c0_g1	373	26.35	3.12	2.48	0.27	-3.66	Septin and tuftelin-interacting protein 1 homolog 1
491	TRINITY_DN30814_c0_g3	2760	26.30	0.42	2.08	0.03	-3.66	Pentatricopeptide repeat-containing protein At2g42920, chloroplastic
492	TRINITY_DN29676_c0_g12	1353	26.16	0.86	2.43	0.07	-3.66	Thioredoxin M2, chloroplastic
493	TRINITY_DN33724_c0_g1	1210	49.63	1.81	3.64	0.12	-3.64	Pentatricopeptide repeat-containing protein At5g61400
494	TRINITY_DN4363_c0_g1	1099	194.19	7.81	16.08	0.60	-3.63	Agamous-like MADS-box protein AGL80
495	TRINITY_DN33691_c0_g1	2580.91	84.89	1.46	7.38	0.12	-3.62	Galactan beta-1,4-galactosyltransferase GALS3
496	TRINITY_DN34736_c0_g2	633	61.06	4.27	5.14	0.33	-3.62	Heparanase-like protein 3
497	TRINITY_DN31244_c0_g1	991	49.10	2.19	3.70	0.15	-3.61	ANTL2_ARATH
498	TRINITY_DN25739_c0_g1	290	48.96	7.47	4.01	0.56	-3.61	Retrovirus-related Pol polyprotein from transposon TNT 1-94
499	TRINITY_DN2960_c0_g1	671	24.92	1.64	1.78	0.11	-3.60	L-ornithine N(5)-monooxygenase
500	TRINITY_DN38104_c0_g1	347	35.53	4.53	2.99	0.35	-3.57	GTP-binding protein rhb1

*logFC: the logarithm to base 2 of fold change (Salt/Control)

**TPM: transcripts per million

Supplementary Table 1. (Cont) Annotation profile of DEG in ice plant seedlings treated with 200 mM NaCl (|FC|>4, FDR<0.001)

No.	Transcript ID	Length	Control reads	Control reads (TPM**)	Salt reads	Salt reads (TPM)	logFC*	Annotation
501	TRINITY_DN41627_c0_g1	1067	35.53	1.47	2.70	0.10	-3.57	Aspartic protease
502	TRINITY_DN13680_c0_g1	530	23.67	1.98	2.08	0.16	-3.54	Cell division control protein 48 homolog C
503	TRINITY_DN16873_c0_g1	439	23.77	2.39	2.08	0.19	-3.54	5-oxoprolinase
504	TRINITY_DN32888_c1_g1	1088.74	3060.33	122.67	272.22	10.30	-3.53	Metallothiol transferase FosB
505	TRINITY_DN31274_c0_g1	534	46.13	3.82	3.94	0.30	-3.52	Probable apyrase 6
506	TRINITY_DN1446_c0_g1	3036	279.22	4.07	24.83	0.33	-3.51	Probable galactinol--sucrose galactosyltransferase 1
507	TRINITY_DN13329_c0_g1	894	100.70	4.98	8.80	0.40	-3.51	Aldehyde dehydrogenase
508	TRINITY_DN49969_c0_g1	300	44.62	6.58	4.37	0.59	-3.49	Transmembrane protein 14C
509	TRINITY_DN29448_c0_g1	2521	45.48	0.80	3.78	0.06	-3.49	Histone-lysine N-methyltransferase family member SUVH2
510	TRINITY_DN32245_c0_g3	1161	34.38	1.31	3.37	0.12	-3.49	Exonuclease 3'-5' domain-containing protein 2
511	TRINITY_DN40321_c0_g1	464	22.68	2.16	1.79	0.16	-3.48	NEDD8
512	TRINITY_DN9136_c0_g1	571	22.58	1.75	1.86	0.13	-3.48	Putative glutamine-dependent NAD(+) synthetase
513	TRINITY_DN28458_c0_g1	717	23.01	1.42	2.20	0.13	-3.48	Sucrose synthase
514	TRINITY_DN28076_c0_g1	1436	33.07	1.02	3.41	0.10	-3.45	Sodium channel modifier 1
515	TRINITY_DN22880_c2_g1	401	22.13	2.44	2.48	0.25	-3.42	Protein PIN-LIKES 1
516	TRINITY_DN26161_c2_g1	467	21.80	2.06	2.02	0.18	-3.42	Pentatricopeptide repeat-containing protein At4g35130, chloroplastic
517	TRINITY_DN29036_c3_g1	394	21.65	2.43	2.49	0.26	-3.42	DNA oxidative demethylase ALKBH2
518	TRINITY_DN13307_c0_g3	3127	52.91	0.75	4.92	0.06	-3.41	BTB/POZ domain-containing protein At3g05675
519	TRINITY_DN33915_c0_g3	589	52.70	3.96	4.89	0.34	-3.41	Protein SYM1
520	TRINITY_DN20284_c0_g1	378	31.52	3.69	3.19	0.34	-3.40	Serine/arginine-rich splicing factor 6
521	TRINITY_DN56357_c0_g1	288	80.81	12.41	7.95	1.12	-3.36	Uncharacterized protein C338.12
522	TRINITY_DN9533_c0_g1	362	31.04	3.79	2.93	0.33	-3.36	Probable inorganic phosphate transporter 1-12
523	TRINITY_DN27533_c0_g1	699	20.66	1.31	1.69	0.10	-3.35	Putative DNA glycosylase At3g47830
524	TRINITY_DN30687_c0_g1	507	20.68	1.80	1.75	0.14	-3.35	ATPase 7, plasma membrane-type
525	TRINITY_DN44103_c0_g1	462	21.21	2.03	2.09	0.18	-3.35	Probable peptide methionine sulfoxide reductase

*logFC: the logarithm to base 2 of fold change (Salt/Control)

**TPM: transcripts per million

Supplementary Table 1. (Cont) Annotation profile of DEG in ice plant seedlings treated with 200 mM NaCl (|FC|>4, FDR<0.001)

No.	Transcript ID	Length	Control reads	Control reads (TPM**)	Salt reads	Salt reads (TPM)	logFC*	Annotation
526	TRINITY_DN8414_c0_g1	397	20.75	2.31	2.31	0.24	-3.35	Geraniol 8-hydroxylase
527	TRINITY_DN31947_c2_g1	910	968.85	47.08	98.89	4.43	-3.33	Peroxidase 60
528	TRINITY_DN33634_c0_g1	1242	292.17	10.40	29.50	0.97	-3.32	Alcohol dehydrogenase 3, mitochondrial
529	TRINITY_DN22801_c0_g1	968	59.30	2.71	5.95	0.25	-3.31	UDP-glycosyltransferase 79B30
530	TRINITY_DN52872_c0_g3	684	30.02	1.94	2.91	0.17	-3.31	DEAD-box ATP-dependent RNA helicase 26
531	TRINITY_DN1472_c0_g1	765	97.12	5.61	9.67	0.51	-3.30	60S ribosomal protein L15
532	TRINITY_DN26528_c0_g1	1216	48.80	1.77	5.21	0.17	-3.30	DNA repair protein RAD51 homolog 2
533	TRINITY_DN22634_c0_g1	324	39.12	5.34	3.77	0.47	-3.29	Cytochrome P450 98A2
534	TRINITY_DN3739_c0_g2	855	66.84	3.46	7.42	0.35	-3.28	F-box protein CPR30
535	TRINITY_DN4525_c0_g1	600	113.34	8.35	12.37	0.84	-3.26	Kinesin-like protein KIN-5C
536	TRINITY_DN6338_c0_g1	1106	211.38	8.45	23.36	0.86	-3.23	Accumulation of dyads protein 2
537	TRINITY_DN24835_c1_g1	895	72.88	3.60	8.22	0.37	-3.21	Methyl-CpG-binding domain-containing protein 4
538	TRINITY_DN34813_c1_g1	1752	46.03	1.16	5.44	0.13	-3.21	F-box protein SKIP23
539	TRINITY_DN40049_c0_g1	335	37.22	4.91	4.08	0.50	-3.21	Serine carboxypeptidase-like 4
540	TRINITY_DN49219_c0_g1	364	28.12	3.42	2.87	0.32	-3.21	UPF0662 protein C30C2.08
541	TRINITY_DN47766_c0_g1	1877	9213.59	217.06	1024.87	22.25	-3.21	S-coclaurine N-methyltransferase
542	TRINITY_DN34409_c2_g3	1463	321.86	9.73	36.16	1.01	-3.20	Exosome complex component RRP4 homolog
543	TRINITY_DN54875_c0_g1	886	99.25	4.95	11.22	0.52	-3.20	Isocitrate lyase
544	TRINITY_DN22640_c0_g3	652	72.12	4.89	7.65	0.48	-3.19	Peroxidase 57
545	TRINITY_DN31475_c1_g1	825	45.42	2.43	5.29	0.26	-3.18	Expansin-like A1
546	TRINITY_DN54373_c0_g1	385	27.35	3.14	2.56	0.27	-3.16	Fe-S cluster assembly protein DRE2
547	TRINITY_DN56915_c0_g1	296	26.63	3.98	3.30	0.45	-3.16	Protein fluG
548	TRINITY_DN21432_c0_g1	889	53.29	2.65	6.01	0.28	-3.16	Bidirectional sugar transporter SWEET17
549	TRINITY_DN26477_c0_g1	1217	6059.10	220.15	701.65	23.49	-3.15	Early light-induced protein 1, chloroplastic
550	TRINITY_DN14873_c0_g2	1918	371.60	8.57	43.08	0.92	-3.15	Transcription factor bHLH144

*logFC: the logarithm to base 2 of fold change (Salt/Control)

**TPM: transcripts per million

Supplementary Table 1. (Cont) Annotation profile of DEG in ice plant seedlings treated with 200 mM NaCl (|FC|>4, FDR<0.001)

No.	Transcript ID	Length	Control reads	Control reads (TPM**)	Salt reads	Salt reads (TPM)	logFC*	Annotation
551	TRINITY_DN33063_c0_g2	469	78.11	7.36	8.99	0.78	-3.14	Ribonucleoside-diphosphate reductase large subunit
552	TRINITY_DN19996_c0_g2	800	34.85	1.93	4.44	0.23	-3.13	Oxygen-dependent coproporphyrinogen-III oxidase, chloroplastic
553	TRINITY_DN24483_c0_g1	331	26.35	3.52	3.00	0.37	-3.10	Serine/threonine-protein kinase AGC1-5
554	TRINITY_DN28690_c0_g1	580	25.95	1.98	3.40	0.24	-3.10	ABC transporter patM
555	TRINITY_DN37665_c0_g1	344	26.33	3.38	3.29	0.39	-3.10	Zinc-type alcohol dehydrogenase-like protein PB24D3.08c
556	TRINITY_DN35003_c0_g1	686	83.75	5.40	10.46	0.62	-3.10	Transcription termination factor MTEF1, chloroplastic
557	TRINITY_DN32614_c0_g1	589	82.24	6.17	10.11	0.70	-3.06	Capsid protein
558	TRINITY_DN45755_c0_g1	355	24.78	3.09	3.21	0.37	-3.05	Protein hob1
559	TRINITY_DN38319_c0_g1	325	25.42	3.46	3.28	0.41	-3.05	LINE-1 retrotransposable element ORF2 protein
560	TRINITY_DN55067_c0_g1	818	24.64	1.33	3.00	0.15	-3.05	Heat shock protein HSS1
561	TRINITY_DN31872_c0_g3	1985	32.94	0.73	4.06	0.08	-3.05	Aldehyde dehydrogenase family 2 member B7, mitochondrial
562	TRINITY_DN30537_c0_g2	496	33.34	2.97	4.47	0.37	-3.05	Putative RING-H2 finger protein ATL69
563	TRINITY_DN30910_c0_g3	1443	153.11	4.69	18.75	0.53	-3.04	Dof zinc finger protein DOF5.3
564	TRINITY_DN31130_c0_g1	3305	144.00	1.93	18.40	0.23	-3.03	Pentatricopeptide repeat-containing protein At4g20090
565	TRINITY_DN34048_c1_g1	293	55.91	8.44	7.32	1.02	-3.02	WD and tetratricopeptide repeats protein 1
566	TRINITY_DN47341_c0_g2	813	236.51	12.86	29.80	1.49	-3.02	Protein EXORDIUM
567	TRINITY_DN4046_c0_g1	1910	78.63	1.82	10.38	0.22	-3.01	Mitochondrial substrate carrier family protein B
568	TRINITY_DN22116_c0_g3	292	32.13	4.87	3.84	0.54	-3.00	FT-interacting protein 1
569	TRINITY_DN28468_c2_g1	323	32.11	4.40	4.33	0.55	-3.00	Mediator of RNA polymerase II transcription subunit 15a
570	TRINITY_DN53390_c0_g1	293	39.19	5.91	5.26	0.73	-2.97	Nicotinate-nucleotide pyrophosphorylase
571	TRINITY_DN34306_c0_g3	686	31.12	2.01	4.08	0.24	-2.96	Protein DAMAGED DNA-BINDING 2
572	TRINITY_DN12425_c0_g2	488	30.79	2.79	3.70	0.31	-2.96	Caffeoylshikimate esterase
573	TRINITY_DN32752_c0_g1	1870	248.59	5.88	33.00	0.72	-2.95	Cytochrome P450 71D10
574	TRINITY_DN25117_c0_g2	1674	59.85	1.58	8.43	0.21	-2.93	Endoglucanase
575	TRINITY_DN15349_c0_g2	1040	67.11	2.85	9.28	0.36	-2.92	Alternative oxidase, mitochondrial

*logFC: the logarithm to base 2 of fold change (Salt/Control)

**TPM: transcripts per million

Supplementary Table 1. (Cont) Annotation profile of DEG in ice plant seedlings treated with 200 mM NaCl (|FC|>4, FDR<0.001)

No.	Transcript ID	Length	Control reads	Control reads (TPM**)	Salt reads	Salt reads (TPM)	logFC*	Annotation
576	TRINITY_DN31948_c0_g1	1012	102.60	4.48	13.59	0.55	-2.91	Probable methyltransferase PMT15
577	TRINITY_DN37120_c0_g1	290	30.22	4.61	4.29	0.60	-2.91	Probable splicing factor 3B subunit 5
578	TRINITY_DN10563_c0_g2	1518	152.51	4.44	21.10	0.57	-2.90	Acetyl-coenzyme A synthetase
579	TRINITY_DN32210_c0_g6	1343	188.60	6.21	26.05	0.79	-2.90	INO80 complex subunit C
580	TRINITY_DN2096_c0_g1	584	86.39	6.54	12.10	0.84	-2.87	Remorin
581	TRINITY_DN51375_c0_g1	287	28.62	4.41	4.46	0.63	-2.86	Nicotinate phosphoribosyltransferase
582	TRINITY_DN29269_c0_g2	1230	29.09	1.05	4.33	0.14	-2.86	G-type lectin S-receptor-like serine/threonine-protein kinase SD1-13
583	TRINITY_DN39780_c0_g1	580	29.36	2.24	4.07	0.29	-2.86	5-aminolevulinate synthase, mitochondrial
584	TRINITY_DN34739_c0_g5	1265	509.19	17.80	73.33	2.36	-2.84	Probable xyloglucan endotransglucosylase/hydrolase protein 26
585	TRINITY_DN12445_c0_g9	983	112.32	5.05	16.07	0.67	-2.84	Peroxisomal membrane protein PEX14
586	TRINITY_DN6700_c0_g2	635	91.19	6.35	13.18	0.85	-2.84	30S ribosomal protein S17, chloroplastic
587	TRINITY_DN19036_c0_g1	721	63.43	3.89	8.57	0.48	-2.83	Malate synthase, glyoxysomal
588	TRINITY_DN1446_c0_g2	2907	3336.34	50.75	484.67	6.79	-2.82	Probable galactinol--sucrose galactosyltransferase 1
589	TRINITY_DN28843_c0_g4	1887	102.82	2.41	15.08	0.33	-2.81	Heterogeneous nuclear ribonucleoprotein 1
590	TRINITY_DN50017_c0_g1	316	27.95	3.91	3.67	0.47	-2.81	Fucosyltransferase 6
591	TRINITY_DN25717_c0_g1	320	28.33	3.91	3.83	0.49	-2.81	Cysteine desulfurase, mitochondrial
592	TRINITY_DN35246_c0_g1	798	53.67	2.97	7.53	0.38	-2.78	Putative cysteine-rich receptor-like protein kinase 12
593	TRINITY_DN26346_c0_g5	522	86.59	7.33	12.98	1.01	-2.77	Anaphase-promoting complex subunit 13
594	TRINITY_DN47521_c0_g1	727	26.83	1.63	3.57	0.20	-2.76	Heat shock protein 104
595	TRINITY_DN17964_c0_g1	325	26.78	3.64	3.81	0.48	-2.76	Ubiquitin carboxyl-terminal hydrolase 21
596	TRINITY_DN30859_c0_g2	613	39.80	2.87	5.86	0.39	-2.75	Auxin-responsive protein SAUR71
597	TRINITY_DN32716_c0_g3	551	124.64	10.00	18.60	1.38	-2.75	Putative ribonuclease H protein At1g65750
598	TRINITY_DN8632_c0_g1	2156	70.72	1.45	10.65	0.20	-2.72	Lysine-specific histone demethylase 1 homolog 1
599	TRINITY_DN34518_c0_g1	544	198.22	16.11	31.29	2.34	-2.71	Peroxisome biogenesis protein 16
600	TRINITY_DN1639_c0_g1	1212	64.12	2.34	9.75	0.33	-2.70	Autophagy-related protein 101

*logFC: the logarithm to base 2 of fold change (Salt/Control)

**TPM: transcripts per million

Supplementary Table 1. (Cont) Annotation profile of DEG in ice plant seedlings treated with 200 mM NaCl (|FC|>4, FDR<0.001)

No.	Transcript ID	Length	Control reads	Control reads (TPM**)	Salt reads	Salt reads (TPM)	logFC*	Annotation
601	TRINITY_DN1481_c0_g1	880	89.19	4.48	14.32	0.66	-2.70	Auxin-binding protein T85
602	TRINITY_DN27406_c0_g1	837	32.42	1.71	5.44	0.26	-2.69	Pentatricopeptide repeat-containing protein DOT4, chloroplastic
603	TRINITY_DN30197_c0_g1	1545.32	528.37	15.01	84.81	2.29	-2.67	GDSL esterase/lipase At4g16230
604	TRINITY_DN32441_c0_g1	3998	129.83	1.44	21.11	0.22	-2.66	Nodulation protein H
605	TRINITY_DN28837_c0_g2	997	92.98	4.12	14.85	0.61	-2.66	Endoplasmic reticulum oxidoreductin-1
606	TRINITY_DN30623_c0_g1	821	31.26	1.68	4.57	0.23	-2.64	Transcription factor bHLH3
607	TRINITY_DN22345_c0_g1	651	31.10	2.11	4.65	0.29	-2.64	Aluminum-activated malate transporter 8
608	TRINITY_DN20093_c0_g1	1462	1112.72	33.65	184.33	5.14	-2.64	Peroxidase 60
609	TRINITY_DN34739_c0_g1	1344	556.11	18.30	91.93	2.79	-2.63	Probable xyloglucan endotransglucosylase/hydrolase protein 26
610	TRINITY_DN22640_c0_g2	846	298.79	15.62	50.10	2.41	-2.62	Peroxidase 60
611	TRINITY_DN32304_c0_g2	1806	114.49	2.80	19.30	0.44	-2.62	UDP-glycosyltransferase 79B30
612	TRINITY_DN35764_c0_g3	1445	154.62	4.73	25.61	0.72	-2.61	ER membrane protein complex subunit 10
613	TRINITY_DN26667_c0_g2	1572	53.79	1.51	9.07	0.24	-2.61	Caffeoylshikimate esterase
614	TRINITY_DN39844_c0_g1	475	54.26	5.05	8.87	0.76	-2.61	Fatty acid-binding protein
615	TRINITY_DN33182_c0_g2	2774	193.77	3.09	33.48	0.49	-2.59	Zinc phosphodiesterase ELAC protein 2
616	TRINITY_DN32953_c3_g1	366	100.24	12.11	17.35	1.93	-2.59	Aluminum-activated malate transporter 7
617	TRINITY_DN34537_c0_g2	671	52.77	3.48	9.12	0.55	-2.58	Probable flavin-containing monooxygenase 1
618	TRINITY_DN12958_c0_g2	1553	92.66	2.64	15.85	0.42	-2.57	Exosome complex component RRP42
619	TRINITY_DN31557_c0_g1	953	41.23	1.91	7.38	0.32	-2.57	3-isopropylmalate dehydratase large subunit, chloroplastic
620	TRINITY_DN25235_c0_g2	2150	63.79	1.31	10.87	0.21	-2.57	Pentatricopeptide repeat-containing protein At2g02750
621	TRINITY_DN53093_c0_g1	942	57.61	2.70	10.18	0.44	-2.56	High-affinity glucose transporter ght2
622	TRINITY_DN27310_c0_g2	1110	35.17	1.40	6.43	0.24	-2.56	Pentatricopeptide repeat-containing protein At2g28050
623	TRINITY_DN57347_c0_g1	278	28.72	4.57	5.03	0.74	-2.55	ABC transporter C family member 10
624	TRINITY_DN43639_c0_g1	1150	95.88	3.69	16.95	0.60	-2.53	Adducin-related protein C1289.14
625	TRINITY_DN23059_c0_g1	1331	162.50	5.40	29.21	0.89	-2.52	Basic 7S globulin

*logFC: the logarithm to base 2 of fold change (Salt/Control)

**TPM: transcripts per million

Supplementary Table 1. (Cont) Annotation profile of DEG in ice plant seedlings treated with 200 mM NaCl (|FC|>4, FDR<0.001)

No.	Transcript ID	Length	Control reads	Control reads (TPM**)	Salt reads	Salt reads (TPM)	logFC*	Annotation
626	TRINITY_DN47640_c0_g1	1556	1826.06	51.89	335.33	8.78	-2.49	Basic 7S globulin 2
627	TRINITY_DN38206_c0_g1	1542	97.72	2.80	18.37	0.49	-2.48	Putative ribonuclease H protein At1g65750
628	TRINITY_DN33641_c0_g2	1458	81.43	2.47	14.97	0.42	-2.46	ATP-dependent RNA helicase DEAH11, chloroplastic
629	TRINITY_DN9102_c0_g5	1567	186.79	5.27	35.19	0.92	-2.45	Dolichyl-diphosphooligosaccharide--protein glycosyltransferase subunit 1B
630	TRINITY_DN37807_c0_g1	648	42.87	2.93	8.33	0.52	-2.45	Hit family protein 1
631	TRINITY_DN16116_c0_g4	829	3567.74	190.30	676.27	33.24	-2.44	60S ribosomal protein L23
632	TRINITY_DN29741_c0_g1	1068	89.90	3.72	16.68	0.64	-2.44	GDSL esterase/lipase At5g03980
633	TRINITY_DN25387_c0_g2	1660	381.89	10.17	74.45	1.83	-2.41	Probable S-sulfocysteine synthase, chloroplastic
634	TRINITY_DN49064_c0_g1	712	52.39	3.25	9.55	0.55	-2.40	Fatty acyl-CoA reductase 1
635	TRINITY_DN42322_c0_g1	718	93.48	5.76	17.79	1.01	-2.40	Peroxidase 60
636	TRINITY_DN15965_c0_g2	1323	35.57	1.19	7.01	0.22	-2.38	Peroxidase 5
637	TRINITY_DN6334_c0_g1	786	35.63	2.00	7.07	0.37	-2.38	2-isopropylmalate synthase
638	TRINITY_DN55839_c0_g1	286	60.63	9.37	12.35	1.76	-2.37	NADH dehydrogenase
639	TRINITY_DN18144_c0_g2	1477	329.16	9.85	65.97	1.82	-2.36	UDP-glycosyltransferase 74E2
640	TRINITY_DN4521_c0_g1	961	85.31	3.93	17.29	0.73	-2.35	Auxin-induced protein 6B
641	TRINITY_DN30998_c0_g1	1503.08	209.10	6.16	42.30	1.13	-2.35	F-box protein At5g49610
642	TRINITY_DN35179_c2_g3	2037	2654.79	57.63	540.40	10.81	-2.34	Probable sesquiterpene synthase
643	TRINITY_DN31697_c0_g2	1054	63.54	2.67	13.38	0.52	-2.33	Protein ALTERED XYLOGLUCAN 4-like
644	TRINITY_DN9761_c0_g2	787	88.47	4.97	18.12	0.94	-2.32	Zinc finger Ran-binding domain-containing protein 2
645	TRINITY_DN21278_c0_g1	500	83.41	7.38	16.93	1.38	-2.32	Chalcone synthase
646	TRINITY_DN8544_c0_g2	402	49.25	5.42	10.11	1.03	-2.32	Uncharacterized exonuclease domain-containing protein At3g15140
647	TRINITY_DN8150_c0_g1	861	77.98	4.00	16.22	0.77	-2.32	Fumarate reductase
648	TRINITY_DN28527_c0_g2	1894	58.33	1.36	12.00	0.26	-2.30	Formin-like protein 5
649	TRINITY_DN23479_c1_g1	332	34.26	4.56	7.40	0.91	-2.30	Pentatricopeptide repeat-containing protein At2g17210
650	TRINITY_DN34843_c1_g1	1985	2156.05	48.03	452.87	9.30	-2.29	Basic 7S globulin

*logFC: the logarithm to base 2 of fold change (Salt/Control)

**TPM: transcripts per million

Supplementary Table 1. (Cont) Annotation profile of DEG in ice plant seedlings treated with 200 mM NaCl (|FC|>4, FDR<0.001)

No.	Transcript ID	Length	Control reads	Control reads (TPM**)	Salt reads	Salt reads (TPM)	logFC*	Annotation
651	TRINITY_DN8936_c0_g2	1119	119.35	4.72	25.13	0.92	-2.29	Beta-carotene isomerase D27, chloroplastic
652	TRINITY_DN18144_c0_g1	1716	1211.23	31.21	256.17	6.08	-2.28	UDP-glycosyltransferase 74F2
653	TRINITY_DN33072_c1_g1	284	42.73	6.65	8.83	1.27	-2.28	Poly(A)-specific ribonuclease PARN-like
654	TRINITY_DN34851_c0_g1	457	37.83	3.66	7.83	0.70	-2.27	2-succinylbenzoate--CoA ligase, chloroplastic/peroxisomal
655	TRINITY_DN29054_c0_g1	728	37.82	2.30	7.80	0.44	-2.27	Pentatricopeptide repeat-containing protein At3g29230
656	TRINITY_DN17606_c0_g3	1008	535.30	23.48	113.51	4.59	-2.27	CBS domain-containing protein CBSX6
657	TRINITY_DN34739_c0_g4	496	303.93	27.10	65.39	5.37	-2.26	Probable xyloglucan endotransglucosylase/hydrolase protein 26
658	TRINITY_DN13527_c0_g1	920	153.85	7.39	32.87	1.46	-2.26	Cytokinin riboside 5'-monophosphate phosphoribohydrolase LOG5
659	TRINITY_DN30315_c0_g2	1129	102.46	4.01	21.95	0.79	-2.25	Guanine nucleotide-binding protein subunit beta-like protein
660	TRINITY_DN29314_c0_g2	1148	64.57	2.49	13.60	0.48	-2.25	High-affinity nitrate transporter 2.1
661	TRINITY_DN13251_c0_g2	2981	65.05	0.96	13.50	0.18	-2.25	Pentatricopeptide repeat-containing protein At1g03560, mitochondrial
662	TRINITY_DN3925_c0_g1	414	36.99	3.95	8.24	0.81	-2.23	Peroxidase 44
663	TRINITY_DN32471_c0_g3	1215	44.90	1.63	10.44	0.35	-2.20	Probable magnesium transporter NIPA4
664	TRINITY_DN36115_c0_g1	1075	5654.66	232.60	1269.68	48.13	-2.20	Transcription factor UPBEAT1
665	TRINITY_DN3695_c0_g2	1151	582.86	22.39	130.92	4.63	-2.19	PLAT domain-containing protein 2
666	TRINITY_DN30039_c0_g2	2056	36.39	0.78	7.76	0.15	-2.19	Pentatricopeptide repeat-containing protein At1g69290
667	TRINITY_DN35179_c2_g2	1177	1352.10	50.80	305.12	10.56	-2.19	Sesquiterpene synthase
668	TRINITY_DN26819_c0_g2	1009	70.92	3.11	16.14	0.65	-2.18	Probable amino-acid acetyltransferase NAGS2, chloroplastic
669	TRINITY_DN31413_c0_g2	1806	83.86	2.05	19.04	0.43	-2.18	BTB/POZ domain-containing protein At1g04390
670	TRINITY_DN14506_c0_g1	563	44.06	3.46	9.95	0.72	-2.16	Glucose-1-phosphate adenylyltransferase large subunit 3, chloroplastic
671	TRINITY_DN37686_c0_g1	581	44.10	3.36	10.14	0.71	-2.16	Endoglucanase 5
672	TRINITY_DN31828_c0_g2	1597	1149.00	31.81	265.21	6.77	-2.16	Cytosolic sulfotransferase 15
673	TRINITY_DN1330_c0_g1	2583	229.94	3.94	53.13	0.84	-2.16	Protease Do-like 2, chloroplastic
674	TRINITY_DN33648_c0_g1	921	60.55	2.91	14.48	0.64	-2.15	5-pentadecatrienyl resorcinol O-methyltransferase
675	TRINITY_DN3756_c0_g1	1017	104.16	4.53	23.57	0.94	-2.15	Peptidyl-prolyl cis-trans isomerase CYP21-1

*logFC: the logarithm to base 2 of fold change (Salt/Control)

**TPM: transcripts per million

Supplementary Table 1. (Cont) Annotation profile of DEG in ice plant seedlings treated with 200 mM NaCl (|FC|>4, FDR<0.001)

No.	Transcript ID	Length	Control reads	Control reads (TPM**)	Salt reads	Salt reads (TPM)	logFC*	Annotation
676	TRINITY_DN28882_c0_g1	2032	781.30	17.00	180.85	3.63	-2.15	Protein DEK
677	TRINITY_DN34751_c0_g1	732	89.94	5.43	20.83	1.16	-2.13	Nicotinamide/nicotinic acid mononucleotide adenylyltransferase
678	TRINITY_DN27560_c0_g1	1728	81.13	2.08	19.32	0.46	-2.13	Putative glucose-6-phosphate 1-epimerase
679	TRINITY_DN43278_c0_g1	1010	46.86	2.05	11.34	0.46	-2.12	Transcription factor bHLH85
680	TRINITY_DN54276_c0_g1	1440	101.67	3.12	23.59	0.67	-2.12	Probable receptor-like protein kinase At4g39110
681	TRINITY_DN25167_c0_g1	549	118.07	9.51	27.84	2.07	-2.11	NADH-ubiquinone oxidoreductase chain 2
682	TRINITY_DN19258_c0_g1	364	75.53	9.18	18.42	2.06	-2.11	PHD finger protein ALFIN-LIKE 5
683	TRINITY_DN53381_c0_g1	502	54.61	4.81	12.68	1.03	-2.11	Mavicyanin
684	TRINITY_DN22351_c0_g1	923	196.31	9.40	46.98	2.07	-2.10	NAD(P)H-quinone oxidoreductase subunit M, chloroplastic
685	TRINITY_DN28827_c0_g2	1380	49.65	1.59	11.50	0.34	-2.09	Transcription factor bHLH126
686	TRINITY_DN30071_c0_g3	2292	106.63	2.06	26.12	0.46	-2.08	tRNA-specific adenosine deaminase 2
687	TRINITY_DN31330_c0_g3	1358	70.48	2.29	16.85	0.51	-2.07	Protein OSB1, mitochondrial
688	TRINITY_DN32467_c0_g1	854	40.71	2.11	10.17	0.49	-2.06	Elongation of fatty acids protein 3-like
689	TRINITY_DN35030_c0_g3	1576	44.86	1.26	11.00	0.28	-2.06	Pentatricopeptide repeat-containing protein At1g31790
690	TRINITY_DN31232_c0_g3	1862	202.38	4.81	49.77	1.09	-2.05	3-oxoacyl-[acyl-carrier-protein] reductase, chloroplastic
691	TRINITY_DN28322_c0_g1	2292	101.23	1.95	25.45	0.45	-2.05	Putative ribonuclease H protein At1g65750
692	TRINITY_DN35267_c4_g1	2125	6918.63	143.97	1736.35	33.29	-2.04	Glucose-6-phosphate/phosphate translocator 2, chloroplastic
693	TRINITY_DN28827_c0_g1	1276	314.57	10.90	79.17	2.53	-2.03	Transcription factor bHLH126
694	TRINITY_DN33440_c1_g1	1647	55.78	1.50	14.44	0.36	-2.03	Zinc finger CCCH domain-containing protein 12
695	TRINITY_DN44593_c0_g1	756	52.22	3.05	13.09	0.71	-2.03	Fructose-1,6-bisphosphatase, cytosolic
696	TRINITY_DN41843_c0_g1	747	48.08	2.85	11.94	0.65	-2.03	Rop guanine nucleotide exchange factor 12
697	TRINITY_DN27211_c0_g1	451.74	43.50	4.25	11.46	0.93	-2.03	Aldo-keto reductase family 4 member C10
698	TRINITY_DN11859_c0_g1	541	43.79	3.58	10.59	0.80	-2.03	Dehydrodolichyl diphosphate synthase 6
699	TRINITY_DN6988_c0_g1	1024	39.52	1.71	10.46	0.42	-2.03	Stilbene synthase 3
700	TRINITY_DN32850_c0_g2	2798	158.44	2.50	40.47	0.59	-2.02	Bromodomain and PHD finger-containing protein 3

*logFC: the logarithm to base 2 of fold change (Salt/Control)

**TPM: transcripts per million

Supplementary Table 1. (Cont) Annotation profile of DEG in ice plant seedlings treated with 200 mM NaCl (|FC|>4, FDR<0.001)

No.	Transcript ID	Length	Control reads	Control reads (TPM**)	Salt reads	Salt reads (TPM)	logFC*	Annotation
701	TRINITY_DN12977_c0_g1	1545	74.74	2.14	19.35	0.51	-2.01	Probable transcriptional regulatory protein At2g25830
702	TRINITY_DN35326_c7_g3	1295.68	122.34	4.16	31.19	0.98	-2.01	Transposon Ty3-I Gag-Pol polyprotein
703	TRINITY_DN29438_c0_g2	882	51.09	2.56	12.98	0.60	-2.00	Ethylene-responsive transcription factor ERF113
704	TRINITY_DN34739_c1_g1	665	848.37	56.41	218.37	13.38	-2.00	Probable xyloglucan endotransglucosylase/hydrolase protein 26

*logFC: the logarithm to base 2 of fold change (Salt/Control)

**TPM: transcripts per million
