# Supplementary material for: Transcriptomic and enzymatic analysis of peroxidase families at the early growth stage of halophyte ice plant (Mesembryanthemum crystallinum L.) under salt stress
Source: Bot Stud. 2025 Jan 21;66:5. doi: 10.1186/s40529-024-00450-y (PMC11751343; doi:10.1186/s40529-024-00450-y)
Supplement: Supplementary file 2 — Supplementary Material 2 [file 40529_2024_450_MOESM2_ESM.pdf]

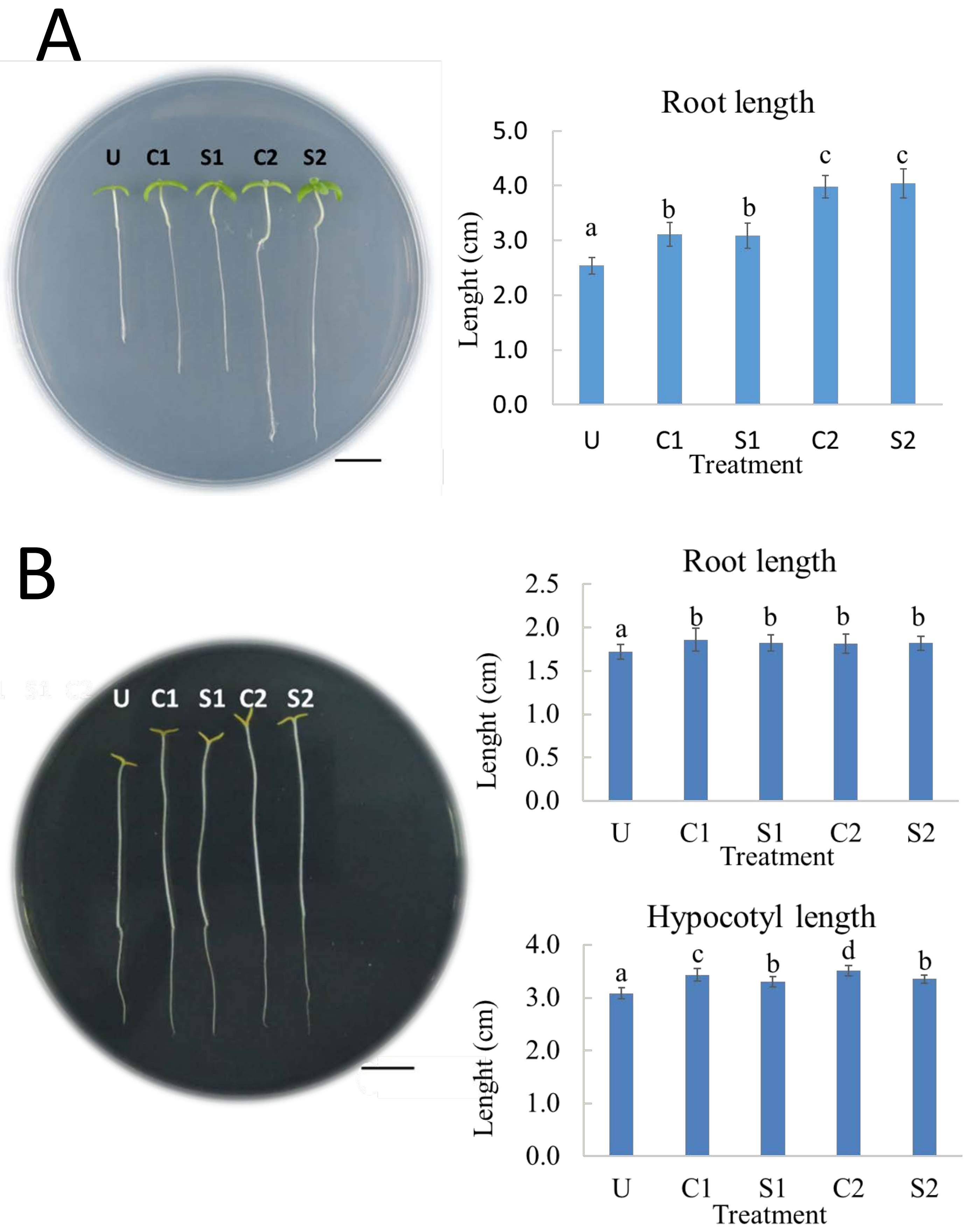

Supplementary Fig 1. The growth of ice plant seedlings treated with 0 or 200 mM NaCl for 2 days. Five-day-old light-grown seedlings (A) and 3-day-old etiolated seedlings (B) were treated with 0 mM (control, C) or 200 mM NaCl (salt, S). Samples were collected on day 1 (C1 and S1) and day 2 (C2 and S2). Rapid root growth following the addition of fresh liquid MS medium was observed in light-grown seedlings, consistent with previous reports (Chiang et al., 2016; Tsukagoshi et al., 2015). U: untreated seedlings. The lengths of the root and hypocotyl at each time point were measured from 15 seedlings. Error bars were calculated using the Excel STDEVP function. Different letters indicate significant differences at $P < 0.05$. Bar = 1 cm.

Chiang CP, Yim WC, Sun YH, Ohnishi M, Mimura T, Cushman JC, Yen HE (2016) Identification of ice plant (*Mesembryanthemum crystallinum* L.) microRNAs using RNA-Seq and their putative roles in high salinity responses in seedlings. Front Plant Sci 7:1143.

Tsukagoshi H, Suzuki T, Nishikawa K, Agarie S, Ishiguro S, Higashiyama T (2015) RNA-seq analysis of the response of the halophyte, *Mesembryanthemum crystallinum* (ice plant) to high salinity. PLoS One 10:e0118339.

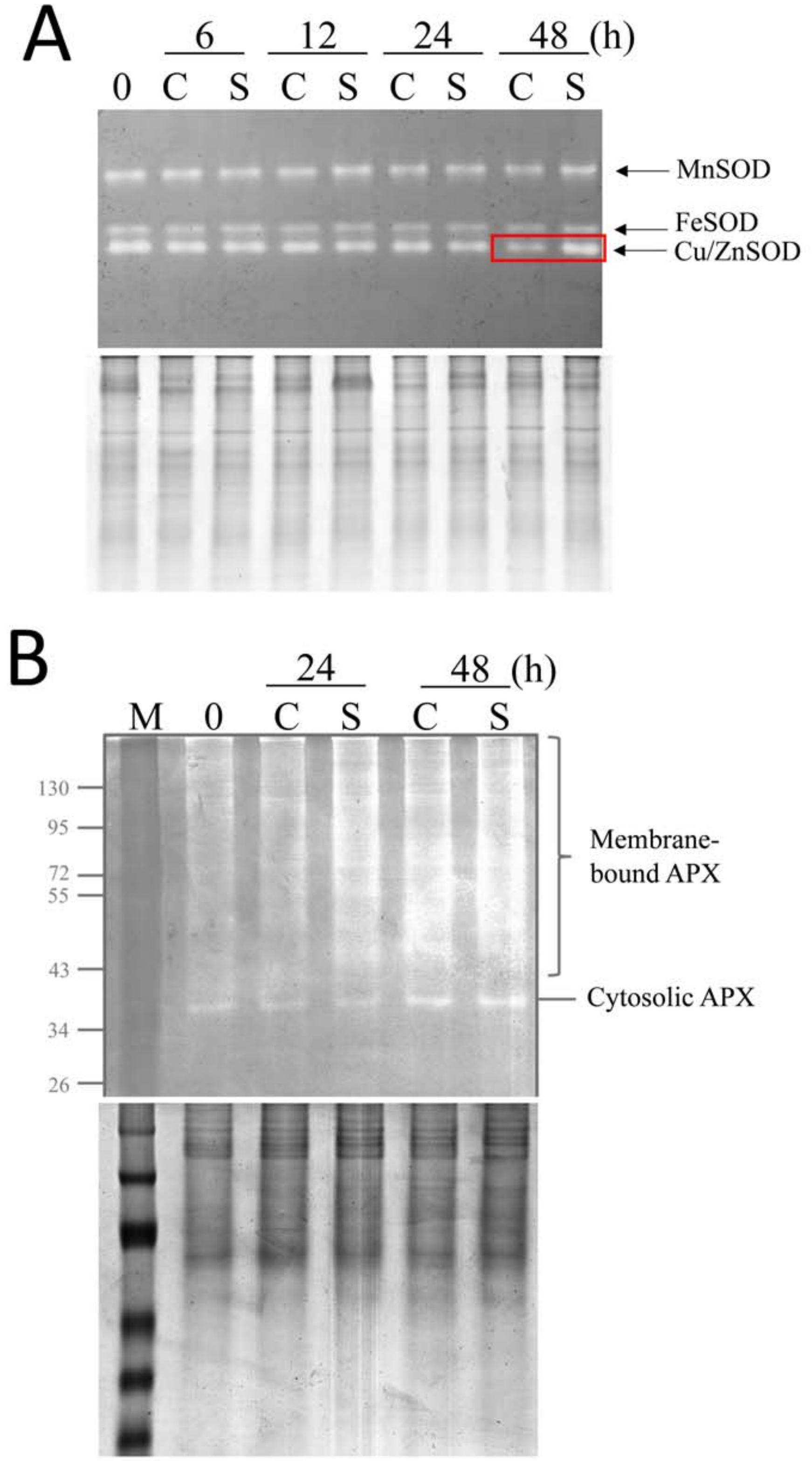

Supplementary Fig 2. Activity staining of superoxide dismutase (A) and ascorbate peroxidase (B) in native PAGE. Five-day-old seedlings were treated with 0 (control; C) or 200 mM NaCl (salt; S) for 48 h. Sample preparation and SOD and APX activity staining was performed according to Miszalski et al. (1998) and Mittler & Zilinskas (1993), respectively. The upper panel is the activity staining showing SOD (A) or APX (B) activity and the lower panel is the Coomassie blue staining showing equal loading. Arrows indicate the position of enzyme activity. The red box indicates salt-induced Cu/Zn SOD activity at 48 h.

Mittler R, Zilinskas BA (1993) Detection of ascorbate peroxidase activity in native gels by inhibition of the ascorbate-dependent reduction of nitroblue tetrazolium. Anal Biochem 212:540-546.

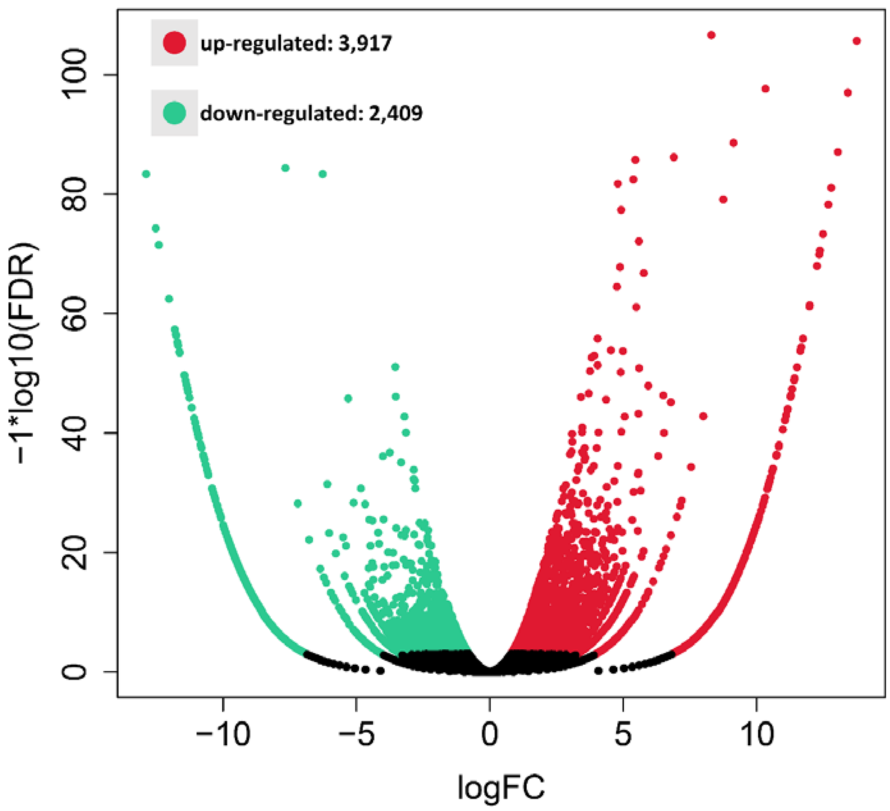

Supplementary Fig 3. A volcano plot showing the differentially expressed genes (DEGs) of ice plant seedlings treated with 0 mM or 200 mM NaCl for 6 h. The biological significance (log_2 Fold Change) is at the *x*-axis and statistical significance (log_{10} FDR) is at the *y*-axis. Statistical significance was corrected at FDR<0.001. Transcripts with increased expression are marked with red, and transcripts with decreased expression are marked with green.

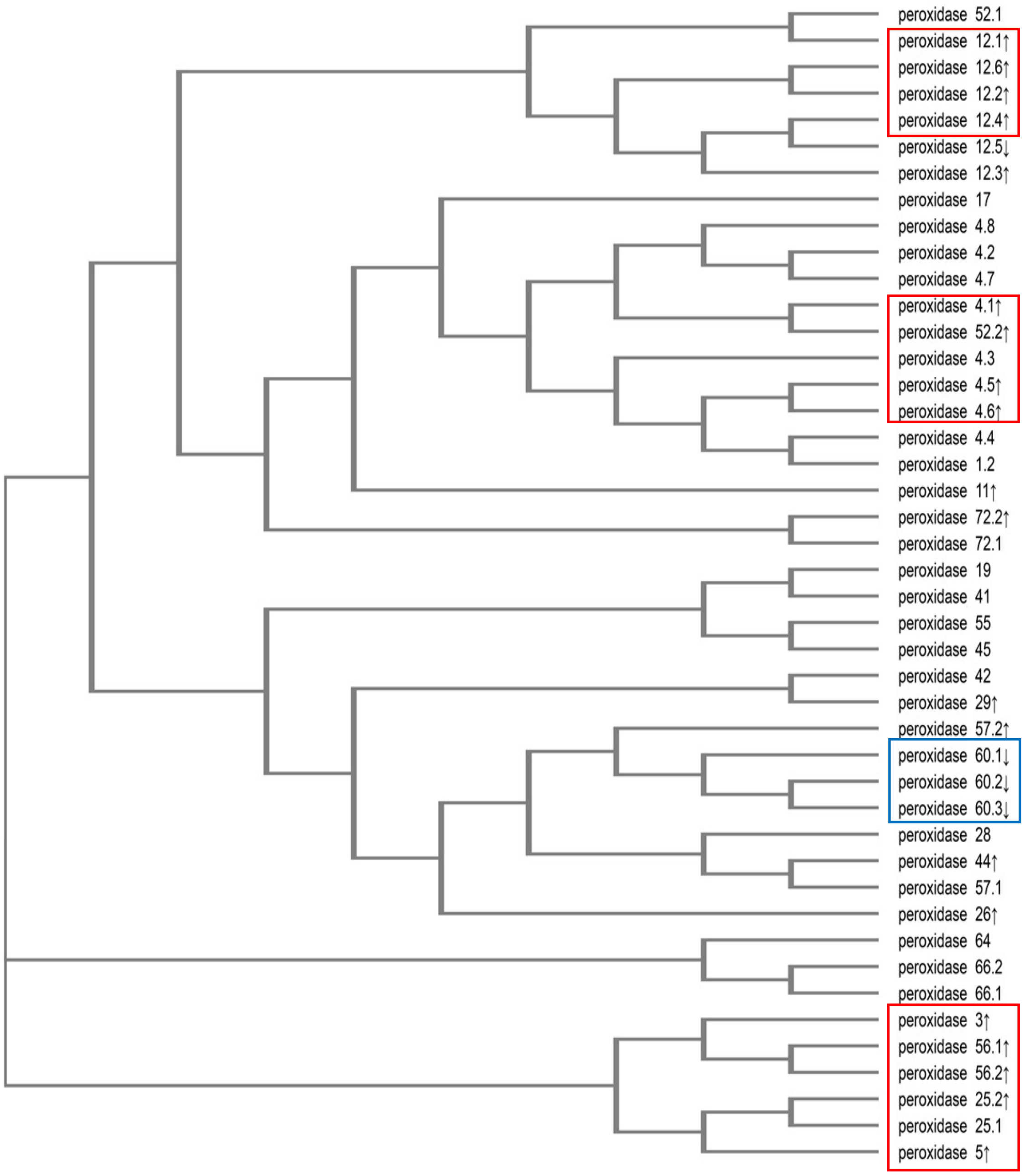

Supplementary Figure 4. Phylogenetic analysis of ice plant peroxidase gene family and regulation by salt. Upregulated transcripts mark with ↑; downegulated transcripts mark with ↓. Red boxes show cluster of genes showing upregulation, while the blue box shows downregulation of peroxidase 60 family members. The transcripts were aligned to the peroxidase genes in the ice plant transcriptome provided by Dr. Cushman.
